# TBK1 eliminates aggregation-prone monomeric TDP-43 through an IFNβ-immunoproteasome pathway in ALS models

**DOI:** 10.1038/s44319-026-00953-8

**Published:** 2026-10-05

**Authors:** Shohei Sakai, Kotaro Oiwa, Yohei Iguchi, Izzet Mehmet Akcay, Seiji Watanabe, Okiru Komine, Noriko Nagata, Mai Horiuchi, Jemeen Sreedharan, Masahisa Katsuno, Koji Yamanaka

**Affiliations:** 1 Department of Neuroscience and Pathobiology, Research Institute of Environmental Medicine, Nagoya University, Nagoya, Aichi 464-8601 Japan; 2 Department of Neuroscience and Pathobiology, Nagoya University Graduate School of Medicine, Nagoya, Aichi 466-8560 Japan; 3 Laboratory of Genetics, The Salk Institute for Biological Studies, La Jolla, CA 92037 USA; 4 Department of Neurology, Nagoya University Graduate School of Medicine, Nagoya, Aichi 466-8550 Japan; 5 Department of Basic and Clinical Neuroscience, The Maurice Wohl Clinical Neuroscience Institute, Institute of Psychiatry, Psychology and Neuroscience (IoPPN), King’s College London, London, SE5 9RT UK; 6 Department of Life Science, Faculty of Medicine, Shimane University, Izumo, Shimane 693-8501 Japan; 7 Institute for Glyco-core Research (iGCORE), Nagoya University, Nagoya, Aichi Japan; 8 Center for One Medicine Innovative Translational Research (COMIT), Nagoya University, Nagoya, Aichi Japan; 9 Research Institute for Quantum and Chemical Innovation, Institutes of Innovation for Future Society, Nagoya University, Nagoya, Aichi Japan

**Keywords:** Molecular Biology of Disease, Neuroscience, Post-translational Modifications & Proteolysis

## Abstract

Loss-of-function mutations in TANK-binding kinase 1 (TBK1) cause amyotrophic lateral sclerosis (ALS) and frontotemporal dementia (FTD), characterized by cytoplasmic aggregation of TAR DNA-binding protein 43 (TDP-43), known as TDP-43 pathology, but the mechanisms beyond impaired autophagy remain undefined. Here, we identify a TBK1-IFNβ-immunoproteasome axis as a novel autophagy-independent proteostatic pathway regulating TDP-43 clearance. TBK1 is activated by aggregation-prone monomeric TDP-43 via physical association, and this activation alleviates TDP-43 pathology in neuronal cells. TBK1 subsequently induces IFNβ expression, which upregulates the immunoproteasome to promote degradation of monomeric TDP-43 in neurons, including human iPSC-derived lower motor neurons. Importantly, IFNβ receptor expression is downregulated in spinal motor neurons from ALS patients with TDP-43 pathology. Furthermore, heterozygous *Tbk1* deletion in *SOD1*^G93A^ mice impairs immunoproteasome induction and increases polyubiquitinated protein accumulation in spinal cords, supporting the in vivo relevance of this pathway. Collectively, our findings reveal that impairment of the TBK1-IFNβ-immunoproteasome axis represents an autophagy-independent mechanism contributing to the development of TDP-43 pathology in ALS and FTD.

## Synopsis

TBK1 activation by aggregation-prone monomeric TDP-43 induces IFNβ and the immunoproteasome, promoting the clearance of monomeric TDP-43 independently of autophagy. Impairment of this TBK1–IFNβ– immunoproteasome axis may contribute to TDP-43 pathology in ALS and FTD.

- Aggregation-prone monomeric TDP-43 activates TBK1 via physical association, which induces IFNβ and the immunoproteasome to promote monomeric TDP-43 clearance in neurons.
- IFNβ receptor expression is downregulated in spinal motor neurons with TDP-43 pathology from patients with ALS.
- Heterozygous Tbk1 deletion impairs immunoproteasome induction and increases polyubiquitinated protein accumulation in the spinal cords of an SOD1-ALS mouse model.

## Abstract (web summary)

TBK1 activation by aggregation-prone monomeric TDP-43 induces IFNβ and the immunoproteasome, promoting the clearance of monomeric TDP-43 independently of autophagy. Impairment of this TBK1–IFNβ– immunoproteasome axis may contribute to TDP-43 pathology in ALS and FTD.

## Graphical abstract

**Figure:**
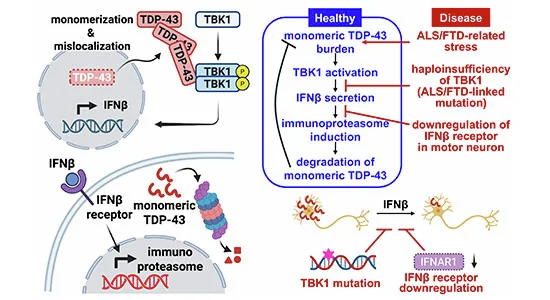

## Introduction

Cytoplasmic mislocalization and abnormal accumulation of TAR DNA-binding protein 43 (TDP-43) in the brain and spinal cord, known as TDP-43 pathology, are pathological hallmarks in several incurable neurodegenerative diseases such as amyotrophic lateral sclerosis (ALS) and frontotemporal dementia (FTD) (Arai et al, 2006; Neumann et al, 2006). TDP-43 is an RNA-binding protein that regulates multiple aspects of RNA metabolism, such as RNA transcription, splicing, transport, and translation (Lagier-Tourenne et al, 2010). As TDP-43 regulates numerous transcripts essential for neuronal health and function (Polymenidou et al, 2011; Klim et al, 2019; Melamed et al, 2019; Brown et al, 2022; Ma et al, 2022), the cytoplasmic aggregation of TDP-43, along with the loss of nuclear TDP-43, is considered a primary driver of neurodegeneration. Therefore, preventing TDP-43 pathology represents a rational therapeutic strategy for ALS and FTD. However, the precise molecular mechanisms underlying TDP-43 pathology have not been fully understood.

Mutations in *TBK1*, encoding TANK-binding kinase 1 (TBK1), cause familial ALS/FTD with TDP-43 pathology through loss-of-function mechanisms (Cirulli et al, 2015; Freischmidt et al, 2015). We recently reported that active TBK1 levels are reduced in the brains and spinal cords of sporadic ALS patients (Watanabe et al, 2023), suggesting that deregulated TBK1 broadly contributes to ALS/FTD pathogenesis and TDP-43 pathology, including sporadic cases. TBK1 is a multifunctional kinase involved in proteostasis and inflammation (Oakes et al, 2017; Ahmad et al, 2016). In the autophagy pathway, TBK1 phosphorylates autophagy receptors Optineurin (Wild et al, 2011) and p62 (Pilli et al, 2012) and promotes autophagic clearance of damaged organelles and protein aggregates. Because both *OPTN* and *SQSTM1* have been identified as ALS/FTD causative genes (Maruyama et al, 2010; Fecto et al, 2011; Le Ber et al, 2013; Pottier et al, 2018), there is growing consensus that deregulation of the autophagy–lysosome pathway is one of the main causes of ALS/FTD pathogenesis. On the other hand, in innate immune signaling, TBK1 regulates inflammation by phosphorylating stimulator of interferon genes (STING) (Liu et al, 2015), nuclear factor-kappa B (NF-κB) (Buss et al, 2004), and interferon regulatory factor 3 (IRF3) (Tanaka and Chen, 2012) to induce the expression of proinflammatory cytokines such as tumor necrosis factor (TNF) and type I interferons (IFNs) such as IFNβ. In ALS/FTD studies on TBK1, the autophagy–lysosome pathway has been well-studied; however, the effects of TBK1-induced cytokines on TDP-43 pathology in neurons remain poorly understood.

Here, we propose a novel mechanism by which TBK1 mediates abnormal TDP-43 clearance. In this study, we found that TBK1 regulates proteostasis by inducing IFNβ and the immunoproteasome through a multidimensional approach. Furthermore, impairment of the TBK1-IFNβ-immunoproteasome axis was observed in *Tbk1*-deficient ALS-SOD1 mice and the spinal motor neurons of ALS patients.

## Results

### TBK1 is activated by monomeric TDP-43 and alleviates TDP-43 pathology in cells

**Figure 1:**
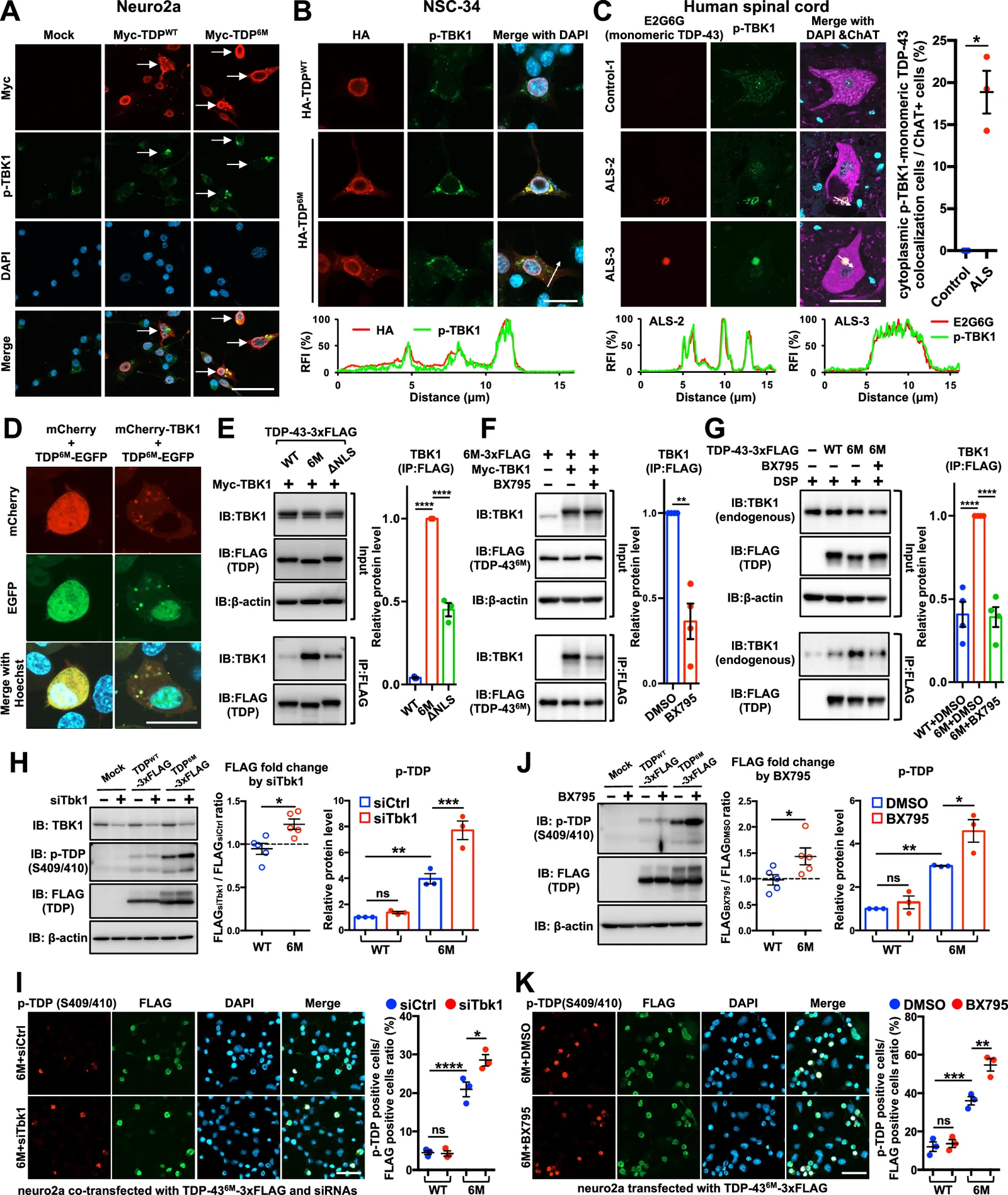
TBK1 is activated by monomeric TDP-43 via a physical association and alleviates TDP-43 pathology in cells. (**A**,** B**) Cytoplasmic monomeric TDP-43 induces TBK1 phosphorylation via a physical association. Neuro2a (**A**) and NSC-34 (**B**) cells were transiently transfected with human TDP-43^WT^ or TDP-43^6M^ expression vectors and analyzed by immunocytochemistry with the indicated antibodies. Representative confocal images from two independent experiments are shown. Relative fluorescence intensity (RFI) of each channel at points along the white arrow is shown. Scale bars, 50 μm (**A**) or 20 μm (**B**). (**C**) Monomeric TDP-43 colocalizes with phosphorylated TBK1 (p-TBK1) in spinal motor neurons from ALS patients. Representative images show p-TBK1 and monomeric TDP-43 (E2G6G) immunostaining with a spinal motor neuron marker ChAT in the spinal cords from control subjects and ALS patients. Spinal cord sections from three control subjects and three ALS patients with TDP-43 pathology were analyzed. The proportion of motor neurons showing cytoplasmic colocalization of p-TBK1 and monomeric TDP-43 was quantified and plotted (right graph). Colocalization was observed in 4/28 (14.3%), 3/13 (23.1%), and 5/26 (19.2%) motor neurons from ALS-1, ALS-2, and ALS-3, respectively. No motor neurons from the control subjects showed cytoplasmic colocalization (0/22, 0/21, and 0/18 motor neurons from Control-1, Control-2, and Control-3, respectively). *P* values: *p* = 0.0177 (*). RFI of each channel at points along the white arrows is shown (bottom graphs). Scale bar, 50 μm. (**D**) TBK1 colocalizes with monomeric TDP-43 in live cells. Neuro2a cells were transiently co-transfected with human TDP-43^6M^-EGFP and either mCherry or mCherry-TBK1 expression vectors and analyzed by live-cell imaging. Representative confocal images from three independent experiments are shown. Scale bar, 20 μm. (**E**,** F**) Overexpressed TBK1 interacts with monomeric TDP-43 in a kinase activity-dependent manner. Neuro2a cells were transiently co-transfected with human TDP-43-3×FLAG constructs (WT, 6M, or ΔNLS) together with either mock or Myc-TBK1 expression vectors, followed by immunoprecipitation with an anti-FLAG antibody and subsequent immunoblotting. Representative immunoblotting data were shown, and relative TBK1 levels in the IP:FLAG panels are quantified and plotted (right graphs). *n* = 3–4, biologically independent experiments. In (**F**), cells were treated with either DMSO or 1 μM BX795 (TBK1-specific inhibitor) following transfection. *P* values: *p* < 0.0001 (****), *p* = 0.0094 (**). (**G**) Endogenous TBK1 interacts with monomeric TDP-43. Neuro2a cells were transiently transfected with human TDP-43-3×FLAG constructs (WT or 6M) and treated with a cell-permeable crosslinker, dithiobis(succinimidyl propionate) (DSP), followed by immunoprecipitation with an anti-FLAG antibody and subsequent immunoblotting. Representative immunoblotting data are shown, and relative TBK1 levels in the IP:FLAG panels are quantified and plotted (right graph). *n* = 4, biologically independent experiments. *P* values: *p* < 0.0001 (****). (**H**,** I**) Knockdown of *Tbk1* exacerbates TDP-43 pathology driven by monomeric TDP-43 in cells. Neuro2a cells were co-transfected with the indicated plasmids and either control siRNA (siCtrl) or siRNA against *Tbk1* (siTbk1), cultured in differentiation medium, and subsequently analyzed by immunoblotting (**H**) or immunocytochemistry (**I**). Representative immunoblotting images with quantified FLAG fold changes and relative p-TDP levels are shown (**H**). *n* = 3–5, biologically independent experiments. *P* values: *p* = 0.0113 (*) for FLAG fold change, *p* = 0.9235 (ns), 0.0042 (**), 0.0009 (***) for p-TDP. Representative immunocytochemistry images are shown, with the ratio of p-TDP-positive to FLAG-positive cells quantified and plotted (**I**). *n* = 3, biologically independent experiments. *P* values: *p* = 0.999 (ns), 0.013 (*), *p* < 0.0001 (****). In each experiment, ~200–300 FLAG-positive cells per condition were analyzed. Scale bar, 50 μm. (**J**,** K**) Pharmacological inhibition of TBK1 exacerbates TDP-43 pathology driven by monomeric TDP-43 in cells. Neuro2a cells were transfected with the indicated plasmids and treated with either DMSO or 1 μM BX795, cultured in differentiation medium, and subsequently analyzed by immunoblotting (**J**) or immunocytochemistry (**K**). Representative immunoblotting images with quantified FLAG fold changes and relative p-TDP levels are shown (**J**). *n* = 3–5, biologically independent experiments. *P* values: *p* = 0.0494 (*) for FLAG fold change, *p* = 0.9028 (ns), 0.0212 (*), 0.0071 (**) for p-TDP. Representative immunocytochemistry images of the TDP-43^6M^ cells treated with either DMSO or BX795 are shown. The ratio of p-TDP-positive to FLAG-positive cells is quantified and plotted (**K**). *n* = 3, biologically independent experiments. *P* values: *p* = 0.9669 (ns), 0.0035 (**), 0.0007 (***). In each experiment, ~200–300 FLAG-positive cells per condition were analyzed. Scale bar, 50 μm. All data were expressed as means ± SEM. Unpaired *t*-test with Welch’s correction [**C**, **F**, **H** (left graph), and **J** (left graph)] and one-way ANOVA followed by Holm–Sidak’s multiple comparisons test [**E**, **G**, **H** (right graph), **I**, **J** (right graph), and **K**]. ns, not significant. Source data are available online for this figure.

We recently reported that the monomerization of TDP-43 is a key determinant of TDP-43 pathology in sporadic ALS cases, and that expression of aggregation-prone monomeric TDP-43 mutants, such as 6M, a mutant carrying six point mutations at the homo-dimerization/oligomerization interface of TDP-43 (Afroz et al, 2017), recapitulates key characteristics of TDP-43 pathology, including cytoplasmic mislocalization and aggregation (Oiwa et al, 2023). We hypothesized that if TBK1 is crucial for suppressing TDP-43 pathology, it should be activated in response to monomeric TDP-43 burden. First, we found that phosphorylated TBK1 (p-TBK1), the active form of TBK1, was increased in cells exhibiting cytoplasmic TDP-43, particularly in cells transfected with the 6M mutant (Fig. 1A), and that p-TBK1 colocalized with cytoplasmic monomeric TDP-43 (Fig. 1B). Immunocytochemistry using a monomeric TDP-43-specific antibody (E2G6G) (Oiwa et al, 2023) and the FlexAble technology also revealed colocalization of these two proteins (Appendix Fig. S1). Importantly, spinal motor neurons from sporadic ALS patients also showed colocalization of monomeric TDP-43 and p-TBK1, providing the first in vivo evidence of a TDP-43–TBK1 physical association (Fig. 1C; Appendix Fig. S2). Additionally, we demonstrated that overexpressed TBK1 interacts with monomeric TDP-43 by live-cell imaging (Fig. 1D; Appendix Fig. S3) and co-immunoprecipitation (co-IP) (Fig. 1E) in a kinase activity-dependent manner (Fig. 1F). Dithiobis(succinimidyl propionate) (DSP) crosslinking followed by co-IP demonstrated that monomeric TDP-43 also interacts with endogenous TBK1 (Fig. 1G). Reducing endogenous TBK1 activity by siRNA-mediated knockdown or treatment with the TBK1-specific inhibitor BX795 increased levels of monomeric TDP-43 and its phosphorylated form (Fig. 1H–K). Collectively, these data suggest that TBK1 is activated by monomeric TDP-43 via a physical association and alleviates TDP-43 pathology in cells.

### TBK1 reduces aggregation-prone monomeric TDP-43 via humoral factors

**Figure 2:**
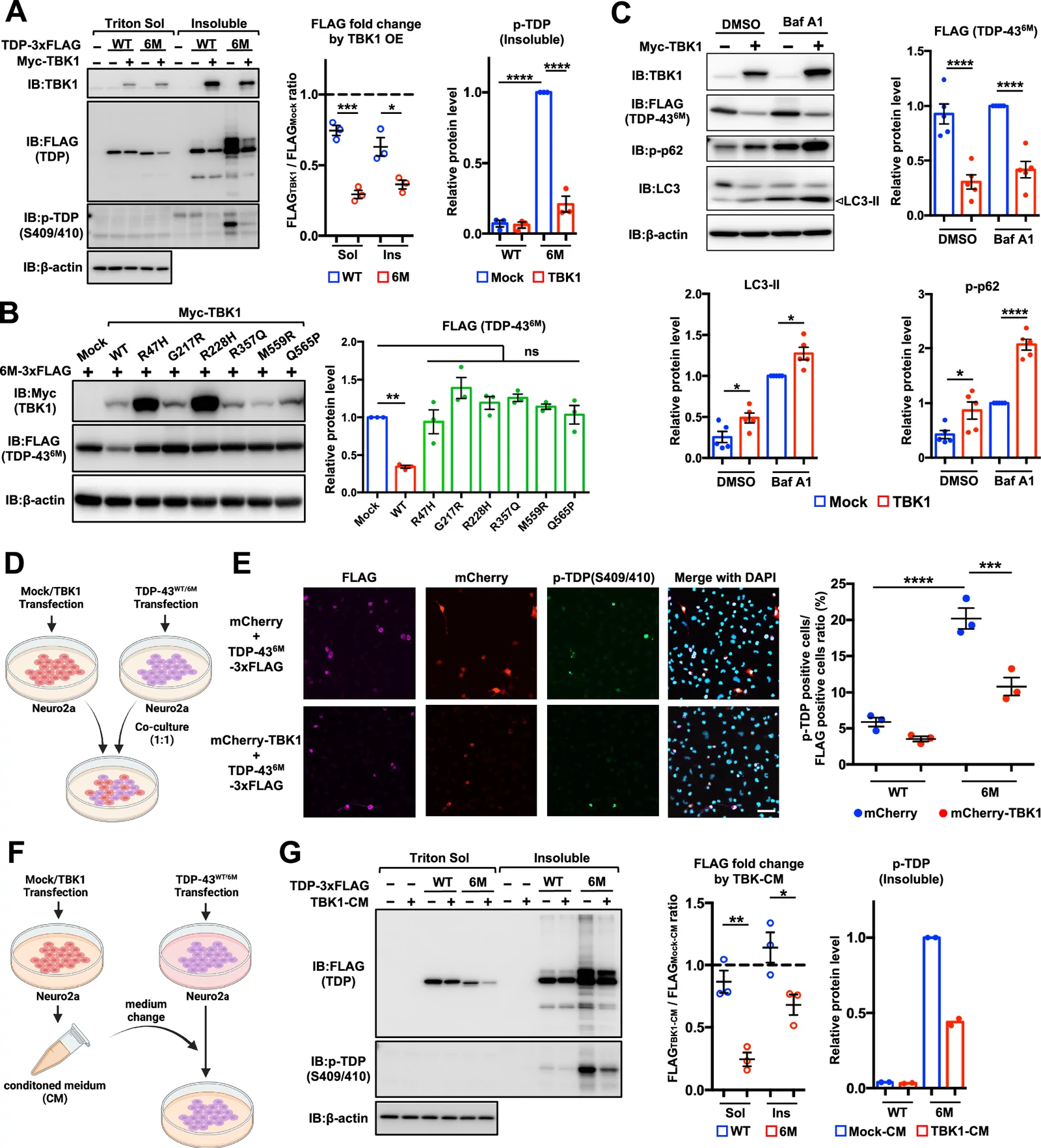
TBK1 overexpression reduces monomeric TDP-43 accumulation in cells via humoral factors. (**A**) TBK1 overexpression reduced the levels of transfected human TDP-43, particularly the monomeric TDP-43 mutant, as well as its phosphorylated form (p-TDP). Neuro2a cells were transiently co-transfected with human TDP-43^WT^ or TDP-43^6M^-3×FLAG and either mock or human Myc-TBK1 expression vectors. The cell lysates were then fractionated into 1% Triton X-100-soluble (Sol) and -insoluble (Ins) fractions and subjected to immunoblot analysis. Representative immunoblotting images with quantified FLAG fold changes and relative p-TDP levels are shown. *n* = 3, biologically independent experiments. *P* values: *p* = 0.0366 (*), 0.0007 (***) for FLAG fold change, *p* < 0.0001 (****) for p-TDP. (**B**) ALS/FTD-linked kinase-dead TBK1 mutants failed to reduce monomeric TDP-43 levels.　Neuro2a cells were transiently co-transfected with TDP-43^6M^-3×FLAG and the indicated expression vectors of wild-type or mutant Myc-TBK1. Representative immunoblotting data as well as the relative expression level of FLAG (TDP-43^6M^) are shown. *n* = 3, biologically independent experiments. *P* values: *p* = 0.0034 (**) for WT, 0.9792 (ns) for R47H, 0.1712 (ns) for G217R, 0.8835 (ns) for R228H, 0.6907 (ns) for R357Q, 0.9594 (ns) for M559R, 0.9792 (ns) for Q565P. (**C**) Inhibition of the autophagy–lysosome pathway did not abolish the TBK1-mediated reduction of monomeric TDP-43.　Neuro2a cells were transiently co-transfected with TDP-43^6M^-3×FLAG and either mock or Myc-TBK1 expression vectors, treated with either DMSO or 100 nM bafilomycin A1 (Baf A1) for 20 h, and analyzed by immunoblotting. Representative immunoblotting data, as well as the relative expression levels of FLAG, LC3-II, and phospho-p62, are shown. *n* = 5, biologically independent experiments. *P* values: *p* = 0.0141 (*) for LC3-II DMSO, 0.0105 (*) for LC3-II Baf A1, 0.015 (*) for p-p62 DMSO, *p* < 0.0001 (****). (**D**,** E**). TDP-43 pathology was suppressed by neighboring cells overexpressing TBK1. The experimental scheme is illustrated in (**D**). Representative immunofluorescence images of co-cultured Neuro2a cells from the TDP-43^6M^/TBK1 transfection experiment are shown in (**E**). The ratio of p-TDP-positive to FLAG-positive cells is quantified and plotted (right). *n* = 3, biologically independent experiments. *P* values: *p* = 0.0008 (***), *p* < 0.0001 (****). In each experiment, more than 200 FLAG-positive cells per condition were analyzed. Scale bar, 50 μm. (**F**,** G**). Conditioned medium (CM) from TBK1-overexpressing cells reduced monomeric TDP-43 and p-TDP levels in CM-recipient cells. The experimental scheme is illustrated in (**F**). Lysates from CM-recipient cells were fractionated as in (**A**) and analyzed by immunoblotting. Representative immunoblotting images with quantified FLAG fold changes and relative p-TDP levels are shown. *n* = 3, biologically independent experiments. *P* values: *p* = 0.0413 (*), 0.0073 (**). All data were presented as means ± SEM. Unpaired *t*-test with Welch’s correction [**A** (left graph) and **G** (left graph)] and one-way ANOVA followed by Holm–Sidak’s multiple comparisons test [**A** (right graph), **B**, **C**, **E**]. ns, not significant. Source data are available online for this figure.

In contrast to the results of endogenous TBK1 suppression, we found that TBK1 overexpression reduced TDP-43 levels in both the soluble and insoluble fractions, with a more pronounced effect on TDP-43^6M^ than on TDP-43^WT^ (Fig. 2A). TBK1 also decreased the levels of phosphorylated TDP-43 (p-TDP) induced by the monomeric mutant. We confirmed that TBK1 overexpression did not affect the mRNA levels of the transfected TDP-43 (Appendix Fig. S4). The reduction of TDP-43^6M^ by TBK1 overexpression was dependent on its kinase activity, as ALS/FTD-linked kinase-dead TBK1 mutants failed to reduce TDP-43^6M^ levels (Fig. 2B). Since TBK1 is involved in autophagy, we initially hypothesized that the substantial decrease in monomeric TDP-43 was dependent on the autophagy–lysosome pathway. To test this hypothesis, cells co-transfected with TDP-43^6M^ and TBK1 were treated with bafilomycin A1, a potent inhibitor of the autophagy–lysosome pathway. TBK1 increased the levels of phosphorylated p62 (p-p62) and LC3-II, indicating the activation of autophagy by TBK1. However, TDP-43^6M^ levels were still reduced by TBK1 even with bafilomycin A1 treatment (Fig. 2C). The increased LC3-II levels in the 3rd and 4th lanes indicate successful inhibition of autophagy–lysosomal degradation. Interestingly, proteasome inhibition by MG132 blocked the reduction of TDP-43^6M^ by TBK1 (Appendix Fig. S5). These results suggest that TBK1 has the potential to ameliorate TDP-43 pathology by reducing excess TDP-43, particularly the aggregation-prone monomeric form, through a mechanism independent of the autophagy–lysosome pathway. Next, we investigated the possibility that TBK1 suppresses TDP-43 pathology via autocrine and paracrine signaling, because TBK1 is involved in cytokine secretion. Co-culture experiments (Fig. 2D), followed by immunoblotting (Appendix Fig. S6A) and immunocytochemistry (Fig. 2E), revealed that neighboring cells overexpressing TBK1 reduced the protein levels of TDP-43^6M^ and p-TDP. To rule out the effects of physical cell-to-cell contact in the co-culture system, we conducted an additional experiment using conditioned medium (CM) (Fig. 2F). Consistently, CM derived from TBK1-overexpressing cells also reduced levels of TDP-43^6M^ and p-TDP (Fig. 2G; Appendix Fig. S6B). Collectively, these findings suggest that TBK1 ameliorates TDP-43 pathology via humoral factors such as cytokines.

### TBK1-IFNβ pathway is crucial for the clearance of monomeric TDP-43

**Figure 3:**
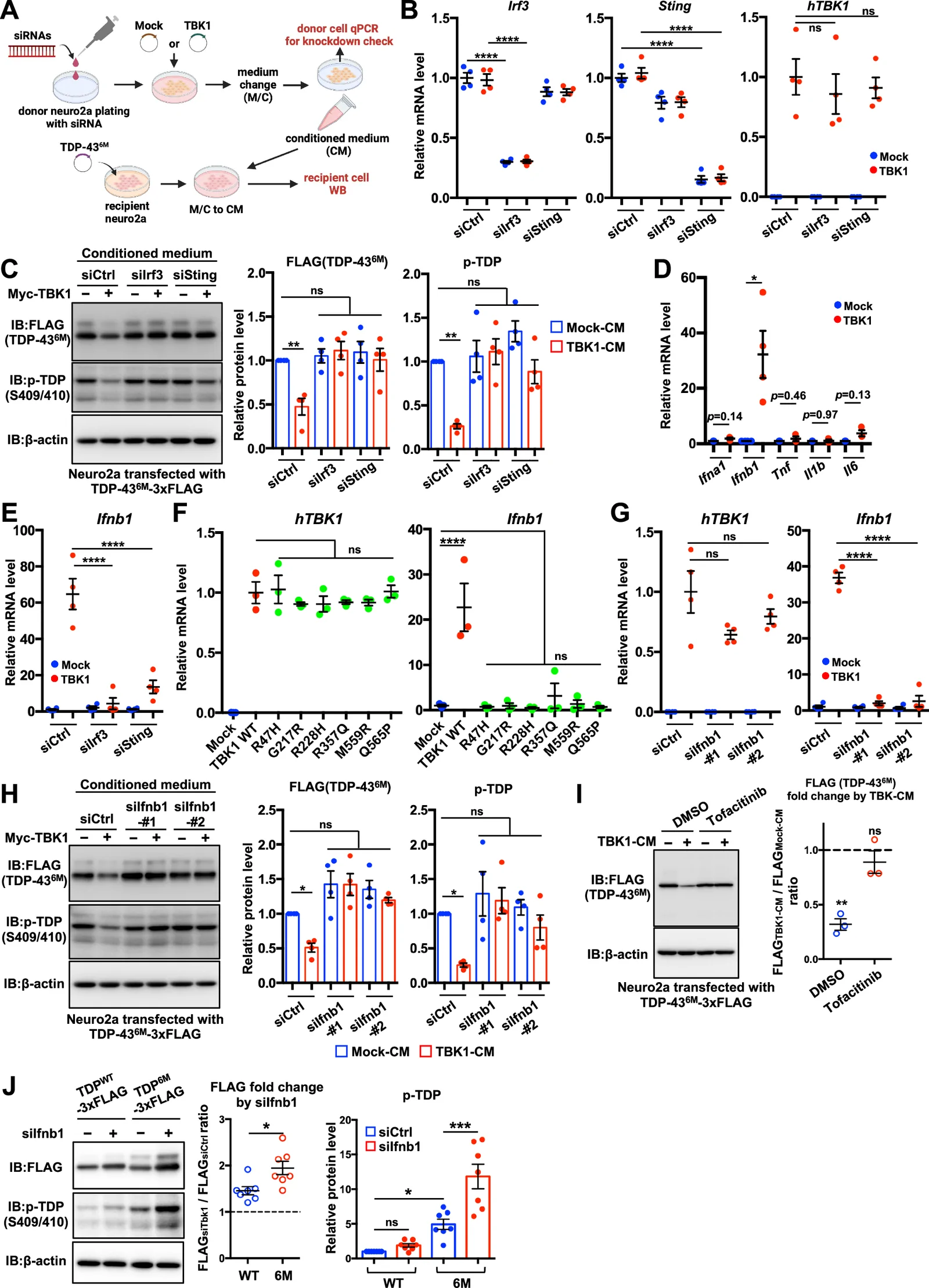
The STING-IRF3-IFNβ pathway is responsible for the clearance of monomeric TDP-43 in cells. (**A**) A schematic illustration of experiments using siRNAs and conditioned medium (CM). (**B**,** C**) Knockdown of *Irf3* or *Sting* abolished the reduction of the monomeric TDP-43 by TBK1-CM. Neuro2a cells (CM-donor cells) were treated with the indicated siRNAs, and then transfected with mock or TBK1 expression plasmids. Efficient knockdown of *Irf3* and *Sting* mRNA, without affecting *hTBK1* mRNA levels, was confirmed by qPCR in CM-donor cells (**B**). *P* values: *p* < 0.0001 (****), *p* = 0.9021 (ns) for siIrf3, 0.9746 (ns) for siSting. Cells treated with the indicated CM were analyzed by immunoblotting. Representative immunoblotting images (left) and relative protein levels of FLAG and p-TDP (right) are shown (**C**). *n* = 4, biologically independent experiments. *P* values: *p* = 0.0067 (**) for FLAG, 0.002 for p-TDP. (**D**) TBK1 overexpression in Neuro2a cells markedly induced IFNβ (*Ifnb1*). The expression levels of the indicated genes were analyzed in cells transfected with mock or TBK1 using qPCR. *n* = 4, biologically independent experiments. *P* values: *p* = 0.0352 (*). (**E**) qPCR revealed that knockdown of either *Sting* or *Irf3* markedly suppressed *Ifnb1* induction by TBK1 in Neuro2a cells. Cells treated with the indicated siRNAs were transfected with mock or TBK1. *Ifnb1* mRNA levels relative to control cells co-transfected with mock vector and siCtrl were determined by qPCR. *n* = 4, biologically independent experiments. *P* values: *p* < 0.0001 (****). (**F**) ALS/FTD-linked kinase-dead TBK1 mutants failed to induce IFNβ. NSC-34 cells were transiently transfected with the indicated expression vectors of wild-type or mutant Myc-TBK1. *hTBK1* and *Ifnb1* mRNA levels were determined by qPCR. *n* = 3, biologically independent experiments. *P* values: *p* > 0.9999 (ns) for *hTBK1* TBK1 R47H and Q565P, *p* = 0.9941 (ns) for *hTBK1* TBK1 G217R, R228H, R357Q, and M559R, *p* < 0.0001 (****) for *Ifnb1* TBK1 WT, *p* > 0.9999 (ns) for *Ifnb1* TBK1 mutants. (**G**,** H**) Knockdown of *Ifnb1* abolished the reduction of the monomeric TDP-43 by TBK1-CM. A significant reduction in *Ifnb1* expression in CM-donor cells was confirmed by qPCR (**G**). *P* values: *p* < 0.0001 (****), *p* = 0.1031 (ns) for *hTBK1* siIfnb1-#1, 0.4162 (ns) for *hTBK1* siIfnb1-#2. Cells treated with the indicated CM were analyzed by immunoblotting. Representative immunoblotting images are shown alongside a quantification of the relative levels of FLAG and p-TDP proteins (**H**). *n* = 4, biologically independent experiments. *P* values: *p* = 0.0447 (*) for FLAG, 0.0377 (*) for p-TDP. (**I**) Pan-JAK inhibition abolished the reduction of the monomeric TDP-43 by TBK1-CM. Neuro2a cells transiently transfected with TDP-43^6M^-3×FLAG were treated with either Mock- or TBK1-CM in the presence of either DMSO or 5 μM tofacitinib. Representative immunoblotting images with a quantification of FLAG fold changes are shown. *n* = 3, biologically independent experiments. *P* values: *p* = 0.0059 (**), 0.3971 (ns). (**J**) Knockdown of *Ifnb1* exacerbates TDP-43 pathology driven by monomeric TDP-43 in cells. Neuro2a cells were co-transfected with the indicated plasmids and either control siRNA or siRNA against *Ifnb1*, cultured in differentiation medium, and subsequently analyzed by immunoblotting. Representative immunoblotting images with quantified FLAG fold changes and relative p-TDP levels are shown. *n* = 7, biologically independent experiments. *P* values: *p* = 0.0158 (*) for FLAG fold change, *p* = 0.5196 (ns), 0.0237 (*), 0.0001 (***) for p-TDP. All data were expressed as means ± SEM. Unpaired *t*-test with Welch’s correction [**D**, **I**, **J** (left graph)] and one-way ANOVA followed by Holm–Sidak’s multiple comparisons test [**B**, **C**, **E**–**H**, **J** (right graph)]. ns, not significant. Source data are available online for this figure.

In innate immunity, TBK1 mediates cytokine secretion by phosphorylating STING and IRF3 (Shu et al, 2014). To test whether the TBK1-linked humoral factor-mediated reduction of monomeric TDP-43 depends on this innate immune pathway, we pretreated cells with siRNAs targeting *Sting* or *Irf3* prior to TBK1 overexpression, and then performed the experiment using conditioned medium (CM) (Fig. 3A). Quantitative PCR (qPCR) analysis of the CM-donor cells confirmed efficient knockdown of both *Sting* and *Irf3*, and showed that these treatments did not affect the expression level of exogenous human *TBK1* (Fig. 3B). Immunoblotting of the CM-recipient cells revealed that knockdown of either *Sting* or *Irf3* abolished the reduction of TDP-43^6M^ and p-TDP levels by TBK1-CM (Fig. 3C). We observed strong induction of *Ifnb1* (IFNβ) expression upon TBK1 overexpression (Fig. 3D), which was significantly suppressed by *Sting* or *Irf3* knockdown (Fig. 3E) and was not induced by ALS/FTD-linked kinase-dead mutants (Fig. 3F); we therefore focused on IFNβ as the key humoral factor. By using two distinct siRNAs targeting *Ifnb1*, we confirmed inhibition of TBK1-induced *Ifnb1* expression (Fig. 3G). Consistently, immunoblotting of the CM-recipient cells revealed that *Ifnb1* knockdown in CM-donor cells completely abolished the effect of TBK1-CM on TDP-43^6M^ and p-TDP levels, demonstrating that IFNβ is the key cytokine responsible for TBK1-mediated clearance of TDP-43^6M^ (Fig. 3H). We also confirmed that inhibition of the JAK/STAT pathway, a canonical downstream signaling pathway of IFNβ, with the pan-JAK inhibitor tofacitinib completely abolished the reduction of TDP-43^6M^ by TBK1-CM (Fig. 3I). Importantly, knockdown of *Ifnb1* exacerbated accumulation of TDP-43 and p-TDP, particularly in cells overexpressing TDP-43^6M^ (Fig. 3J), suggesting that the increased TDP-43 pathology observed upon endogenous TBK1 suppression in Fig. 1H–K results from impaired IFNβ signaling. Collectively, these findings demonstrate that the TBK1-STING-IRF3-IFNβ-JAK-STAT axis is crucial for the clearance of monomeric TDP-43.

### IFNβ treatment reduces monomeric TDP-43 in neurons

**Figure 4:**
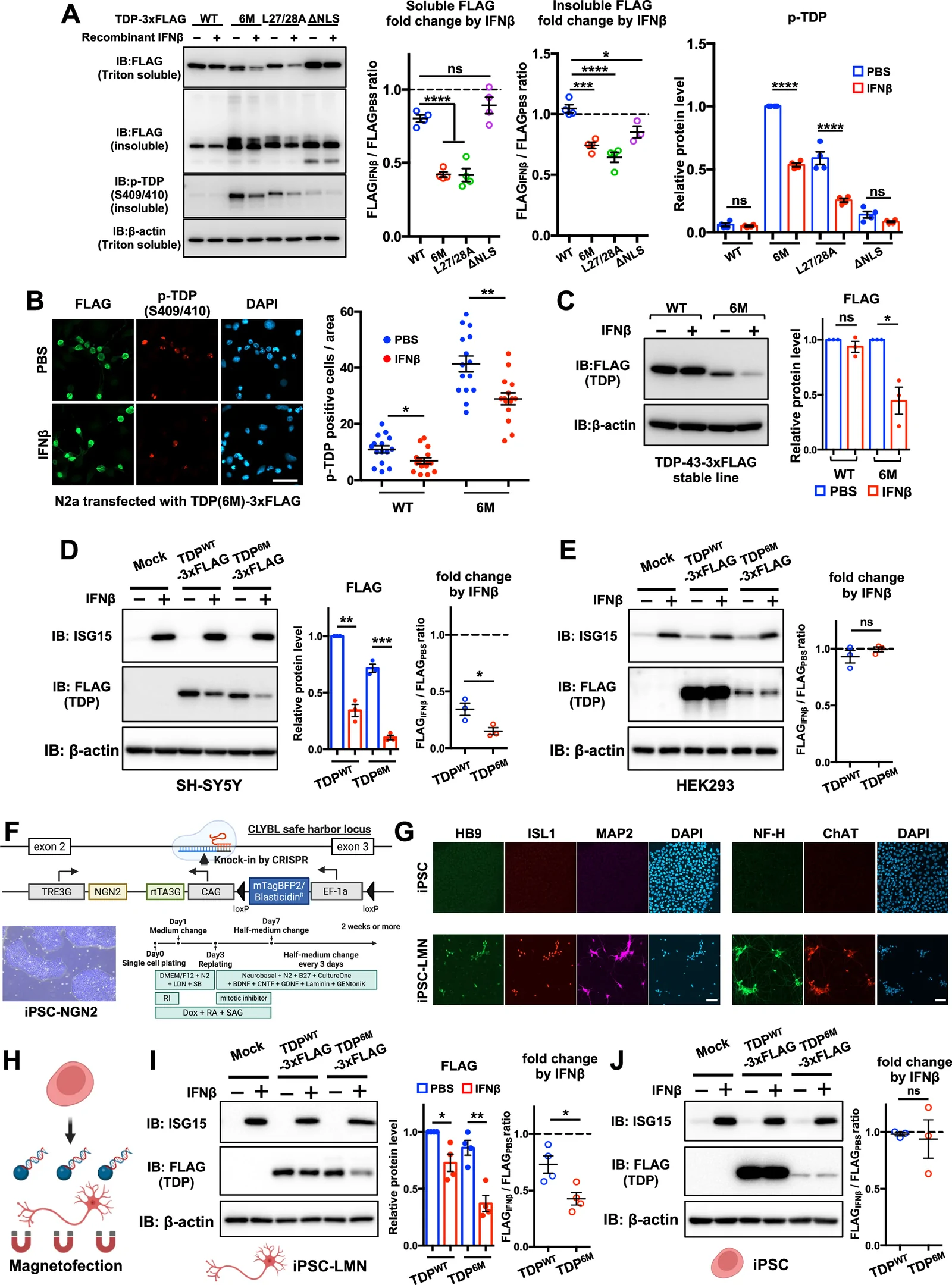
IFNβ treatment reduces monomeric TDP-43 in neurons. (**A**,** B**). Recombinant IFNβ treatment reduced monomeric TDP-43 mutants and suppressed p-TDP accumulation. Neuro2a cells transiently transfected with expression vectors of the indicated TDP-43 mutants were treated with either PBS or 10 ng/mL mouse IFNβ for 42 h. The cells were then fractionated into soluble and insoluble fractions using 1% Triton X-100 and analyzed by immunoblotting. Representative immunoblotting images with quantified FLAG fold changes and relative p-TDP levels are shown (**A**). *n* = 4, biologically independent experiments. *P* values: *p* < 0.0001 (****) for 6M and L27/28A Soluble FLAG fold change, *p* = 0.2436 (ns) for ΔNLS Soluble FLAG fold change, *p* = 0.0004 (***) for 6M Insoluble FLAG fold change, *p* <  0.0001 (****) for L27/28A Insoluble FLAG fold change, *p* = 0.0133 (*) for ΔNLS Insoluble FLAG fold change, *p* = 0.7537 (ns) for WT p-TDP, *p* < 0.0001 (****) for 6M and L27/28A p-TDP, *p* = 0.3396 (ns) for ΔNLS p-TDP. Reduction of p-TDP-positive cells by recombinant IFNβ was confirmed by immunocytochemistry (**B**). Representative images of the TDP-43^6M^ cells treated with PBS or IFNβ are shown. The number of p-TDP-positive cells　per 0.5 mm^2^ area is plotted, based on 15 areas from three independent biological replicates. *P* values: *p* = 0.0225 (*), 0.0017 (**). Scale bar, 50 μm. (**C**) Recombinant IFNβ selectively reduced the monomeric TDP-43 mutant in stable lines. Neuro2a cells stably expressing Dox-inducible TDP-43^WT^ or TDP-43^6M^-3×FLAG were treated with 1.5 μg/mL Dox in the presence of either PBS or 10 ng/mL mouse IFNβ for 48 h and analyzed by immunoblotting. Representative immunoblotting images with relative FLAG levels are shown. *n* = 3, biologically independent experiments. *P* values: *p* = 0.3219 (ns), 0.0455 (*). (**D**,** E**) Recombinant IFNβ treatment reduced TDP-43 in SH-SY5Y cells but not in HEK293 cells. SH-SY5Y cells (**D**) and HEK293 cells (**E**) were transiently transfected with the indicated expression vectors, treated with either PBS or 10 ng/mL human IFNβ for 48 h, and analyzed by immunoblotting. Representative immunoblotting images with quantified FLAG fold changes and relative FLAG levels are shown. *n* = 3, biologically independent experiments. *P* values: *p* = 0.0066 (**), 0.0005 (***), 0.0479 (*) for SH-SY5Y, *p* = 0.359 (ns) for HEK293. (**F**,** G**). Establishment of a human induced pluripotent stem cell (iPSC)-derived lower motor neuron (LMN) model. Schematic for the LMN induction protocol using a stable iPSC-NGN2 line and small molecules (**F**). A Tet-On-inducible NGN2 expression cassette was inserted into the CLYBL safe harbor locus in an iPSC line from a healthy donor (KOLF2.1J) by CRISPR-mediated genome editing. The phase-contrast microscopic image shows the isolated monoclonal iPSC-NGN2 line used in this study. Representative immunofluorescence images of undifferentiated iPSC-NGN2 cells and 2-week-old iPSC-NGN2-derived LMNs are shown in (**G**). MAP2 and neurofilament heavy chain (NF-H) were used as pan-neuron markers, and HB9, ISL1, and ChAT were used as lower motor neuron markers. Scale bars, 50 μm. (**H**–**J**). Recombinant IFNβ treatment reduced monomeric TDP-43 in iPSC-LMNs but not in undifferentiated iPSCs. iPSC-LMNs (**I**) and undifferentiated iPSC-NGN2 cells (**J**) were transfected with the indicated expression vectors, treated with IFNβ for 48 h, and analyzed by immunoblotting. Magnetofection was performed to transfect iPSC-LMNs (**H**). Representative immunoblotting data with quantified FLAG levels and the ratio of FLAG levels in IFNβ-treated cells relative to those in PBS-treated cells are shown (**I**, **J**). *n* = 4 (**I**) or *n* = 3 (**J**), biologically independent experiments. *P* values: *p* = 0.0386 (*) for WT FLAG, 0.0021 (**) for 6M FLAG, 0.0209 (*) for iPSC-LMN fold change, *p* = 0.8239 (ns) for iPSC fold change. All data were expressed as means ± SEM. Unpaired *t*-test with Welch’s correction (**B**–**E**, **I**, **J**) and one-way ANOVA followed by Holm–Sidak’s multiple comparisons test (**A**). ns, not significant. Source data are available online for this figure.

Next, we tested whether IFNβ treatment alone is sufficient to reduce TDP-43 levels. We examined the effects of IFNβ on TDP-43^WT^ and its mutants, including the monomeric mutants 6M and L27/28A (Mompeán et al, 2017), a nuclear localization signal-deficient mutant (ΔNLS) (Winton et al, 2008), an aggregation-prone mutant C173/175S (Shodai et al, 2013), and FALS-associated mutants A315T and M337V. We found that IFNβ treatment significantly reduced both monomeric TDP-43 mutants (6M and L27/28A) in the 1% Triton X-100-soluble and -insoluble fractions, as well as p-TDP in the insoluble fraction, with a more pronounced effect on the monomeric mutants than on TDP-43^WT^ or TDP-43^ΔNLS^ (Fig. 4A; Appendix Fig. S7). IFNβ also reduced C173/175S, A315T, and M337V mutants; however, the magnitude of reduction by IFNβ in these mutants was similar to that observed for WT (Appendix Fig. S8). Immunocytochemistry further confirmed that recombinant IFNβ treatment decreased the number of p-TDP-positive cells (Fig. 4B). We also tested the effects of IFNβ on TDP-43 protein levels using stable cell lines expressing human TDP-43 and confirmed that IFNβ treatment reduced TDP-43^6M^ levels (Fig. 4C). Similar results were obtained in the human neuronal cell line SH-SY5Y (Fig. 4D), but not in HEK293 cells (Fig. 4E). Next, to validate IFNβ-dependent monomeric TDP-43 clearance in post-mitotic bona fide neurons, we established a human induced pluripotent stem cell (iPSC)-derived lower motor neuron (LMN) model based on previously published protocols (Fernandopulle et al, 2018; Limone et al, 2023) (Fig. 4F). We confirmed that our iPSC-LMNs express mature neuron markers, such as MAP2 and neurofilament heavy chain (NF-H), and canonical LMN markers, such as HB9, ISL1, and choline acetyltransferase (ChAT) (Fig. 4G). We transduced TDP-43 expression vectors into the differentiated neurons with magnetofection (Fig. 4H) and demonstrated that IFNβ reduces monomeric TDP-43 in iPSC-LMNs (Fig. 4I), but not in undifferentiated iPSCs (Fig. 4J). Collectively, these results demonstrate that IFNβ reduces monomeric TDP-43, preferentially in neurons, suggesting that this regulatory mechanism may be specific to neurons.

### IFNβ receptor is downregulated in the spinal motor neurons of patients with ALS

**Figure 5:**
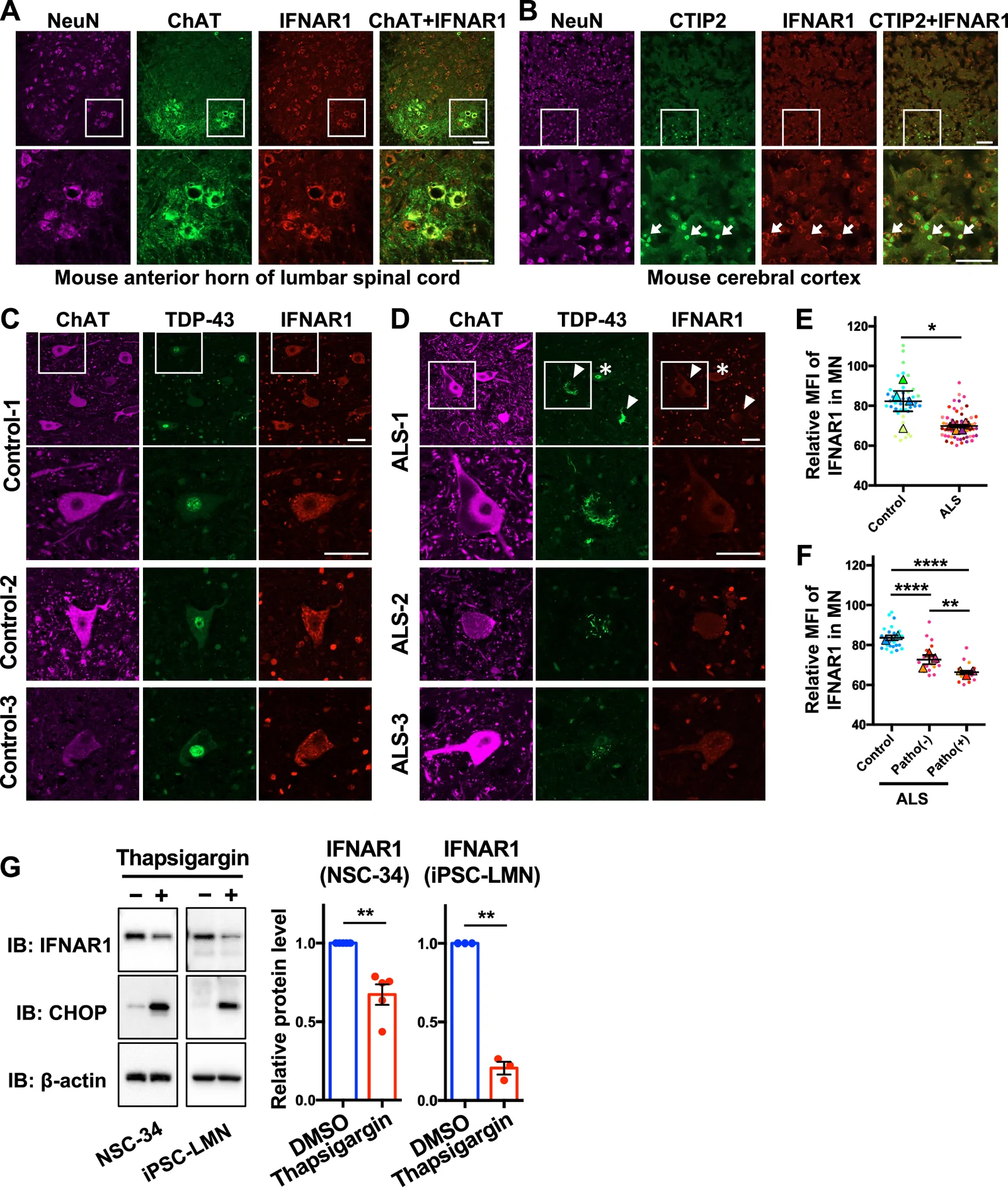
The level of IFNAR1, a subunit of the receptor for IFNβ, is reduced in the spinal motor neurons of patients with ALS. (**A**,** B**) IFNAR1 is expressed in ALS/FTD-susceptible neurons in mice. Representative images show IFNAR1 immunostaining with a pan-neuron marker NeuN and either a spinal motor neuron marker ChAT or a layer V cortical neuron marker CTIP2 in the lumbar spinal cords (**A**) or cerebral cortices (**B**) of WT mice. Representative confocal images from two independent experiments are shown. Magnified images of the areas indicated by the white squares are shown below. The arrows indicate CTIP2- and IFNAR1-positive cells. Scale bars, 100 μm. (**C**–**F**) IFNAR1 is downregulated in the spinal motor neurons of patients with ALS. Representative images show IFNAR1 immunostaining with ChAT and TDP-43 in the spinal cords of control subjects (**C**) and ALS patients (**D**). The asterisk and arrowheads indicate TDP-43 pathology-negative and pathology-positive motor neurons, respectively. Scale bar, 50 μm. Magnified images of the areas indicated by the white squares are shown below (**C**: Control-1, **D**: ALS-1). Relative mean fluorescence intensities (MFI) of IFNAR1 in a single motor neuron are plotted in a SuperPlots-style format (Lord et al, 2020) (**E**, **F**). Forty-four motor neurons from 4 control subjects and 63 from 6 ALS patients were quantified. In (**F**), the results for motor neurons from 2 controls (Control-1 and -2) and 3 ALS patients (ALS-1, -2, and -3) are shown, in which motor neurons from ALS patients are subdivided into two groups based on the presence or absence of TDP-43 pathology. MN, motor neuron. Patho (−), TDP-43 pathology-negative. Patho (+), TDP-43 pathology-positive. *P* values: *p* = 0.0165 (*), 0.0068 (**), *p* < 0.0001 (****). Statistical significance between the non-ALS and ALS groups (**E**) was assessed using a linear mixed-effects model with diagnosis as a fixed effect and donor as a random effect. In (**F**), additional comparisons among non-ALS, TDP-43 pathology-negative ALS, and TDP-43 pathology-positive ALS motor neurons were performed using a linear mixed-effects model with group as a fixed effect and donor as a random effect, followed by post hoc pairwise comparisons with Tukey’s adjustment. Given the limited number of donors included in the analysis shown in (**F**) (two control and three ALS donors), estimation of the donor-level random effect may be unstable, and the results should therefore be interpreted with caution.　*P* values for the fixed effects were calculated using Satterthwaite’s approximation for degrees of freedom. (**G**) ER stress reduces IFNAR1 protein levels. NSC-34 cells and iPSC-LMNs at 2 weeks were treated with 1 μM thapsigargin for 12 h (NSC-34) or 10 μM for 48 h (iPSC-LMN). CHOP was assessed to confirm the induction of ER stress. *n* = 5 (NSC-34) or 3 (iPSC-LMNs), biologically independent experiments. *P* values: *p* = 0.0072 (**) for NSC-34, 0.0026 (**) for iPSC-LMN. All data were expressed as means ± SEM. Unpaired *t*-test with Welch’s correction (**G**). Source data are available online for this figure.

IFNβ binds to the interferon α/β receptor (IFNAR), a heterodimer composed of IFNAR1 and IFNAR2, to activate downstream signaling (Ivashkiv and Donlin, 2014). Although IFNAR is ubiquitously expressed, as demonstrated by studies using systemic IFNAR1-null mice (Müller et al, 1994), the integrated single-cell/nucleus RNA-sequencing data from various tissues in the Human Protein Atlas (http://www.proteinatlas.org/celltype) (Karlsson et al, 2021) (Appendix Fig. S9A), and single-nucleus RNA-sequencing from human spinal cord (Yadav et al, 2023) (Appendix Fig. S9B), the detailed protein expression pattern of IFNAR in the spinal cord and brain has not been fully characterized. Therefore, we investigated whether neurons susceptible to ALS/FTD express the IFNβ receptor by immunostaining for IFNAR1, one of the two subunits of the receptor. Immunofluorescence analysis of IFNAR1 in the lumbar spinal cord and cerebral cortex of wild-type mice revealed that IFNAR1 is expressed in NeuN-positive neurons, including ChAT-positive spinal motor neurons and COUP-TF-interacting protein 2 (CTIP2)-positive layer V cortical neurons (Fig. 5A,B), and also in IBA1-positive microglia (Appendix Fig. S10). These findings suggest that type I IFN signaling plays important roles in neurons, possibly including IFNβ-mediated clearance of aggregation-prone proteins. Next, we investigated whether IFNAR1 protein expression is altered in motor neurons of sporadic ALS patients with TDP-43 pathology. In the controls, strong IFNAR1 signals were observed in motor neurons (Fig. 5C), consistent with the results obtained in mice. However, IFNAR1 fluorescence intensity in motor neurons from ALS patients (Fig. 5D) was significantly lower than that in the controls (Fig. 5E). Furthermore, we compared IFNAR1 fluorescence intensity between TDP-43 pathology-negative and -positive motor neurons within ALS patients and found that IFNAR1 expression was lower in pathology-positive neurons (Fig. 5F; Appendix Fig. S11). These results imply that deregulated IFNβ signaling in motor neurons may be associated with TDP-43 pathology in ALS. To elucidate the mechanism underlying IFNAR1 reduction, we examined whether ER stress affects IFNAR1 expression and found that thapsigargin-induced ER stress reduced IFNAR1 levels in NSC-34 cells and iPSC-LMNs (Fig. 5G), suggesting that the reduction of IFNAR1 in ALS motor neurons may be attributable to ER stress (Zhao et al, 2022).

### IFNβ promotes degradation of monomeric TDP-43 through the immunoproteasome pathway

**Figure 6:**
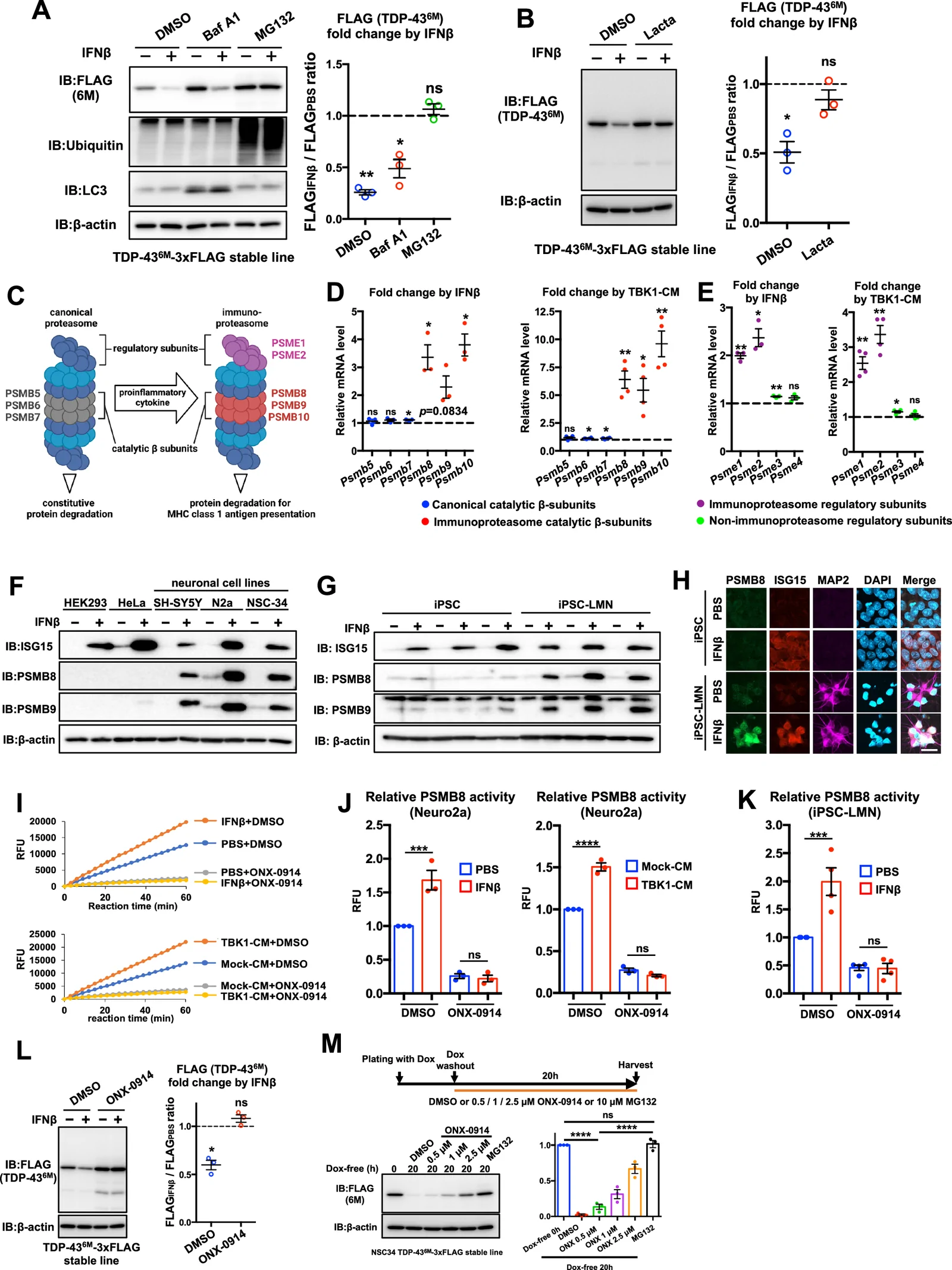
IFNβ upregulates immunoproteasome and promotes degradation of monomeric TDP-43. (**A**,** B**). Proteasome inhibitors abolish the reduction of TDP-43^6M^ by IFNβ treatment. NSC-34 cells stably expressing Dox-inducible TDP-43^6M^-3×FLAG were treated with 1.5 μg/mL Dox and either PBS or 10 ng/mL mouse IFNβ for 24 h. The cells were additionally treated with DMSO, 100 nM bafilomycin A1 (Baf A1), or 10 μM MG132 (**A**), or with DMSO or 2.5 μM lactacystin (**B**). Representative immunoblotting images with quantified fold changes of FLAG are shown. *n* = 3, biologically independent experiments. *P* values: *p* = 0.0012 (**), 0.0284 (*), 0.3349 (ns) for (**A**), *p* = 0.023 (*), 0.253 (ns) for (**B**). (**C**) A schematic illustration of the canonical proteasome and immunoproteasome. (**D**–**F**) IFNβ induces expression of immunoproteasome subunits in neuronal cells. In (**D**) and (**E**), Neuro2a cells were treated with either PBS or 10 ng/mL mouse IFNβ, or either Mock- or TBK1-CM for 24 h, and the expression levels of the indicated proteasome catalytic β-subunits (**D**) and regulatory subunits (**E**) were determined by qPCR. Relative mRNA levels are plotted. *n* = 3 or 4, biologically independent experiments. *P* values: *p* = 0.4305 (ns) for IFNβ *Psmb5*, 0.1491 (ns) for IFNβ *Psmb6*, 0.0123 (*) for IFNβ *Psmb7*, 0.0355 (*) for IFNβ *Psmb8*, 0.0196 (*) for IFNβ *Psmb10*, 0.1266 (ns) for TBK1-CM *Psmb5*, 0.0252 (*) for TBK1-CM *Psmb6*, 0.0412 (*) for TBK1-CM *Psmb7*, 0.0061 (**) for TBK1-CM *Psmb8*, 0.0235 (*) for TBK1-CM *Psmb9*, 0.0051 (**) for TBK1-CM *Psmb10*, 0.0038 (**) for IFNβ *Psme1*, 0.0176 (*) for IFNβ *Psme2*, 0.0081 (**) for IFNβ *Psme3*, 0.1104 (ns) for IFNβ *Psme4*, 0.0034 (**) for TBK1-CM *Psme1*, 0.0028 (*) for TBK1-CM *Psme2*, 0.015 (*) for TBK1-CM *Psme3*, 0.3731 (ns) for TBK1-CM *Psme4*. In (**F**), the indicated cell lines were treated with either PBS or 10 ng/mL human or mouse IFNβ for 30 h and analyzed by immunoblotting to assess protein levels of immunoproteasome subunits, PSMB8 and PSMB9. ISG15 was assessed to confirm a general response to IFNβ. N2a, Neuro2a. Representative immunoblotting images from three biologically independent experiments are shown. (**G**,** H**). IFNβ induces expression of immunoproteasome subunits in iPSC-LMNs. Undifferentiated iPSCs and iPSC-LMNs at 4 weeks (**G**) or 2 weeks (**H**) were treated with either PBS or 10 ng/mL human IFNβ for 30 h and analyzed by immunoblotting (**G**) or immunocytochemistry (**H**). Representative confocal images from two independent experiments are shown. Scale bar, 20 μm. (**I**–**K**) IFNβ induced functional immunoproteasome. Neuro2a cells and iPSC-LMNs at 2 weeks were treated with either PBS or 10 ng/mL mouse or human IFNβ, or either Mock- or TBK1-CM for 24 h and analyzed by immunoproteasome activity assay. Representative kinetic assay results (**I**) and relative fluorescence unit (RFU) at the endpoints of the assays (**J**, **K**) are shown. *n* = 3 or 4, biologically independent experiments. *P* values: *p* = 0.0005 (***) for Neuro2a IFNβ DMSO, 0.7535 (ns) for Neuro2a IFNβ ONX-0914, 0.1421 (ns) for Neuro2a TBK1-CM ONX-0914, 0.0008 (***) for iPSC-LMN DMSO, 0.9472 (ns) for iPSC-LMN ONX-0914, *p* < 0.0001 (****) for Neuro2a TBK1-CM DMSO. The samples treated with ONX-0914, a selective immunoproteasome inhibitor, were prepared as negative controls. (**L**) Immunoproteasome-specific inhibition by ONX-0914 abolishes the reduction of TDP-43^6M^ by IFNβ. NSC-34 cells stably expressing Dox-inducible TDP-43^6M^-3×FLAG were treated with Dox and either PBS or 10 ng/mL mouse IFNβ for 24 h under DMSO or 0.5 µM ONX-0914 treatment. Representative immunoblotting images with relative fold changes of FLAG are shown. *n* = 3, biologically independent experiments. *P* values: *p* = 0.0151 (*), 0.1740 (ns). (**M**) A concentration of 0.5 μM ONX-0914 has a minimal impact on canonical proteasome activity. NSC-34 cells stably expressing doxycycline (Dox)-inducible TDP-43^6M^-3×FLAG were treated as indicated and analyzed by immunoblotting. Representative immunoblotting images are shown alongside a quantification of the relative levels of FLAG. *n* = 3, biologically independent experiments. *P* values: *p* = 0.812 (ns), *p* < 0.0001 (****). All data were expressed as means ± SEM. Unpaired *t*-test with Welch’s correction (**A**, **B**, **D**, **E**, **L**) and one-way ANOVA followed by Holm–Sidak’s multiple comparisons test (**J**, **K**, **M**). ns, not significant. Source data are available online for this figure.

We then explored the downstream mechanisms of IFNβ. Based on our findings that monomeric TDP-43 reduction mediated by TBK1 was blocked by MG132 treatment (Appendix Fig. S5) and that IFNβ reduced the mild detergent-soluble form of monomeric TDP-43 mutants (Fig. 4A), which is typically degraded via the proteasome, we focused on the ubiquitin–proteasome pathway. First, we found that treatment with canonical proteasome inhibitors, MG132 or lactacystin, completely abolished the IFNβ-induced reduction of TDP-43^6M^ (Fig. 6A,B). Given this proteasome dependency, we examined whether the immunoproteasome, a subtype of the proteasome, contributes to the clearance of aggregation-prone monomeric TDP-43. The immunoproteasome is a specialized form of the proteasome complex that is normally induced by proinflammatory cytokines, such as TNF and IFNγ. Its overall structure is similar to that of the canonical proteasome; however, it incorporates two immunoproteasome-specific regulatory subunits, PSME1 and PSME2 (also known as PA28α and PA28β, respectively), and three catalytic β-subunits, PSMB8, PSMB9, and PSMB10, thereby facilitating the generation of peptides for MHC class I antigen presentation (McCarthy and Weinberg, 2015) (Fig. 6C). Although IFNβ is not typically considered a canonical inducer of the immunoproteasome, previous studies have shown that type I IFNs can also induce immunoproteasome subunits in hepatocytes (Shin et al, 2006), epithelial cells, and fibroblasts (Wang et al, 2023). Therefore, we tested whether IFNβ upregulates immunoproteasome subunits in neurons. qPCR analysis of Neuro2a cells revealed that both IFNβ and TBK1-CM induced the expression of catalytic β-subunits and regulatory subunits of the immunoproteasome (Fig. 6D,E). Intriguingly, we found that IFNβ robustly induced PSMB8 and PSMB9 at the protein level in three neuronal cell lines (SH-SY5Y, Neuro2a, and NSC-34 cells), mouse primary spinal neurons, and iPSC-LMNs, but not, or only faintly, in HEK293 cells, HeLa cells, and undifferentiated iPSCs (Fig. 6F–H; Appendix Fig. S12). Next, we assessed immunoproteasome activity using a PSMB8-specific fluorogenic substrate, Ac-Ala-Asn-Trp-7-amino-4-methylcoumarin (Ac-ANW-AMC). As expected, the relative immunoproteasome activity was increased by IFNβ or TBK1-CM, and was not increased in the presence of the PSMB8-specific inhibitor ONX-0914 in Neuro2a cells and iPSC-LMNs (Fig. 6I–K). Finally, we concluded that the reduction of monomeric TDP-43 by IFNβ depends on the immunoproteasome pathway, because treatment with the PSMB8-specific inhibitor ONX-0914 completely abolished the IFNβ-induced reduction of monomeric TDP-43 (Fig. 6L). We confirmed that the ONX-0914 concentration used in Fig. 6L had minimal effects on the canonical proteasome (Fig. 6M).

### Heterozygous deletion of *Tbk1* in an ALS model mouse impairs immunoproteasome induction and exacerbates polyubiquitinated protein accumulation at disease onset

**Figure 7:**
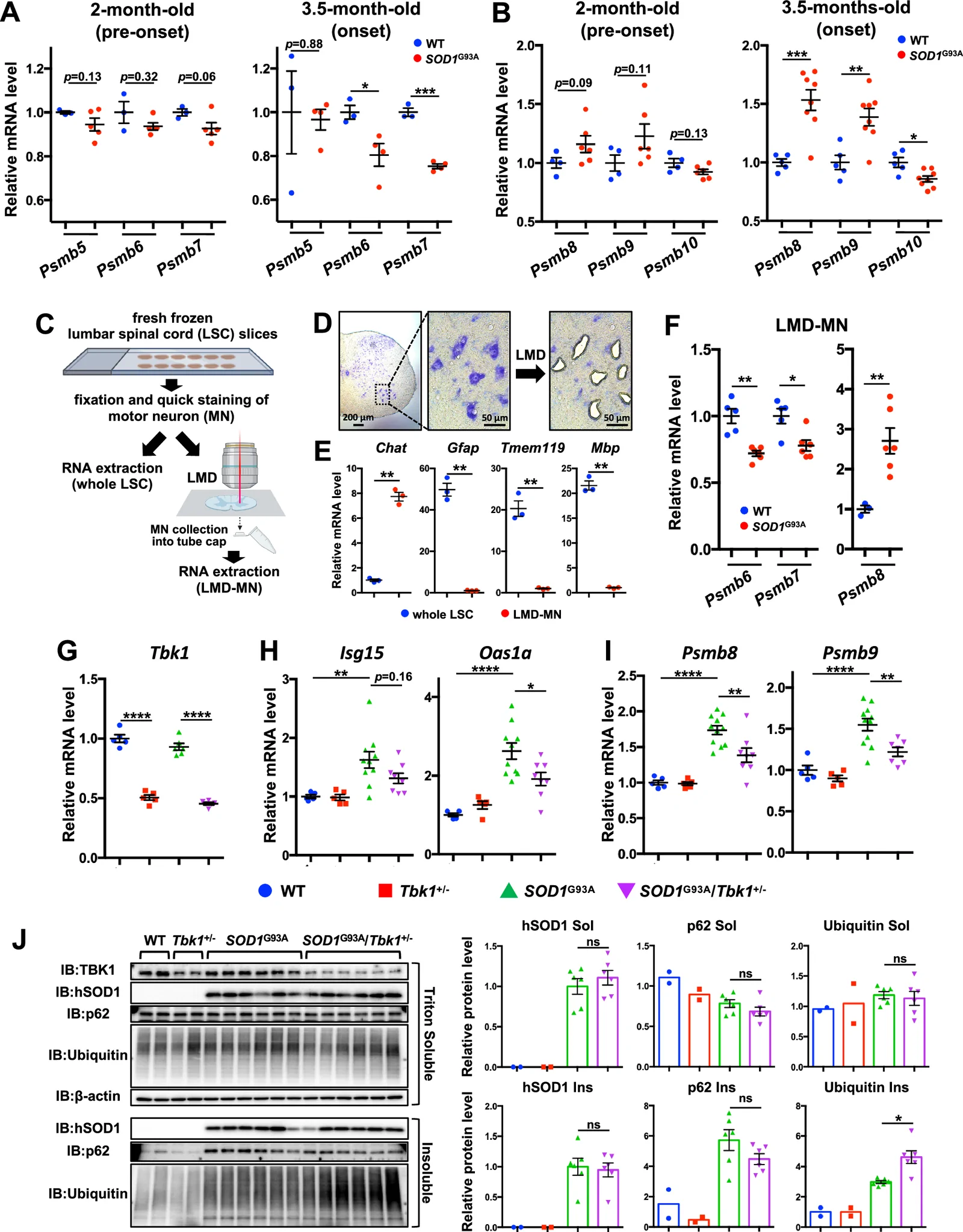
Heterozygous deletion of *Tbk1* in *SOD1*^G93A^ mice causes impaired immunoproteasome induction and exacerbates polyubiquitinated protein accumulation. (**A**) Canonical proteasome catalytic β-subunits, *Psmb6* and *Psmb7*, were downregulated in the lumbar spinal cords of *SOD1*^G93A^ mice at the onset stage. Total RNA was extracted from the lumbar spinal cords of 2-month-old (pre-onset) or 3.5-month-old (onset) *SOD1*^G93A^ mice, as well as from age-matched wild-type (WT) mice. mRNA levels of the indicated genes relative to those of the WT were analyzed by qPCR and are plotted [2-month-old WT (*n* = 3), 2-month-old *SOD1*^G93A^ (*n* = 5), 3.5-month-old WT (*n* = 3), and 3.5-month-old *SOD1*^G93A^ (*n* = 4)]. *P* values: *p* = 0.025 (*), 0.001 (***). (**B**) Immunoproteasome β-subunits, *Psmb8* and *Psmb9*, were upregulated in the lumbar spinal cords of *SOD1*^G93A^ mice at the onset stage. Relative mRNA expression levels of the indicated genes were analyzed and plotted as in (**A**) [2-month-old WT (*n* = 4), 2-month-old *SOD1*^G93A^ (*n* = 6), 3.5-month-old WT (*n* = 5), and 3.5-month-old *SOD1*^G93A^ (*n* = 8)]. *P* values: *p* = 0.0004 (***), 0.002 (**), 0.023 (*). (**C**–**E**) Validation of laser microdissection (LMD)-based motor neuron isolation. Motor neurons were dissected from the lumbar spinal cords (LSCs) of WT mice by LMD and compared with whole LSCs (**C**). Representative images of toluidine blue-stained LSC sections before and after LMD are shown (**D**). mRNA levels of cell type-specific markers (*Chat*, for motor neurons; *Gfap*, for astrocytes; Tmem119, for microglia; *Mbp*, for oligodendrocytes) were analyzed by qPCR and plotted [whole LSC (*n* = 3), LMD-isolated motor neurons (LMD-MNs) (*n* = 3)] (**E**). *P* values: *p* = 0.0016 (**) for *Chat*, 0.004 (**) for *Gfap*, 0.0091 (**) for *Tmem119*, 0.0018 (**) for *Mbp*. (**F**) Canonical and immunoproteasome catalytic β-subunits were downregulated and upregulated, respectively, in motor neurons from the lumbar spinal cords of *SOD1*^G93A^ mice at disease onset. LMD-MNs were isolated from the lumbar spinal cords of 3.5-month-old *SOD1*^G93A^ mice and age-matched WT mice. Relative mRNA expression levels of the indicated genes were analyzed and plotted [WT (*n* = 3–5), *SOD1*^G93A^ (*n* = 6)]. *P* values: *p* = 0.0047 (**) for *Psmb6*, 0.0137 (*) for *Psmb7*, 0.0026 (**) for *Psmb8*. (**G**–**I**). Heterozygous knockout of *Tbk1* in *SOD1*^G93A^ mice impaired the induction of type I IFN response and immunoproteasome in the lumbar spinal cords at the onset stage. Total RNA was extracted from the lumbar spinal cords of 3.5-month-old mice with the indicated genotypes. mRNA levels of the indicated genes relative to those of the WT were analyzed by qPCR and are plotted [WT (*n* = 5), *Tbk1*^+/−^ (*n* = 5), *SOD1*^G93A^ (*n* = 6–11), *SOD1*^G93A^/*Tbk1*^+/−^ (*n* = 6–8)]. *P* values: *p* < 0.0001 (****), *p* = 0.0056 (**) for *Isg15*, *p* = 0.0138 (*) for *Oas1a*, *p* = 0.004 (**) for *Psmb8*, *p* = 0.0046 (**) for *Psmb9*. (**J**) Heterozygous deletion of *Tbk1* increased Triton X-100-insoluble, polyubiquitinated proteins in the lumbar spinal cords of *SOD1*^G93A^ mice. Total protein was extracted from the lumbar spinal cords of 3.5-month-old mice with the indicated genotypes, fractionated into 1% Triton X-100-soluble and -insoluble fractions, and analyzed by immunoblotting. Representative immunoblotting data and relative protein levels are shown [WT (*n* = 2), *Tbk1*^+/−^ (*n* = 2), *SOD1*^G93A^ (*n* = 6), *SOD1*^G93A^/*Tbk1*^+/−^ (*n* = 6)]. *P* values: *p* = 0.4363 (ns) for hSOD1 Sol, 0.7756 (ns) for hSOD1 Ins, 0.2069 (ns) for p62 Sol, 0.1465 (ns) for p62 Ins, 0.695 (ns) for Ubiquitin Sol, 0.0108 (*) for Ubiquitin Ins. All data were expressed as means ± SEM. Unpaired *t*-test with Welch’s correction (**A**, **B**, **E**, **F**, **J**) and one-way ANOVA followed by Holm–Sidak’s multiple comparisons test (**G**, **H**, **I**). ns, not significant. Source data are available online for this figure.

Lastly, we investigated the role of TBK1 in immunoproteasome induction in vivo using *SOD1*^G93A^ transgenic ALS model mice (Gurney et al, 1994). First, we found that, while the canonical proteasome β-subunits, *Psmb6* and *Psmb7*, were downregulated in the lumbar spinal cord of the ALS mice at disease onset (3.5 months old) (Fig. 7A), the immunoproteasome β-subunits, *Psmb8* and *Psmb9*, were upregulated (Fig. 7B). To investigate whether these molecular alterations occur in motor neurons, we performed laser microdissection (LMD) to isolate motor neurons from whole lumbar spinal cords (Fig. 7C,D). We validated this approach by confirming enrichment of *Chat*-positive motor neurons with minimal inclusion of glial cells (Fig. 7E). Consistently, LMD-isolated motor neurons also showed reductions in the canonical proteasome β-subunits and an increase in the immunoproteasome β-subunit *Psmb8* (Fig. 7F), suggesting that immunoproteasome induction in motor neurons may serve as a compensatory mechanism in response to increased proteostatic stress in ALS. Next, we investigated the role of TBK1 in immunoproteasome induction and ubiquitinated protein accumulation in vivo using heterozygous *Tbk1* knockout *SOD1*^G93A^ transgenic mice, which exhibit earlier disease onset compared to *SOD1*^G93A^ mice (Brenner et al, 2019). It is important to note that homozygous deletion of *Tbk1* in mice is embryonically lethal (Bonnard et al, 2000). Heterozygous deletion of *Tbk1*, confirmed by 50% reduction of *Tbk1* mRNA (Fig. 7G), in *SOD1*^G93A^ transgenic mice led to decreased type I IFN responses, as assessed by reduced expression of *Isg15* and *Oas1a* (Fig. 7H). Immunoproteasome induction in the ALS mice evaluated by the expression of *Psmb8* and *Psmb9* was also impaired by heterozygous *Tbk1* deletion (Fig. 7I). These results demonstrated that TBK1 plays an important role in the induction of type I interferon response and immunoproteasome at the disease onset of ALS. Although we were unable to detect IFNβ in mouse tissues, likely due to its extremely low expression levels, it is highly plausible that neurons are the primary source of IFNβ, as phosphorylation of STING at Ser365, a prerequisite for type I IFN induction, was observed in neurons, including ChAT-positive motor neurons, of ALS mice at the age of 2 and 3.5 months (Appendix Fig. S13). Finally, to investigate the relationship between impairment of the TBK1–immunoproteasome axis and abnormal protein accumulation, we analyzed 1% Triton X-100-soluble and -insoluble protein fractions extracted from the lumbar spinal cords of mice with each genotype by immunoblotting. While the levels of an autophagy marker protein, p62, did not differ between *SOD1*^G93A^ and *SOD1*^G93A^/*Tbk1*^+/−^ mice, *SOD1*^G93A^/*Tbk1*^+/−^ mice had higher levels of Triton-insoluble polyubiquitinated proteins, possibly due to impaired induction of the immunoproteasome at the onset stage (Fig. 7J). These results suggest that TBK1 deficiency exacerbates abnormal protein accumulation and aggregation, possibly through impairment of the immunoproteasome pathway in vivo.

## Discussion

**Figure 8:**
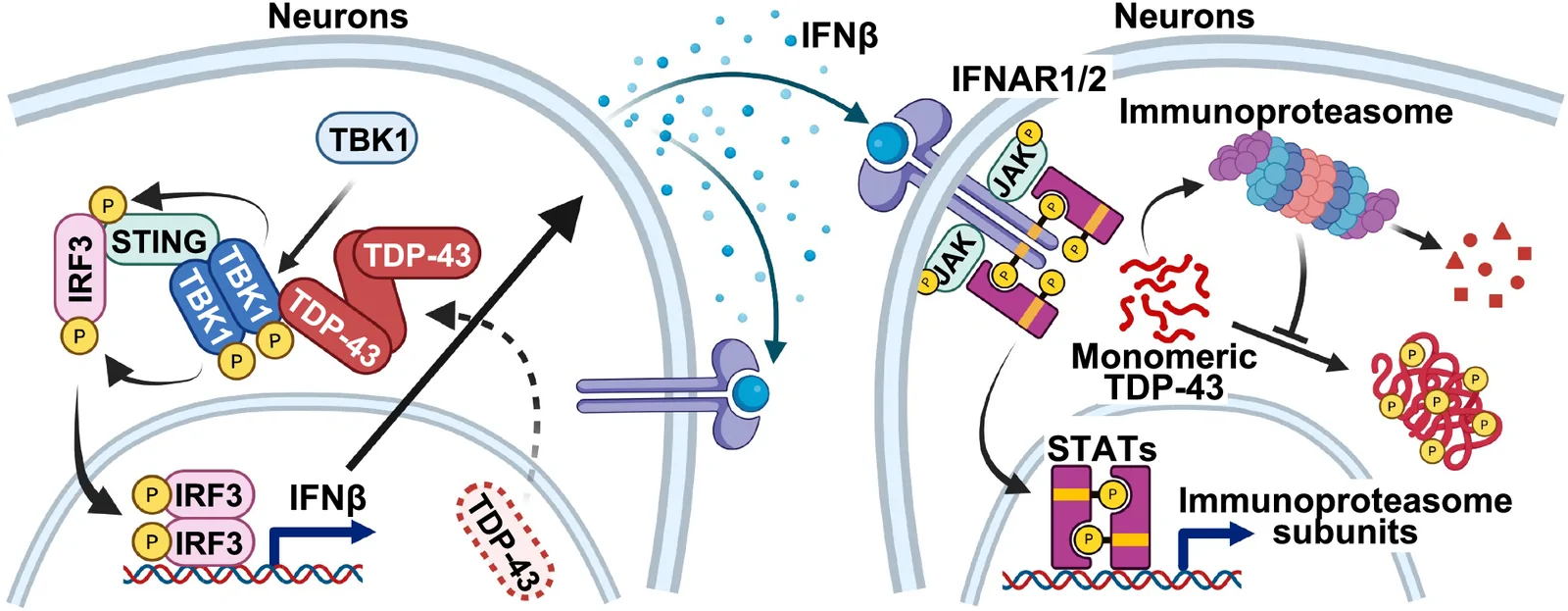
A schematic summary of the TBK1-IFNβ-immunoproteasome axis in TDP-43 clearance. TBK1 is activated by various cellular stresses, including proteostatic stress (Watanabe et al, 2023) and mitochondrial DNA (mtDNA) release (Yu et al, 2020). The present study also revealed that TBK1 is activated by cytoplasmic monomeric TDP-43 via a physical association. Upon activation, phospho-TBK1 phosphorylates STING and IRF3, which leads to IFNβ induction. IFNβ subsequently upregulates the expression of immunoproteasome subunits in neurons, thereby promoting the degradation of aggregation-prone monomeric TDP-43. In ALS/FTD patients, TBK1 activity (phospho-TBK1) (Shao et al, 2022; Watanabe et al, 2023) and IFNAR1 are impaired (present study).

While accumulating evidence has suggested that TBK1 is a key molecule in ALS/FTD, the mechanism through which TBK1 deregulation leads to TDP-43 pathology remains poorly understood. In this study, we demonstrated that TBK1 is activated by aggregation-prone monomeric TDP-43 via a physical association (Fig. 1) and that it induces the immunoproteasome via IFNβ signaling, thereby promoting the degradation of aggregation-prone monomeric TDP-43 in neurons, including human iPSC-derived lower motor neurons (Fig. 8). Importantly, we found that the IFNβ receptor was downregulated in the spinal motor neurons of ALS patients, particularly in those with TDP-43 pathology. We also observed immunoproteasome induction in spinal motor neurons of *SOD1*^G93A^ ALS model mice at disease onset. Notably, heterozygous deletion of *Tbk1* in the ALS mice resulted in impaired immunoproteasome induction and increased polyubiquitinated protein accumulation. Based on these findings, we propose that the TBK1-IFNβ-immunoproteasome axis represents an inducible and protective mechanism that mitigates proteostatic stress, such as TDP-43 pathology, in neurons. Our results suggest that impairment of this pathway, due to deregulated TBK1 or reduced IFNAR1 expression, may contribute to the development of TDP-43 pathology in ALS/FTD.

Our findings broaden the current understanding of TBK1 function beyond its established roles in autophagy, the endosomal pathway, and inflammation. We demonstrated that TBK1 activates the immunoproteasome pathway through IFNβ signaling, thereby enhancing the clearance of misfolded proteins. Although the immunoproteasome has been primarily known as a component of adaptive immunity responsible for MHC class I antigen presentation, our results reveal a critical role of the immunoproteasome in neuronal protein quality control, particularly in the context of neurodegenerative diseases. This function is particularly important for the clearance of defective ribosomal products (DRIPs), which are aberrant, misfolded, or incomplete polypeptides that account for approximately 30% of newly synthesized proteins under physiological conditions (Seifert et al, 2010; Schubert et al, 2000). The aggregation-prone monomeric TDP-43 mutant, TDP-43^6M^, exemplifies such a substrate: it is unable to form a physiological polymeric structure (Afroz et al, 2017), is extremely unstable (Pérez-Berlanga et al, 2023), and lacks its splicing function (Oiwa et al, 2023), all of which are characteristics consistent with a misfolded protein. Thus, it is reasonable to assume that misfolded proteins associated with neurodegeneration, such as monomeric TDP-43, are preferentially degraded via the immunoproteasome pathway.

The TBK1-IFNβ-immunoproteasome pathway offers two distinct advantages over the autophagy/endosomal–lysosome pathways. First, this pathway depends on the proteasome for the degradation of substrates. Misfolded proteins, including misfolded TDP-43, are major substrates of the proteasome, and when not properly eliminated, they can serve as precursors to protein aggregates. Therefore, the proteasome is considered the upstream degradation machinery required to maintain proteostasis, and maintaining or enhancing its activity may represent a more effective strategy for preventing protein aggregate formation, rather than degrading aggregates by the lysosome after they have formed. Intriguingly, a previous study using mouse models demonstrated that disruption of the proteasome, but not autophagy, recapitulated TDP-43 pathology in motor neurons (Tashiro et al, 2012), highlighting the importance of the proteasome pathway in TDP-43 proteostasis. Second, immunoproteasome induction can occur in a non-cell-autonomous manner via IFNβ. This feature enables simultaneous regulation of proteostasis across multiple cells, even when TBK1 is inactive in some adjacent cells. In the frontal cortices of c9FTD patients, phosphorylated TBK1 has been reported to be sequestered into dipeptide repeat protein aggregates, thereby limiting its activity (Shao et al, 2022). Under such conditions, active TBK1 derived from neighboring cells may support affected cells in mitigating proteostatic stress through IFNβ signaling. This non-cell-autonomous mechanism may also complement the canonical cell-intrinsic unfolded protein response (UPR), extending proteostatic support to the tissue microenvironment when cell-intrinsic capacity is exceeded. However, as direct evidence for this model remains limited, further studies are needed to clarify the relationship between these two systems.

Previous studies using mouse models have shown that a single genetic manipulation of *Tbk1*, such as knockout or knockin of ALS/FTD-linked mutations, is insufficient to recapitulate TDP-43 pathology (Brenner et al, 2019; Gerbino et al, 2020; Brenner et al, 2024). This discrepancy between human and mouse pathology may be attributable to the limitations of mouse models. Indeed, an embryonic stem cell-derived human motor neuron model demonstrated that loss of TBK1 activity is sufficient to cause early-stage motor neuron degenerative phenotypes and mild TDP-43 pathology (Hao et al, 2021). In mouse models, TBK1 appears to affect ALS/FTD and TDP-43 pathology as a so-called “second hit”. Sieverding and colleagues showed that heterozygous deletion of *Tbk1* in *TDP-43*^G298S^ transgenic mice worsened neuromuscular junction pathology, although TDP-43 pathology remained unaltered (Sieverding et al, 2021). Another study using a *C9orf72* mouse model of c9FTD-ALS demonstrated that introducing the disease-linked R228H mutation in *Tbk1* exacerbated phenotypes, neurodegeneration, and p-TDP accumulation (Shao et al, 2022). In the present study, we found that heterozygous deletion of *Tbk1* did not alter immunoproteasome subunit expression or protein accumulation in wild-type mice but did so in *SOD1*^G93A^ mice (Fig. 7). Consistent with this notion, heterozygous *Tbk1* deletion in *SOD1*^G93A^ mice increased ubiquitinated protein accumulation without altering p62 levels (Fig. 7J), suggesting a stage-dependent shift between autophagy and immunoproteasome pathways. Importantly, we also demonstrated that TBK1 is activated in response to monomeric TDP-43 burden and suppresses TDP-43 accumulation. Thus, TBK1 appears to play important roles, particularly under disease conditions.

In the present study, we unexpectedly found that lysosomal inhibition did not affect TBK1-mediated clearance of aggregation-prone TDP-43. One possible explanation is that the induced immunoproteasome pathway may act upstream of the lysosomal pathway, and that enhanced proteasome activity alone is sufficient to prevent TDP-43 accumulation, at least under these experimental conditions. It is widely accepted that both the ubiquitin–proteasome system and the autophagy–endosome–lysosome pathway are essential for proteostasis, and the relative requirement for each degradation pathway likely depends on various factors, including cell types, cellular conditions, and disease stage.

Although proinflammatory cytokines such as TNF, interleukin-1β, and IFNγ exacerbate neurodegeneration through glial overactivation, the role of type I IFNs remains controversial and appears to be context-dependent, varying by cell type, disease stage, and disease type. In a Parkinson’s disease (PD) model, Ejlerskov and colleagues showed that neuronal IFNβ signaling is protective against PD-like phenotypes and accumulation of PD-related proteins in mice (Ejlerskov et al, 2015; Tresse et al, 2021). Conversely, in an ALS model, type I IFN signaling is considered detrimental because homozygous deletion of *Ifnar1* in *SOD1*^G93A^ mice extended the life span (Wang et al, 2011). However, this interpretation warrants careful reconsideration. Notably, the same study showed that heterozygous *Ifnar1* deletion extended survival time even more than complete *Ifnar1* knockout in *SOD1*^G93A^ mice, suggesting that partial, rather than absent, type I IFN signaling may be optimal. Furthermore, type I IFN signaling may have a disease stage-dependent protective role in ALS, particularly at disease onset. Our study showed that *SOD1*^G93A^/Tbk1^+/−^ mice, which exhibit an earlier onset than *SOD1*^G93A^ mice (Brenner et al, 2019), exhibited lower type I IFN signaling and more ubiquitinated protein accumulation (Fig. 7), suggesting that type I IFN signaling may be protective during the initial stages of ALS. Supporting this concept, transcriptomic analysis of spinal motor neurons from two distinct *SOD1*^G93A^ strains (C57 and 129 Sv) revealed that the C57 strain, which exhibits slower disease progression, expressed higher levels of type I IFN-responsive genes at disease onset compared to the faster-progressing 129 Sv strain (Nardo et al, 2013). The mechanism underlying IFNAR1 downregulation in ALS motor neurons also warrants discussion. A previous report demonstrated that viruses downregulate IFNAR1 via ER stress as a viral survival strategy (Liu et al, 2009). Consistently, we found that ER stress induced by thapsigargin reduced IFNAR1 levels in NSC-34 cells and iPSC-LMNs (Fig. 5G), suggesting that ER stress, which is known to occur in ALS motor neurons (Zhao et al, 2022), may similarly contribute to IFNAR1 downregulation and thereby impair protective type I IFN signaling during disease progression.

The present study demonstrated that the immunoproteasome is protective against TDP-43 accumulation driven by aggregation-prone monomeric TDP-43 in vitro and possibly also protective against polyubiquitinated protein accumulation in vivo. However, whether the immunoproteasome is protective throughout the overall ALS/FTD disease course remains an open question. Additionally, the expression levels of immunoproteasome subunits and IFNβ in patient tissues remain uncharacterized, which constitutes a limitation of this study. A recent study using immunoproteasome subunit knockout mice demonstrated that the immunoproteasome is important for proteostasis in aged mice and the lack of the immunoproteasome causes epilepsy (Leister et al, 2024). Further studies with immunoproteasome-deficient ALS/FTD models using animals and iPSC-derived neurons or organoids will be required to address the question.

In conclusion, we demonstrated that TBK1 confers neuroprotection against proteostatic stress, particularly TDP-43 pathology, through activation of the immunoproteasome pathway. Furthermore, we identified IFNβ as an inducer of the immunoproteasome in neurons, including human iPSC-derived lower motor neurons. Although further validation is required, this study is the first to demonstrate that inducing immunoproteasome activity through the TBK1-IFNβ axis in neurons may represent a promising therapeutic strategy for ALS/FTD.

## Methods

**Table:** Reagents and tools table

Reagent/resource	Reference or source	Identifier or catalog number
**Experimental models**		
Neuro2a cells (*M. Musculus*)	ATCC	CCL-131
NSC-34 cells (*M. Musculus*)	A gift from Dr. Neil Cashman	n/a
HEK293 cells (*H. sapiens*)	ATCC	CRL-1573
HeLa cells (*H. sapiens*)	ATCC	CCL-2
SH-SY5Y cells (*H. sapiens*)	ATCC	CRL-2266
KOLF2.1 J cells (*H. sapiens*, iPSC)	The Jackson Laboratory	JIPSC001000
C57BL/6J mice	CLEA Japan	C57BL/6JJcl
B6.Cg-Tg(SOD1*G93A)1Gur/J mice	The Jackson Laboratory	004435
Tbk1, Ikbke, and Tnf triple knockout mice	Laboratory Animal Resource Bank, National Institute of Biomedical Innovation, Health, and Nutrition	nbio156
**Recombinant DNA**		
pEGFP-N1	Takara Bio	6085-1
pPur vector	Takara Bio	631601
pTRE-Tight vector	Takara Bio	631059
pTet-On Advanced vector	Takara Bio	630930
pCAG-iCre	A gift from Dr. Wilsom Wong (from Addgene)	89573
CLYBL-TO-hNGN2-BSD-mApple	A gift from Dr. Michael Ward (from Addgene)	124229
**Antibodies**		
rat monoclonal anti-Myc (9E1) [1:1000 for immunofluorescence (IF)]	Proteintech	9E1
Rabbit monoclonal anti-phosphorylated TBK1 Ser172 (D52C2) (1:50)	Cell Signaling Technology	5483S
Rat monoclonal anti-HA (7C9) (1:1000)	Proteintech	7C9
Rabbit monoclonal anti-TDP-43 (E2G6G) (1:50)	Cell Signaling Technology	89718S
Goat polyclonal anti-choline Acetyltransferase (ChAT) (1:100)	Merck Millipore	AB144P
Rabbit monoclonal anti-TBK1 (E8I3G) (1:1000)	Cell Signaling Technology	38066S
Rabbit monoclonal anti-TBK1 (D1B4) (1:1000)	Cell Signaling Technology	3504S
Rabbit polyclonal anti-FLAG-tag [1:4000 for immunoblotting (IB), 1:2500 for IF]	Medical & Biological Laboratories	PM020
Mouse monoclonal anti-β-actin (1:5000)	Sigma-Aldrich	A5441
Mouse monoclonal anti-phosphorylated TDP-43 Ser409/410 (11-9) (1:2000 for IB, 1:2500 for IF)	Cosmo Bio	TIP-PTD-M01
Rat monoclonal anti-phosphorylated p62 Ser403 (4F6) (1:500)	Medical & Biological Laboratories	D343-3
Rabbit polyclonal anti-LC3 (1:1000)	Medical & Biological Laboratories	PM036
Mouse monoclonal anti-Ubiquitin (FK2) (1:1000)	Medical & Biological Laboratories	D058-3
Mouse monoclonal anti-Myc-tag (9B11) (1:1000 for IB)	Cell Signaling Technology	2276S
Rabbit polyclonal anti-RFP (1:1000)	Medical & Biological Laboratories	PM005
Mouse monoclonal anti-ISG15 (F-9) (1:250 for IB, 1:100 for IF)	Santa Cruz Biotechnology	sc-166755
Mouse monoclonal anti-HB9 (1:100)	Developmental Studies Hybridoma Bank	81.5C1-c
Rabbit monoclonal anti-ISL1 (M20C8) (1:500)	Selleck Chemicals	F1691
Chicken polyclonal anti-MAP2 (1:2000)	Abcam	ab5392
Mouse monoclonal anti-Neurofilament H (NF-H) (SMI 32) (1:500)	BioLegend	801701
Mouse monoclonal anti-NeuN (A60) (1:500)	Merck Millipore	MAB377
Mouse monoclonal anti-IFNAR1 (H-11) (1:100 for IB, 1:50 for IF)	Santa Cruz Biotechnology	sc-7391
Rat monoclonal anti-CTIP2 (25B6) (1:500)	abcam	ab18465
Rabbit polyclonal anti-IBA1 (1:200)	Fujifilm Wako Pure Chemical Corporation	019-19741
Rabbit polyclonal anti-TDP-43 (1:1000)	Proteintech	10782-2-AP
Mouse monoclonal anti-CHOP (L63F7) (1:1000)	Cell Signaling Technology	2895S
Mouse monoclonal anti-PSMB8 (D-2) (1:500 for IB)	Santa Cruz Biotechnology	sc-374089
Mouse monoclonal anti-PSMB9 (G-2) (1:500)	Santa Cruz Biotechnology	sc-373971
Rabbit polyclonal anti-PSMB8 (1:1000 for IF)	Proteintech	14859-1-AP
Rabbit polyclonal anti-human SOD1 [raised in our laboratory against a recombinant human SOD1 peptide (aa 24–36)]	n/a	n/a
Rabbit polyclonal anti-p62 (1:1000)	Medical & Biological Laboratories	PM045
Rabbit polyclonal anti-phosphorylated STING Ser366 (1:100)	Affinity Biosciences	AF7416
Mouse monoclonal anti-FLAG antibody (1 µg per sample for co-IP)	Medical & Biological Laboratories	M185-3L
Alexa Fluor–conjugated and horseradish peroxidase (HRP)-conjugated secondary antibodies	Thermo Fisher Scientific or Jackson ImmunoResearch Laboratories	n/a
**Oligonucleotides and other sequence-based reagents**		
qPCR_hTBK1_F (Human)	This paper	AGCAGATCAGGAAAAGTGTTGG
qPCR_hTBK1_R (Human)	This paper	GAAATGTTGTGCCAGCCTTCC
qPCR_Irf3_F	This paper	CTACACTCTGTGGTTCTGC
qPCR_Irf3_R	This paper	GTAGGAACAACCTTGACCA
qPCR_Sting_F	This paper	ACTCTTCTGCCGGACACTTG
qPCR_Sting_R	This paper	CCGTCTGTGGGTTCTTGGTAG
qPCR_Ifna1_F	Lianna Li & Barbara Sherry, Virology, 2010	TGAAGGACAGGAAGGACTTTG
qPCR_Ifna1_R	Lianna Li & Barbara Sherry, Virology, 2010	GAATGAGTCTAGGAGGGTTGT
qPCR_Ifnb1_F	Komine, Cell Death & Differ, 2018	CGGACTTCAAGATCCCTATGGA
qPCR_Ifnb1_R	Komine, Cell Death & Differ, 2018	TGGCAAAGGCAGTGTAACTCTTC
qPCR_Tnf_F	Balka, Cell Rep, 2020	CCAAATGGCCTCCCTCTCAT
qPCR_Tnf_R	Balka, Cell Rep, 2020	TGGTGGTTTGCTACGACGTG
qPCR_Il1b_F	Balka, Cell Rep, 2020	TTGACGGACCCCAAAAGATG
qPCR_Il1b_R	Balka, Cell Rep, 2020	CAGCTTCTCCACAGCCACAA
qPCR_Il6_F	Balka, Cell Rep, 2020	CCAGAAACCGCTATGAAGTTCC
qPCR_Il6_R	Balka, Cell Rep, 2020	CGGACTTGTGAAGTAGGGAAGG
qPCR_Psmb5_F	This paper	CCTTCAAGTTTCTCCATGGA
qPCR_Psmb5_R	This paper	TCATGGTGCCCATAGACAG
qPCR_Psmb6_F	This paper	GCCTTAGCTGTTCGTCGAG
qPCR_Psmb6_R	This paper	TAGAACCACGCCCCCATTAAA
qPCR_Psmb7_F	This paper	GTGTCGGTGTTTCAGCCAC
qPCR_Psmb7_R	This paper	GTGCCAGTTTTCCGAGCTTTC
qPCR_Psmb8_F	This paper	GAGTGCAGGTTGTATTATCTTCG
qPCR_Psmb8_R	This paper	CAGCATCATGTTGGAAAGCA
qPCR_Psmb9_F	This paper	TATGGTTATGTGGACGCAG
qPCR_Psmb9_R	This paper	CAGAGTGATGGCATCTGTG
qPCR_Psmb10_F	This paper	TGTGTTCCGAGATGGAGTC
qPCR_Psmb10_R	This paper	TGAAGTGGATCTTCTCGCAG
qPCR_Psme1_F	This paper	AAGGTGTTTGAGCTGATGAC
qPCR_Psme1_R	This paper	AAGGTGTTTGAGCTGATGAC
qPCR_Psme2_F	This paper	GGTAATCACATGGATCCAGC
qPCR_Psme2_R	This paper	CTGAGAAGTACTTGGAAATGGT
qPCR_Psme3_F	This paper	TCCACTCAGACATGAACCT
qPCR_Psme3_R	This paper	TGTAAGTGGGACCATCCAG
qPCR_Psme4_F	This paper	TTCCAAGACAGAGCACCTG
qPCR_Psme4_R	This paper	TGGAAAGTATGGTCGGCAG
qPCR_Ifit1_F	This paper	TACAGCAACCATGGGAGAG
qPCR_Ifit1_R	This paper	CTTCCATGTGAAGTGACATCTC
qPCR_Isg15_F	Komine, Cell Death & Differ, 2018	ATGAGGTCTTTCTGACGCAG
qPCR_Isg15_R	Komine, Cell Death & Differ, 2018	AGCAGCTCCTTGTCCTCCAT
qPCR_Oas1a_F	This paper	AAAGGATGGTTCCCGAGTG
qPCR_Oas1a_R	This paper	TGTCCAGTTCTCTTCTACCTG
qPCR_Ifit3_F	This paper	ATCATGAGTGAGGTCAACCG
qPCR_Ifit3_R	This paper	AAATGTTCGACCTGGTTGC
qPCR_mTbk1_F (Mouse)	This paper	CAACACATGACGGCGCATAAG
qPCR_mTbk1_R (Mouse)	This paper	GGACTAAGCGTCGTCCTTCG
qPCR_Actb_F	Horiuchi, Acta Neuropathol Com, 2024	ACAGCTTCTTTGCAGCTCCT
qPCR_Actb_R	Horiuchi, Acta Neuropathol Com, 2024	ATTCCCACCATCACACCCTG
qPCR_Rps13_F	This paper	TGAGAGGAACAGAAAGGATAAGG
qPCR_Rps13_R	This paper	TAGTATCGAGCCAATCGGTG
qPCR_Chat_F	This paper	AGACAGCCAATTGGGTCTC
qPCR_Chat_R	This paper	CAGGGCTAGAGTTGACTGG
qPCR_Gfap_F	Komine, Cell Death & Differ, 2018	TGACCGCTTTGCTAGCTACATC
qPCR_Gfap_R	Komine, Cell Death & Differ, 2018	CCAGCGCCTTGTTTTGCT
qPCR_Tmem119_F	Maekawa, Sci Rep, 2025	CACCCAGAGCTGGTTCCATAG
qPCR_Tmem119_R	Maekawa, Sci Rep, 2025	GGGAGTGACACAGAGTAGGC
qPCR_Mbp_F	Horiuchi, Acta Neuropathol Com, 2024	CACACGAGAACTACCCATTATGGC
qPCR_Mbp_R	Horiuchi, Acta Neuropathol Com, 2024	GTGTTCGAGGTGTCACAATGTTCT
Negative Control DsiRNA	Integrated DNA Technologies	51-01-14-04
Sting DsiRNA	Integrated DNA Technologies	mm.Ri.Tmem173.13.3
Ifr3 DsiRNA	Integrated DNA Technologies	mm.Ri.Irf3.13.1
Ifnb1 DsiRNA #1	Integrated DNA Technologies	mm.Ri.Ifnb1.13.1
Ifnb1 DsiRNA #2	Integrated DNA Technologies	mm.Ri.Ifnb1.13.3
Stealth RNAi™ siRNA Negative Control, Med GC	Thermo Fisher Scientific	12935300
Tbk1 Stealth Select RNAi™ siRNA	Thermo Fisher Scientific	MSS226225
CLYBL crRNA	Integrated DNA Technologies	AUGUUGGAAGGAUGAGGAAAGUUUUAGAGCUAUGCU (5’ → 3’)
tracrRNA	Integrated DNA Technologies	1072532
**Chemicals, enzymes and other reagents**		
BX795	Sigma-Aldrich	SML0694
bafilomycin A1	Sigma-Aldrich	B1793
MG132	Peptide Institute	3175-v
tofacitinib	Selleck Chemicals	S2789
recombinant mouse IFNβ	BioLegend	581302
recombinant human IFNβ	Proteintech	HZ-1298
thapsigargin	Selleck Chemicals	S7895
lactacystin	Peptide Institute	4368-v
ONX-0914	Cayman Chemical	16271
G-418	Nacalai Tesque	09380-86
puromycin	InvivoGen	ant-pr-1
blasticidin	Sigma-Aldrich	SBR00022-1ML
DMEM	Thermo Fisher Scientific	C11965500
DMEM/F12	Thermo Fisher Scientific	11330032
fetal bovine serum (FBS)	Thermo Fisher Scientific	10270106
penicillin and streptomycin	Thermo Fisher Scientific	15140122
ITS-G supplement	Fujifilm Wako Pure Chemical Corporation	090-06741
0.25% (w/v) trypsin–1 mM EDTA solution	Fujifilm Wako Pure Chemical Corporation	201-16945
poly-D-lysine	Thermo Fisher Scientific	A3890401
collagen	KOKEN	IPC-30
N,N-dibutyladenosine 3’,5’-phosphoric acid (dbcAMP)	Nacalai Tesque	11540-61
mTeSR Plus	STEMCELL Technologies	ST-100-0276
vitronectin	Thermo Fisher Scientific	A31804
Versene	Thermo Fisher Scientific	15040066
Accutase	Thermo Fisher Scientific	A1110501
Geltrex	Thermo Fisher Scientific	A1413302
N2 Supplement	Thermo Fisher Scientific	17502048
GlutaMAX	Thermo Fisher Scientific	35050061
non-essential amino acids	Thermo Fisher Scientific	11140050
glucose	Nacalai Tesque	16974-54
LDN-193189	Selleck Chemicals	S7507
SB431542	Selleck Chemicals	S1067
SAG (Smoothened agonist)	Selleck Chemicals	S7779
retinoic acid	Sigma-Aldrich	R2625-50MG
doxycycline (Dox)	Sigma-Aldrich	D9891-1G
Y-27632	Selleck Chemicals	S1049
laminin	Thermo Fisher Scientific	23017015
Neurobasal Plus Medium	Thermo Fisher Scientific	A3582901
B-27 Plus Supplement	Thermo Fisher Scientific	A3582801
CultureOne Supplement	Thermo Fisher Scientific	A3320201
GSK2879552	Selleck Chemicals	S7796
EPZ-5676	Selleck Chemicals	S7062
N-methyl-D-aspartic acid (NMDA)	Selleck Chemicals	S7072
Bay K 8644	Selleck Chemicals	S7924
BDNF	PeproTech	450-02-10ug
GDNF	PeproTech	450-10-10ug
CNTF	PeproTech	AF-450-13-20ug
uridine	Sigma-Aldrich	U3003-5G
5-fluoro-2’-deoxyuridine	Sigma-Aldrich	F0503-100MG
aphidicolin	Tocris Bioscience	5736
dithiobis(succinimidyl propionate) (DSP)	MedChemExpress	HY-118759
glycine	Fujifilm Wako Pure Chemical Corporation	077-00735
cOmplete EDTA-free protease inhibitor cocktail	Roche Diagnostics GmbH	11873580001
PhosSTOP	Roche Diagnostics GmbH	4906845001
Dynabeads M-280 Sheep anti-Mouse IgG	Thermo Fisher Scientific	11201D
bovine serum albumin	Nacalai Tesque	01281-26
Immobilon Crescendo Western HRP substrate	Merck Millipore	WBLUR0500
goat serum	Jackson ImmunoResearch Laboratories	005-000-121
donkey serum	Jackson ImmunoResearch Laboratories	017-000-121
linear polyethyleneimine (PEI)	Polysciences	23966
Lipofectamine 2000	Thermo Fisher Scientific	11668019
Lipofectamine RNAiMax	Thermo Fisher Scientific	13778150
Lipofectamine Stem Transfection Reagent	Thermo Fisher Scientific	STEM00003
NeuroMag	OZ Biosciences	NM50200
Can Get Signal immunostain Immunoreaction Enhancer Solution A	TOYOBO	NKB-501
Can Get Signal immunostain Immunoreaction Enhancer Solution B	TOYOBO	NKB-601
HistoVT One	Nacalai Tesque	06380-05
TrueBlack Lipofuscin Autofluorescence Quencher	Biotium	23007
Fluoromount/Plus	Diagnostic BioSystems	K048
Tissue-Tek OCT compound	Sakura Finetek Japan	4583
TRIzol Reagent	Thermo Fisher Scientific	15596018
TB Green Premix Ex Taq^TM^ II (Tli RNaseH Plus)	Takara Bio	RR820A
toluidine blue O	FUJIFILM Wako Pure Chemical Corporation	535-05542
Ac-ANW-AMC	Cayman Chemical	26640
recombinant Cas9	Integrated DNA Technologies	1081060
**Software**		
MultiGauge	Cytiva	n/a
Fiji (version 2.1.0/1.54j)	Schindelin et al, 2012	n/a
ZEN	Carl Zeiss AG	n/a
GraphPad Prism 6	GraphPad Software	n/a
RStudio (version 2023.09.1 + 494)	R Foundation for Statistical Computing	n/a
**Other**		
Lab-Tek II 4- or 8-well chamber slides	Thermo Fisher Scientific	154526PK 154534PK
Falcon 4- or 8-well culture slides	Corning	354114 354118
Super Magnetic Plate	OZ Biosciences	MF-10000
PEN foil-covered glass slides	Leica Biosystems	11505158
Immobilon-P PVDF Membrane	Merck Millipore	IPVH00010
Bio-Rad Protein Assay Kit I	Bio-Rad	5000001
Micro BCA Protein Assay Kit	Thermo Fisher Scientific	23235
RNeasy Micro Kit	Qiagen	74004
PrimeScript™ RT reagent Kit (Perfect Real Time)	Takara Bio	RR037A
FlexAble 2.0 CoraLite® Plus 488 Antibody Labeling Kit for Rabbit IgG	Proteintech	KFA501
FlexAble 2.0 CoraLite® Plus 555 Antibody Labeling Kit for Rabbit IgG	Proteintech	KFA502
LAS-4000 mini	Cytiva	
LuminoGraph Ⅲ Light	ATTO	
LSM-900	Carl Zeiss AG	
Leica LMD7000 laser microdissection system	Leica Biosystems	
Agilent 2100 Bioanalyzer	Agilent Technologies	
Thermal Cycler Dice Real Time System II	Takara Bio	
Infinite 200 PRO	Tecan Schweiz AG	

### Antibodies, chemicals, enzymes, and other reagents

The antibodies, chemicals, enzymes, and other reagents used in this study are listed in the Reagents and tools table.

### Plasmids and siRNAs

Expression plasmids of human TDP-43 (hTDP-43) (WT, 6M, L27/28A, and ΔNLS)-3×FLAG and hTDP-43 (WT and 6M)-mCherry were generated as previously described (Oiwa et al, 2023). Based on the hTDP-43 (WT)-3×FLAG plasmid, hTDP-43 (C173/175S, A315T, and M337V)-3×FLAG constructs were generated by site-directed mutagenesis. To generate hTDP-43 (WT and 6M)-EGFP constructs, cDNA encoding hTDP-43 was subcloned into the pEGFP-N1 vector (#6085-1, Takara Bio, Shiga, Japan). For expression in iPSCs and iPSC-LMNs, cDNA encoding hTDP-43 (WT and 6M)-3×FLAG followed by the woodchuck hepatitis virus posttranscriptional regulatory element (WPRE) was subcloned into a pCAGGS backbone derived from pCAG-iCre (Addgene #89573; a gift from Dr. Wilson Wong). Based on these constructs, Myc- and HA-hTDP-43 constructs were generated by removing the C-terminal 3×FLAG tag and adding an N-terminal Myc or HA tag. An expression plasmid of Myc-human TBK1 (WT) was generated as previously described (Watanabe et al, 2023). Based on this plasmid, ALS/FTLD-linked mutations of TBK1 were introduced by site-directed mutagenesis. An mCherry-TBK1 construct was generated as previously described (Watanabe et al, 2020). To establish stable lines expressing Dox-inducible hTDP-43 (WT or 6M)-3×FLAG, pTRE-Tight-hTDP-43 (WT or 6M)-3×FLAG-SV40-Puro^R^ was generated by subcloning cDNAs encoding hTDP-43 (WT or 6M)-3×FLAG and the SV40 early promoter–puromycin resistance gene–polyadenylation signal sequence from the pPur vector (#631601, Takara Bio) into the pTRE-Tight vector (#631059, Takara Bio). Predesigned 27mer Dicer-substrate short interfering RNAs (siRNAs) specific for *Sting*, *Irf3*, and *Ifnb1*, and a negative control siRNA were purchased from Integrated DNA Technologies (IDT) (Coralville, IA, USA). Predesigned Stealth RNAi™ siRNA specific for *Tbk1* and negative control siRNA were purchased from Thermo Fisher Scientific (Waltham, MA, USA).

### Cell line and mouse primary spinal neuron culture, and transfection

Mouse neuroblastoma Neuro2a (ATCC, Manassas, VA, USA), mouse motor neuron–like hybrid cell line NSC-34 (a kind gift from Dr. Neil Cashman, the University of British Columbia), HEK293 (ATCC), and HeLa (ATCC) cells were maintained in Dulbecco’s modified Eagle’s medium (DMEM) containing 4.5 g/L glucose supplemented with 10% (v/v) fetal bovine serum (FBS), 100 U/mL penicillin, and 100 μg/mL streptomycin (Thermo Fisher Scientific). Human neuroblastoma SH-SY5Y (ATCC) cells were maintained in a 1:1 mixture of DMEM and Ham’s F12 (DMEM/F12) (Thermo Fisher Scientific) supplemented with 10% (v/v) FBS, penicillin, streptomycin, and ITS-G supplement (Fujifilm Wako Pure Chemical Corporation, Osaka, Japan). Mouse primary spinal neurons were isolated from embryonic spinal cords (E14–E15) of C57BL/6 J mice obtained from CLEA Japan (Tokyo, Japan) and cultured on plates coated with 0.1 mg/mL poly-D-lysine (Thermo Fisher Scientific) in medium consisting of Neurobasal Plus Medium, 1× B-27 Plus Supplement, 0.5 mM GlutaMAX, 1× CultureOne Supplement, 100 U/mL penicillin, and 100 μg/mL streptomycin (all from Thermo Fisher Scientific). On culture day 1, the medium was replaced with fresh medium, and thereafter, half of the medium was replaced every 3–4 days. For immunofluorescence of Neuro2a cells, Lab-Tek II 4- or 8-well chamber slides (Thermo Fisher Scientific) and Falcon 4- or 8-well culture slides (Corning, Corning, NY, USA) were coated with 40 μg/mL atelocollagen (KOKEN, Tokyo, Japan) at 37 °C for 30 min, after which cells were cultured on the coated slides.

In the co-culture experiments, Neuro2a cells transfected with the indicated plasmids were detached using 0.25% (w/v) trypsin–1 mM EDTA solution (Fujifilm Wako Pure Chemical Corporation) 6 h after transfection. The cells were then replated at a 1:1 ratio onto atelocollagen-coated slides or culture plates in the indicated combinations, using a differentiation medium consisting of DMEM containing 4.5 g/L glucose, 2% (v/v) FBS, and 2.5 mM *N*,*N*-dibutyladenosine 3’,5’-phosphoric acid (dbcAMP) (Nacalai Tesque, Kyoto, Japan).

In the experiments using conditioned media, the culture medium of Neuro2a cells, which were transfected with either mock or TBK1 plasmids, was replaced with fresh medium 6 h after transfection to remove residual plasmids, and conditioned media were collected after an additional 36-h incubation. The collected media were centrifuged at 1000 rpm for 5 min to remove dead cells, and the supernatant was stored at 4 °C for up to 24 h before use. Six hours after transfection with TDP-43 (WT or 6M), the culture medium of recipient Neuro2a cells was replaced with the collected conditioned media.

Neuro2a and NSC-34 cells stably expressing Dox-inducible hTDP-43 (WT or 6M)-3×FLAG were generated through two sequential rounds of transfection and antibiotic selection. The first transfection was performed using linearized pTet-On Advanced vector (#630930, Takara Bio), followed by positive selection with 1 mg/mL G-418 (Nacalai Tesque) for 2 weeks. The second transfection was then performed using linearized pTRE-Tight-hTDP-43 (WT or 6M)-3×FLAG-SV40-Puro^R^, followed by positive selection with 2 μg/mL puromycin (InvivoGen, San Diego, CA, USA) for an additional two weeks.

Plasmid transfection was performed in Neuro2a, NSC-34, SH-SY5Y and HEK293 cells using Lipofectamine 2000 (Thermo Fisher Scientific). For HeLa cells, linear polyethyleneimine (PEI) (Polysciences, Warrington, PA, USA) was used instead. Six hours after transfection, the media were replaced with fresh ones. siRNA transfection was performed using Lipofectamine RNAiMax (Thermo Fisher Scientific).

### iPSC culture, differentiation, and transfection

A human iPSC line from a healthy donor, KOLF2.1J (Pantazis et al, 2022), was purchased from The Jackson Laboratory (Bar Harbor, ME, USA). iPSCs were maintained in mTeSR Plus medium (STEMCELL Technologies, Vancouver, BC, Canada) on culture plates coated with vitronectin (Thermo Fisher Scientific) and routinely passaged using Versene (Thermo Fisher Scientific). For rapid and efficient differentiation into lower motor neurons (LMNs), we established an iPSC-NGN2 line in which a Tet-On–inducible neurogenin-2 (NGN2) expression cassette was inserted into the CLYBL safe harbor locus in KOLF2.1 J cells by CRISPR-mediated genome editing, based on a previously described method (Fernandopulle et al, 2018). CLYBL-TO-hNGN2-BSD-mApple (Addgene #124229; a gift from Dr. Michael Ward) was used as a donor DNA template after replacing the mApple sequence with mTagBFP2, which was synthesized using the gBlocks Gene Fragments service (IDT). The donor plasmid was introduced into KOLF2.1 J cells together with a ribonucleoprotein (RNP) complex consisting of crRNA (5’-AUGUUGGAAGGAUGAGGAAAGUUUUAGAGCUAUGCU-3’) (IDT), Alt-R^®^ CRISPR-Cas9 tracrRNA (IDT), and Alt-R^®^ S.p. HiFi Cas9 Nuclease V3 (IDT), using Lipofectamine Stem Transfection Reagent (Thermo Fisher Scientific). After positive selection with 10 μg/mL blasticidin (Sigma-Aldrich, St. Louis, MO, USA), iPSCs were dissociated into single cells using Accutase (Thermo Fisher Scientific) and plated onto culture dishes. Monoclonal colonies with uniform mTagBFP2 fluorescence were isolated by manual colony picking and expanded.

To differentiate iPSC-NGN2 cells into LMNs, we followed a recently established protocol (Limone et al, 2023) with minor modifications. Briefly, subconfluent iPSC-NGN2 cells were dissociated with Accutase and plated onto culture plates coated with Geltrex (Thermo Fisher Scientific) in an induction medium consisting of DMEM/F12, 1× N2 Supplement, 1× GlutaMAX, 1× non-essential amino acids (all from Thermo Fisher Scientific), 0.5% glucose (Nacalai Tesque), 200 nM LDN-193189 (#S7507), 10 μM SB431542 (#S1067), 2 μM SAG (Smoothened agonist; #S7779) (all from Selleck Chemicals, Houston, TX, USA), 2 μM retinoic acid (RA) (#R2625-50MG), and 2 μg/mL doxycycline (Dox) (#D9891-1G) (both from Sigma-Aldrich), with the addition of 10 μM Y-27632 (#S1049, Selleck Chemicals). On induction day 1, the medium was replaced with fresh induction medium without Y-27632, in which the concentrations of LDN-193189, SAG, and RA were reduced to 100 nM, 1 μM, and 1 μM, respectively. On induction day 3, the differentiated cells were dissociated with Accutase and replated onto culture plates or chamber slides coated with 0.1 mg/mL poly-D-lysine, Geltrex, and 10 μg/mL laminin (Thermo Fisher Scientific) in a maturation medium consisting of Neurobasal Plus Medium, 1× B-27 Plus Supplement, 1× N2 Supplement, 1× GlutaMAX, 1× non-essential amino acids, 0.5% glucose, 1× CultureOne Supplement, 1 μM SAG, 1 μM RA, 1 μM GENtoniK cocktail [1 μM GSK2879552 (#S7796), 1 μM EPZ-5676 (#S7062), 1 μM N-methyl-D-aspartic acid (NMDA; #S7072), and 1 μM Bay K 8644 (#S7924) (all from Selleck Chemicals)] (Hergenreder et al, 2024), 1 μg/mL laminin, 10 ng/mL BDNF, 10 ng/mL GDNF, 10 ng/mL CNTF (all from PeproTech, Cranbury, NJ, USA), 2 μg/mL Dox, and a mitotic inhibitor [1 μM uridine (#U3003-5G) + 1 μM 5-fluoro-2’-deoxyuridine (#F0503-100MG) (both from Sigma-Aldrich) or 1 μM aphidicolin (#5736, Tocris Bioscience, Bristol, UK)]. On induction days 7, 10, and 13, half of the medium was replaced with fresh maturation medium lacking SAG, RA, Dox, and mitotic inhibitors.

iPSC-LMNs were transfected on induction day 10 using magnetic nanobeads and a magnetic plate (NeuroMag and Super Magnetic Plate, OZ Biosciences, Marseille, France) according to the manufacturer’s instructions. Briefly, plasmid DNA was incubated with NeuroMag in Neurobasal Plus Medium for 15 min and then added dropwise to the cultures. The iPSC-derived LMNs were then incubated on a magnetic plate for 30 min in a CO₂ incubator. After removal of the magnet, the cells were maintained in culture until induction day 15. Undifferentiated iPSCs were transfected with Lipofectamine Stem Transfection Reagent according to the manufacturer’s instructions.

All experimental procedures using human iPSCs were approved by the ethics committee of Nagoya University (approval number #401).

### Animals

Transgenic mice expressing human *SOD1* gene with G93A mutation [B6.Cg-Tg(SOD1*G93A)1Gur/J] were obtained from The Jackson Laboratory. The age of 3.5 months was defined as the onset stage based on our two independent studies using this model (Komine et al, 2018; Watanabe et al, 2024). *Tbk1* heterozygous knockout mice were established by breeding *Tbk1*, *Ikbke*, and *Tnf* triple knockout mice (#nbio156), which were obtained from Laboratory Animal Resource Bank, National Institute of Biomedical Innovation, Health, and Nutrition (Osaka, Japan). C57BL/6J mice obtained from CLEA Japan (Tokyo, Japan) were used as wild-type control mice. All mice were housed under specific pathogen-free conditions with a 12-h light/dark cycle at 23 ± 1 °C; 50 ± 5% humidity, with free access to food and water. Mice were handled according to the guidelines established by the Animal Care and Use Committee of Nagoya University. All experimental procedures involving genetically modified animals were approved by both the Animal Care and Use Committee and the Recombinant DNA Experiment Committee of Nagoya University (approval numbers RIEM250022, RIEM250023, and #143, respectively).

### Postmortem human tissues

Spinal cord specimens were obtained by autopsy with informed consent from six patients with ALS and four patients with non-ALS neurological diseases (used as controls). Detailed clinical information for all patients is provided in Appendix Table S1. ALS was diagnosed according to the El Escorial clinical criteria established by the World Federation of Neurology, along with the pathological presence of TDP-43 inclusions (Brooks et al, 2000). All ALS cases included in this study were confirmed as sporadic through detailed assessment of family history. Ethical approval for the collection and use of human tissues was obtained from the ethics committee of Nagoya University (approval number #399).

### Co-immunoprecipitation

Co-immunoprecipitation (co-IP) was performed using Dynabeads M-280 Sheep anti-Mouse IgG (Thermo Fisher Scientific) according to the manufacturer’s instructions. Briefly, Neuro2a cells (6-well plate format) transfected with hTDP-43 (WT, 6M, or ΔNLS)-3×FLAG were harvested in ice-cold co-IP lysis buffer [50 mM Tris-HCl (pH 7.4), 150 mM NaCl, 2 mM MgCl₂, 0.5% (v/v) NP-40, and 1 mM dithiothreitol (DTT)] supplemented with cOmplete EDTA-free protease inhibitor cocktail (Roche Diagnostics GmbH, Mannheim, Germany) and lysed by brief sonication on ice. After centrifugation at 15,000×*g* for 10 min at 4 °C, the supernatant was collected and incubated with a mouse monoclonal anti-FLAG antibody (1 µg per sample; #M185-3L, Medical & Biological Laboratories, Tokyo, Japan) for 1.5 h at 4 °C with gentle agitation. Meanwhile, 25 µL per sample of Dynabeads M-280 Sheep anti-Mouse IgG were transferred into a 1.5 mL tube, and the suspension buffer was replaced with co-IP lysis buffer. The Dynabeads were then mixed with the cell lysate preincubated with the FLAG antibody and incubated overnight at 4 °C with gentle agitation. The beads were washed three times with co-IP lysis buffer, and the immunoprecipitated proteins were eluted with 2× SDS sample buffer at 95 °C for 5 min and subjected to immunoblotting.

For DSP crosslinking co-IP, live cells were washed twice with PBS and incubated in PBS containing 0.5 mM DSP (#HY-118759, MedChemExpress, Monmouth Junction, NJ, USA) for 30 min. Then, 0.2 M glycine in PBS was added at 1/10 volume, and the cells were incubated for 15 min to quench DSP-mediated crosslinking. After washing with PBS, cells were harvested in co-IP lysis buffer lacking DTT and subjected to the co-IP procedure described above.

### Detergent solubility fractionation and immunoblotting

For detergent solubility fractionation, Neuro2a cells or mouse lumbar spinal cords were collected in ice-cold TNE buffer [50 mM Tris-HCl (pH 7.4), 150 mM NaCl, 1 mM EDTA] containing 1% (v/v) Triton X-100, cOmplete EDTA-free protease inhibitor cocktail, and PhosSTOP (Roche), and lysed by brief sonication on ice. After centrifugation at 15,000×*g* for 10 min at 4 °C, the supernatants were collected as the soluble fractions. The remaining pellets were washed twice with the lysis buffer and resuspended in TNE buffer containing 2% (w/v) sodium dodecyl sulfate (SDS) by brief sonication. After centrifugation at 15,000×*g* for 5 min at room temperature, the resulting supernatants were collected as the insoluble fractions. The protein concentration of each sample was determined by using a BCA assay kit (Bio-Rad Laboratories, Richmond, CA, USA). Equal amounts of total protein were separated by sodium dodecyl sulfate-polyacrylamide gel electrophoresis (SDS-PAGE) and transferred to a polyvinylidene difluoride membrane (Immobilon-P; Merck Millipore, Burlington, MA, USA). The membrane was incubated with a blocking buffer [50 mM Tris-HCl (pH 7.4), 150 mM NaCl, 0.05% (v/v) Tween-20, and 2% (w/v) bovine serum albumin (Nacalai Tesque)], followed by incubation with primary antibodies diluted in the blocking buffer at 4 °C for at least 6 h. Bound antibodies were detected using horseradish peroxidase-conjugated secondary antibodies and Immobilon Crescendo Western HRP substrate (Merck Millipore). Images were obtained by LAS-4000 mini (Cytiva, Tokyo, Japan) or LuminoGraph Ⅲ Light (ATTO, Tokyo, Japan) and analyzed by MultiGauge (Cytiva) or Fiji software (Schindelin et al, 2012).

### Immunofluorescence

Cells were fixed with 4% (w/v) paraformaldehyde (PFA) for 20 min at room temperature, and permeabilized with TBS [50 mM Tris-HCl (pH 7.4), 150 mM NaCl] containing 0.5% (v/v) Triton X-100 for 30 min. After blocking with the blocking buffer [TBS containing 0.3% (v/v) Triton X-100 and 5% goat or donkey serum (Jackson ImmunoResearch Laboratories, West Grove, PA, USA)] for an hour, the cells were incubated with primary antibodies diluted in the blocking buffer overnight at 4 °C. After three washes with the wash buffer [TBS containing 0.3% (v/v) Triton X-100], cells were incubated with Alexa-conjugated secondary antibodies and 1 µg/mL DAPI diluted in the blocking buffer for 2 h at room temperature. Finally, the samples were mounted with Fluoromount/Plus (Diagnostic BioSystems, Pleasanton, CA, USA). For PSMB8 staining, cells were fixed with ice-cold methanol for 15 min at 4 °C. For p-TBK1, HB9, ChAT, and PSMB8 staining, Can Get Signal immunostain Immunoreaction Enhancer Solution B (TOYOBO, Osaka, Japan) was used as the reaction buffer to enhance the immunofluorescent signal. FlexAble 2.0 CoraLite® Plus 488 Antibody Labeling Kit for Rabbit IgG (#KFA501, Proteintech, Rosemont, IL, USA) and FlexAble 2.0 CoraLite® Plus 555 Antibody Labeling Kit for Rabbit IgG (#KFA502, Proteintech) were used to fluorescently label anti-p-TBK1 and anti-TDP-43 (E2G6G) antibodies, respectively, for co-immunostaining of p-TBK1 and monomeric TDP-43.

Mice were deeply anesthetized using a mixture of three anesthetic agents (medetomidine, midazolam, and butorphanol) and transcardially perfused with PBS for 5 min, followed by perfusion fixation with 4% (w/v) PFA in 0.1 M phosphate buffer for 10 min. After an additional overnight immersion fixation in the same fixative, brains and spinal cords were transferred to 30% (w/v) sucrose in PBS and incubated for at least 2 days. Tissues were then embedded in Tissue-Tek OCT compound (Sakura Finetek Japan, Tokyo, Japan) and frozen at −80 °C until use. Cryosections of 20 μm thickness were prepared using a cryostat (Leica Biosystems, Wetzlar, Germany) and immunostained in the same manner as cultured cells. For IFNAR1 staining, Can Get Signal immunostain Immunoreaction Enhancer Solution A (TOYOBO) was used as the reaction buffer to enhance the immunofluorescent signal.

For human tissues, 3 μm sections were prepared from paraffin-embedded cervical or lumbar spinal cord samples. Prior to staining, deparaffinization and antigen retrieval were performed using HistoVT One (Nacalai Tesque) at 90 °C for 40 min. Immunostaining was performed in the same manner as cultured cells and mouse tissue sections, except that PBS was used instead of TBS containing Triton X-100 for all procedures. Prior to mounting, lipofuscin autofluorescence was quenched using TrueBlack Lipofuscin Autofluorescence Quencher (Biotium, Fremont, CA, USA). The fluorescence intensity of IFNAR1 was quantified using Fiji software.

All images were obtained using a confocal laser scanning microscope (LSM-900; Carl Zeiss AG, Oberkochen, Germany) with the equipped software (ZEN; Carl Zeiss AG).

### RNA isolation, laser microdissection, and quantitative PCR

Total RNA from cells and whole lumbar spinal cords (LSCs) was extracted using TRIzol Reagent (Thermo Fisher Scientific) according to the manufacturer’s instructions. For RNA extraction from motor neurons in LSCs, laser microdissection (LMD) was performed. Briefly, fresh LSCs were dissected from murine spinal cords and rapidly frozen in Tissue-Tek OCT compound at −80 °C. Twenty μm cryosections mounted on PEN foil-covered glass slides (Leica) were fixed for 90 s in 70% ethanol pre-chilled to −30 °C, stained for 2 min in ice-cold 0.02% toluidine blue O (FUJIFILM Wako Pure Chemical Corporation), briefly rinsed in ice-cold water, and dehydrated through a graded ethanol series (70, 95, and 100%). Motor neurons, identified based on their anatomical location, morphology, and size (diameter ≥25 μm), were isolated using a Leica LMD7000 laser microdissection system and directly collected into the caps of collection tubes containing lysis buffer. Total RNA from LMD-isolated motor neurons was extracted using the RNeasy Micro Kit (Qiagen, Hilden, Germany), and RNA quality and concentration were assessed using an Agilent 2100 Bioanalyzer (Agilent Technologies, Santa Clara, CA, USA).

cDNA was synthesized from 100–500 ng (cells and whole LSCs) or 18 ng (LMD-isolated motor neurons) of total RNA using the PrimeScript™ RT reagent Kit (Perfect Real Time) (Takara Bio). Quantitative PCR (qPCR) was performed using TB Green Premix Ex Taq™ II (Tli RNaseH Plus) and Thermal Cycler Dice Real Time System II (both from Takara Bio). The primers used in this study are listed in the Reagents and Tools Table.

### Immunoproteasome activity assay

The immunoproteasome activity assay was performed as previously described (Kim et al, 2022) with minor modifications. Briefly, Neuro2a cells and iPSC-LMNs were collected in ice-cold proteasome activity lysis buffer [25 mM Tris-HCl (pH 7.4), 5 mM MgCl₂, 10% (v/v) glycerol, 1 mM freshly prepared ATP, 1 mM freshly prepared DTT, and an EDTA-free protease inhibitor cocktail] and lysed by brief sonication on ice. The lysates were then centrifuged at 15,000×*g* for 5 min at 4 °C, and the supernatants were collected. Protein concentrations were normalized using the Bradford assay (Bio-Rad Protein Assay Kit). The samples were preincubated with either DMSO or 10 μM ONX-0914 at 4 °C for 30 min. Then, 10 μL of each sample was mixed with 100 μL of proteasome activity assay buffer [50 mM Tris-HCl (pH 7.4), 1 mg/mL BSA, 1 mM EDTA, 1 mM freshly prepared ATP, 1 mM freshly prepared DTT] in a black 96-well plate, and the fluorogenic substrate Ac-ANW-AMC (#26640, Cayman Chemical, Ann Arbor, MI, USA) was added at a final concentration of 12.5 μM. After incubation at room temperature for 15 min, AMC fluorescence was measured every 3 min using a microplate reader (Infinite 200 PRO, Tecan Schweiz AG, Männedorf, Switzerland) at 360 nm excitation and 460 nm emission wavelengths.

### Statistical analysis

All semiquantitative immunoblotting data, immunofluorescence data (except for human IFNAR1 staining), qPCR data, and immunoproteasome activity assay data were analyzed by one-way ANOVA followed by Holm–Sidak’s multiple comparisons test for three or more groups and unpaired *t*-tests with Welch’s correction for comparison between two groups. Regarding statistical analysis of human IFNAR1 intensity in Fig. 5E,F, a linear mixed-effects model was used, and the significance of the fixed effect was evaluated using Satterthwaite’s approximation. For Fig. 5F, post hoc pairwise comparisons were performed with Tukey’s adjustment. Outliers [one data point in Fig. 4A (ΔNLS, insoluble FLAG fold change), two data points in Fig. 7H (*SOD1*^G93A^, one each for *Isg15* and *Oas1a*), and one data point in Fig. 7I (*SOD1*^G93A^/*Tbk1*^+/−^, *Psmb9*)] were identified using Grubbs’ test at a significance level of α = 0.05 and excluded from both graphical representations and statistical analyses. All statistical analyses were performed using GraphPad Prism (GraphPad Software, La Jolla, CA, USA) and R software.

### Graphics

Graphics were created with BioRender.com.

## Supplementary information

Appendix

Peer Review File

Source data Fig. 1

Source data Fig. 2

Source data Fig. 3

Source data Fig. 4

Source data Fig. 5

Source data Fig. 6

Source data Fig. 7

Appendix Figure S6A Figure Source Data

## Data Availability

This study includes no data deposited in external repositories. The source data of this paper are collected in the following database record: biostudies:S-SCDT-10_1038-S44319-026-00953-8.
