# Supplementary material for: TBK1 eliminates aggregation-prone monomeric TDP-43 through an IFNβ-immunoproteasome pathway in ALS models
Source: EMBO Rep. 2026 Oct 5;27(21):6251–76. doi: 10.1038/s44319-026-00953-8 (PMC13743774; doi:10.1038/s44319-026-00953-8)
Supplement: Supplementary file 1 [file 44319_2026_953_MOESM1_ESM.pdf]

Supplementary Figures and Table for

TBK1 eliminates aggregation-prone monomeric TDP-43 through an IFNβ-immunoproteasome pathway in ALS models

Shohei Sakai, Kotaro Oiwa, Yohei Iguchi, Izzet Mehmet Akcay, Seiji Watanabe, Okiru Komine, Noriko Nagata, Mai Horiuchi, Jemeen Sreedharan, Masahisa Katsuno, Koji Yamanaka

Corresponding author: Koji Yamanaka, yamanaka.koji.p4@f.mail.nagoya-u.ac.jp

Table of Content

Appendix Figure S1. Immunofluorescence using the FlexAble technology shows co-localization of monomeric TDP-43 and p-TBK1.

Appendix Figure S2. Colocalization of monomeric TDP-43 and p-TBK1 in spinal motor neurons from ALS patients (related to Fig. 1C).

Appendix Figure S3. Colocalization of monomeric TDP-43 and TBK1 in live cells (related to Fig. 1D).

Appendix Figure S4. TBK1 overexpression does not affect mRNA levels of transfected TDP-43.

Appendix Figure S5. The reduction of monomeric TDP-43 by TBK1 overexpression is blocked by proteasome inhibition.

Appendix Figure S6. Humoral factors induced by TBK1 ameliorate TDP-43 pathology in cells (related to Fig. 2).

Appendix Figure S7. Recombinant IFNβ treatment reduces HA-tagged TDP-43^{6M} in cells.

Appendix Figure S8. Recombinant IFNβ treatment reduces wild-type and mutant TDP-43 in cells.

Appendix Figure S9. IFNAR1 is expressed ubiquitously in human cells.

Appendix Figure S10. Spinal motor neurons and microglia express IFNAR1 at the protein level.

Appendix Figure S11. IFNAR1 expression was lower in TDP-43 pathology-positive neurons than in pathology-negative neurons in ALS patients (related to Figs. 5C–F).

Appendix Figure S12. IFNβ induces expression of immunoproteasome subunits in iPSC-LMNs and mouse primary spinal neurons (related to Figs. 6F–H).

Appendix Figure S13. The phosphorylation of STING at Ser365 is observed primarily in neurons of *SOD1*^{G93A} mice at disease onset.

Appendix Table S1. Clinical information of patients with ALS or other diseases

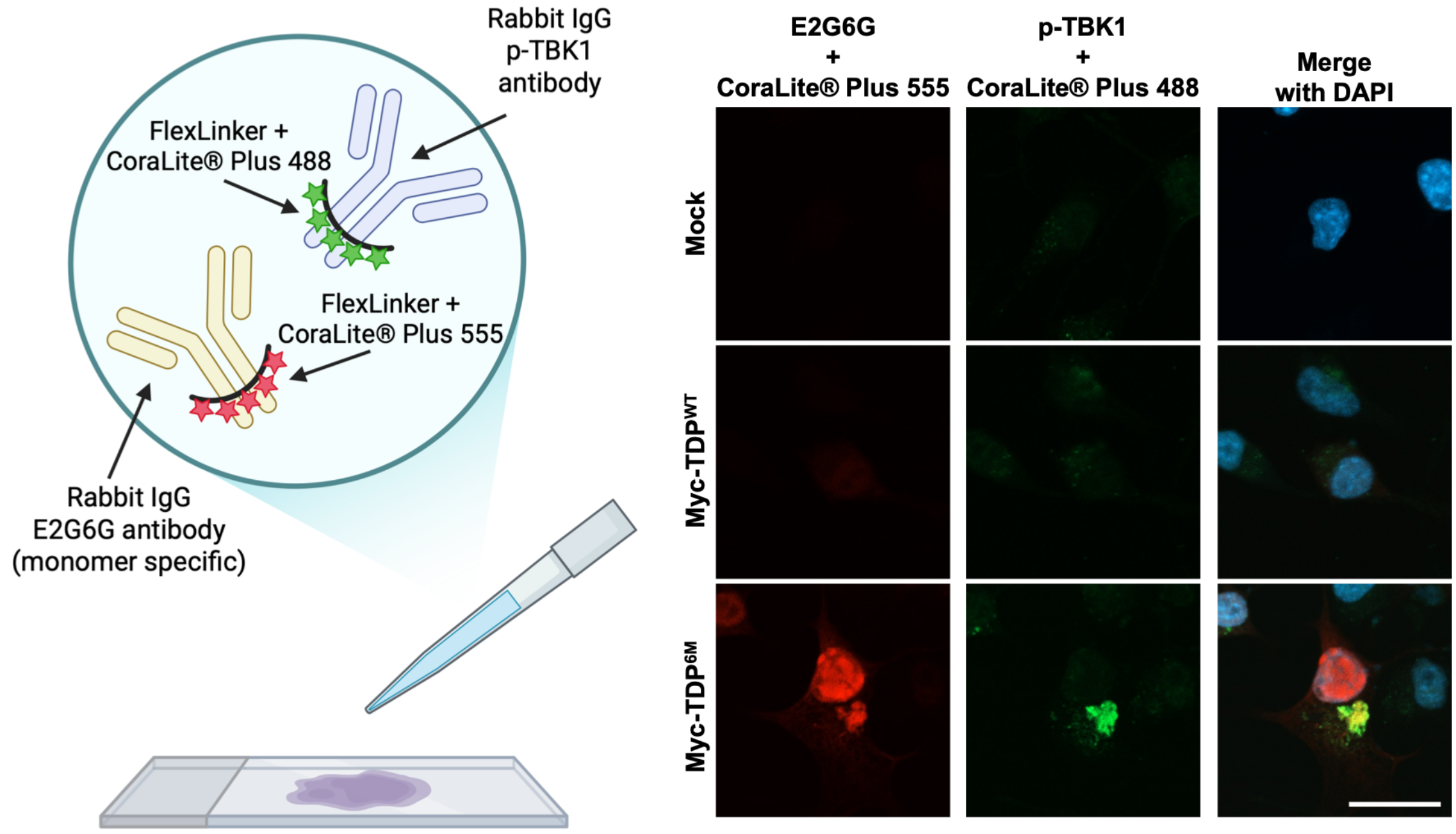

Appendix Figure S1. Immunofluorescence using the FlexAble technology shows co-localization of monomeric TDP-43 and p-TBK1.

Neuro2a cells were transfected with either Mock or TDP-43 expression vectors (WT or 6M) and analyzed by immunocytochemistry. Antibodies against monomeric TDP-43 (E2G6G) and p-TBK1 were fluorescently labeled using the FlexAble technology prior to immunostaining. Scale bar, 20 µm.

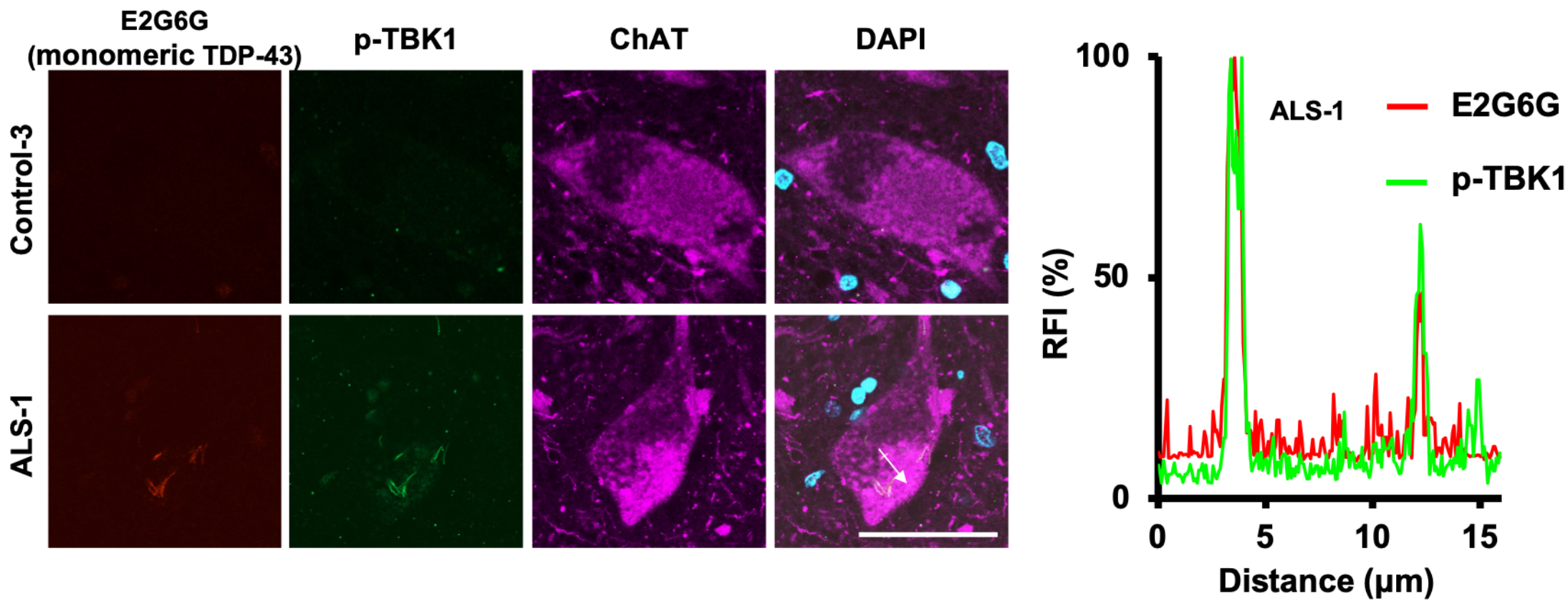

Appendix Figure S2. Colocalization of monomeric TDP-43 and p-TBK1 in spinal motor neurons from ALS patients (related to Fig. 1C).

Representative images show immunostaining of p-TBK1 and monomeric TDP-43 (E2G6G), together with a spinal motor neuron marker ChAT, in spinal cords from subjects different from those shown in Fig. 1C. Relative fluorescence intensity (RFI) of each channel at points along the white arrows is shown. Scale bar, 50 μm.

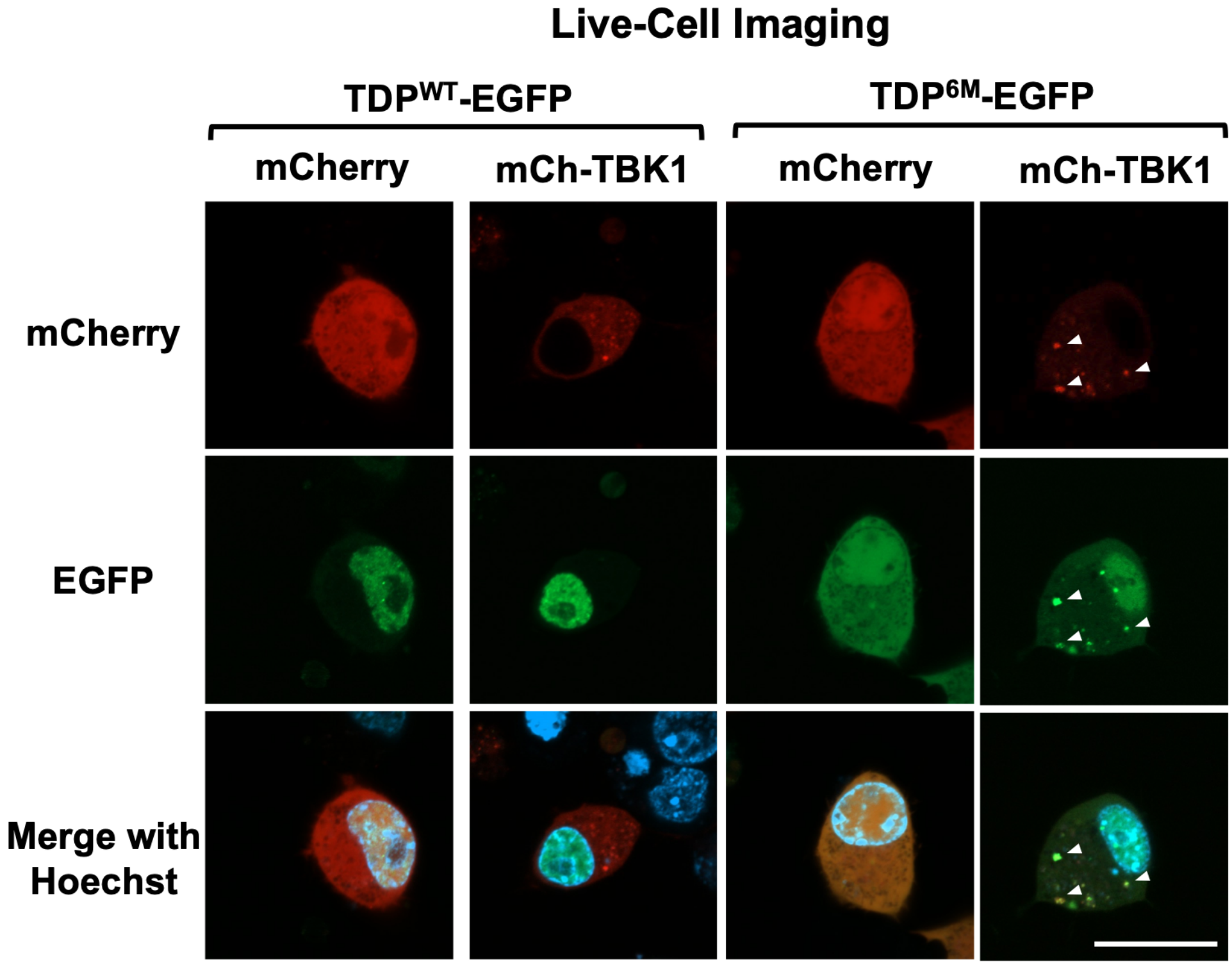

Appendix Figure S3. Colocalization of monomeric TDP-43 and TBK1 in live cells (related to Fig. 1D).

Neuro2a cells were transiently co-transfected with the indicated expression vectors and analyzed by live-cell imaging. White arrowheads indicate colocalization of TDP-43^{6M} and TBK1. Scale bar, 20 µm.

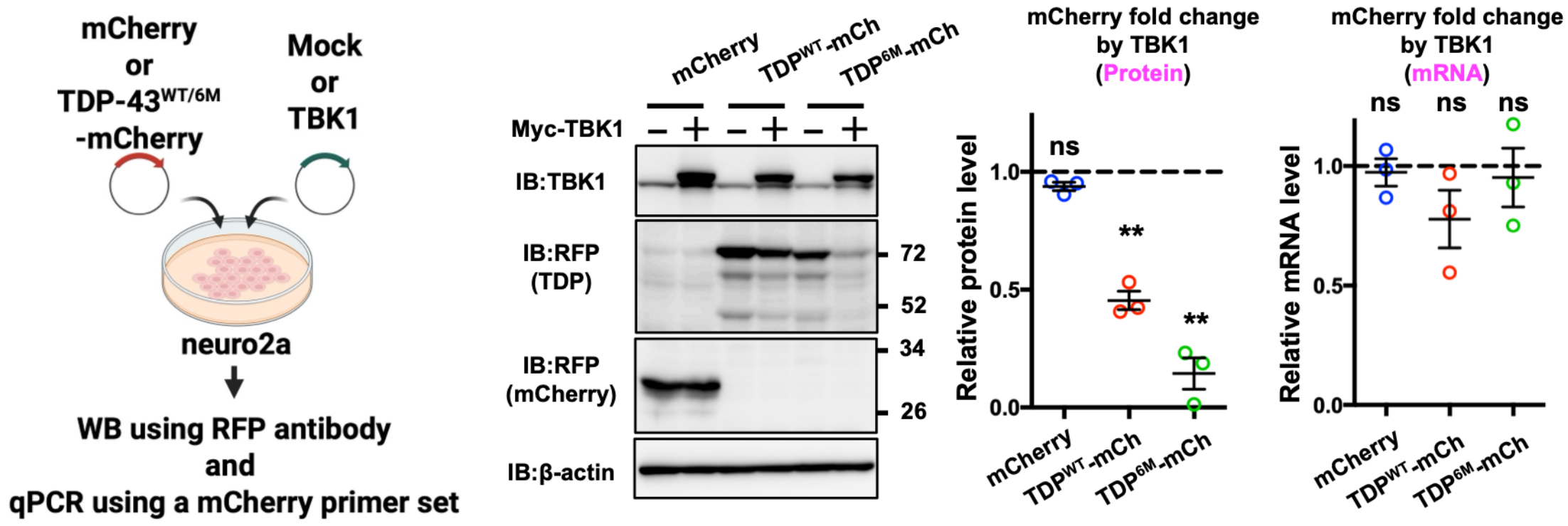

Appendix Figure S4. TBK1 overexpression does not affect mRNA levels of transfected TDP-43.

Neuro2a cells were co-transfected with the indicated plasmids and analyzed by immunoblotting and qPCR. Representative immunoblotting data along with the relative protein fold changes of mCherry and TDP-43$^{WT/6M}$-mCherry by TBK1 overexpression from three independent experiments are shown. Relative mRNA fold changes were determined by qPCR using RNA extracted from the same samples. All data are expressed as means ± SEM. Unpaired t-test with Welch's correction. ns, not significant; **$p < 0.01$.

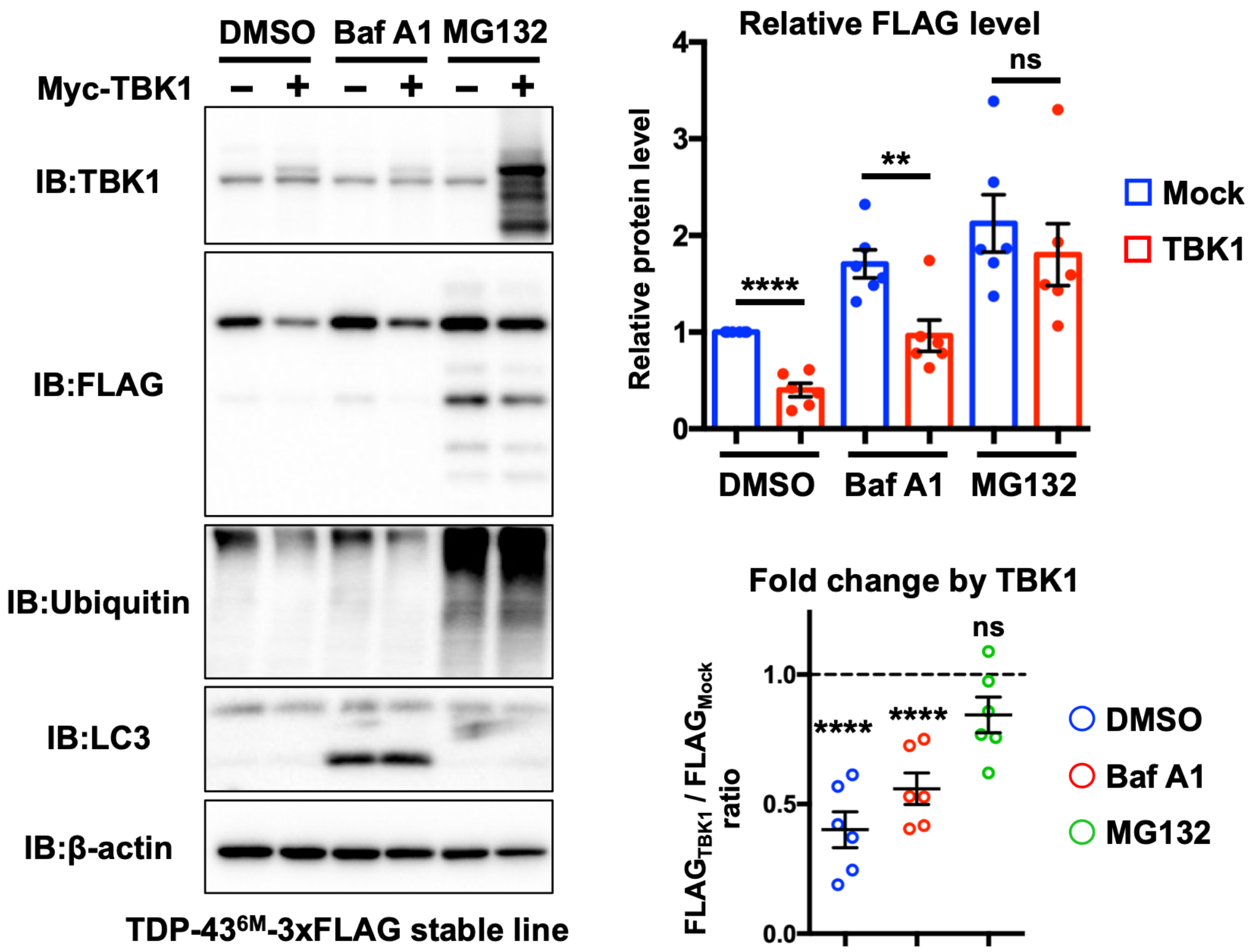

Appendix Figure S5. The reduction of monomeric TDP-43 by TBK1 overexpression is blocked by proteasome inhibition.

NSC-34 cells stably expressing Dox-inducible TDP-43^{6M}-3×FLAG were transfected with either Mock or TBK1 expression vectors and treated with DMSO, 100 nM bafilomycin A1 (Baf A1), or 10 μM MG132. Representative immunoblotting data with relative FLAG levels and the ratio of FLAG levels in TBK1-overexpressing cells relative to Mock are shown. n = 6, biologically independent experiments. All data are expressed as means ± SEM. Unpaired t-test with Welch's correction (lower graph) and one-way ANOVA followed by Holm–Sidak's multiple comparisons test (upper graph). ns, not significant; **p < 0.01, ****p < 0.0001.

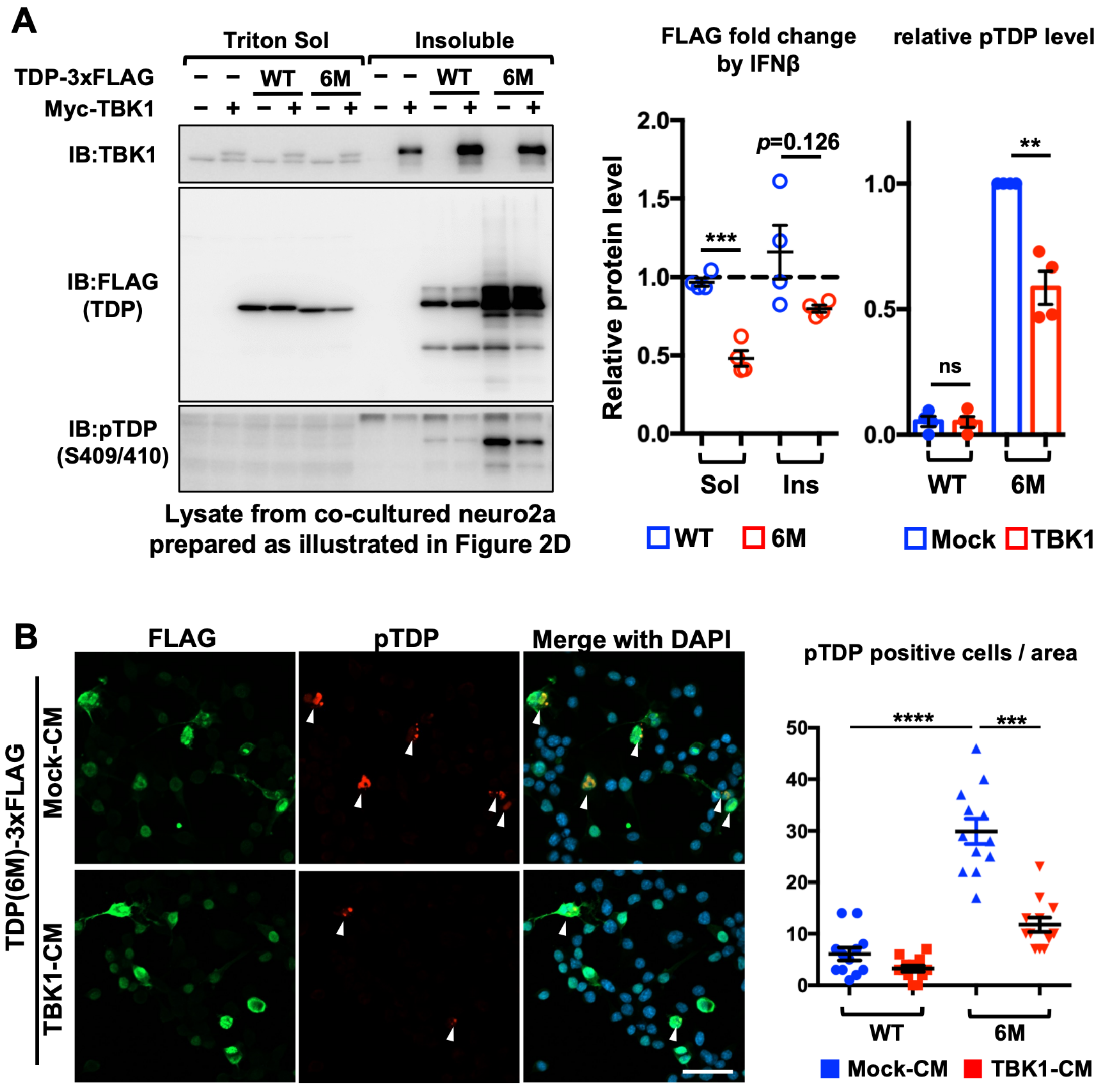

Appendix Figure S6. Humoral factors induced by TBK1 ameliorate TDP-43 pathology in cells (related to Fig. 2).

A. Neuro2a cells were prepared as illustrated in Fig. 2D, and analyzed by immunoblotting. Representative immunoblotting images, along with the relative fold changes of FLAG by IFNβ and the relative pTDP levels are shown. $n = 4$, biologically independent experiments. **B.** Neuro2a cells were prepared as illustrated in Fig. 2F, and analyzed by immunocytochemistry. Representative images of the TDP-43^{6M} samples are shown. The number of pTDP-positive cells per 0.5 mm^2 area is plotted, based on 12 areas from 3 independent biological replicates. Scale bar, 50 µm. All data are presented as means ± SEM. Unpaired t-test with Welch's correction [A (left graph)] and one-way ANOVA followed by Holm–Sidak's multiple comparisons test [A (right graph), and B]. ns, not significant; **p < 0.01, ***p < 0.001, ****p < 0.0001.

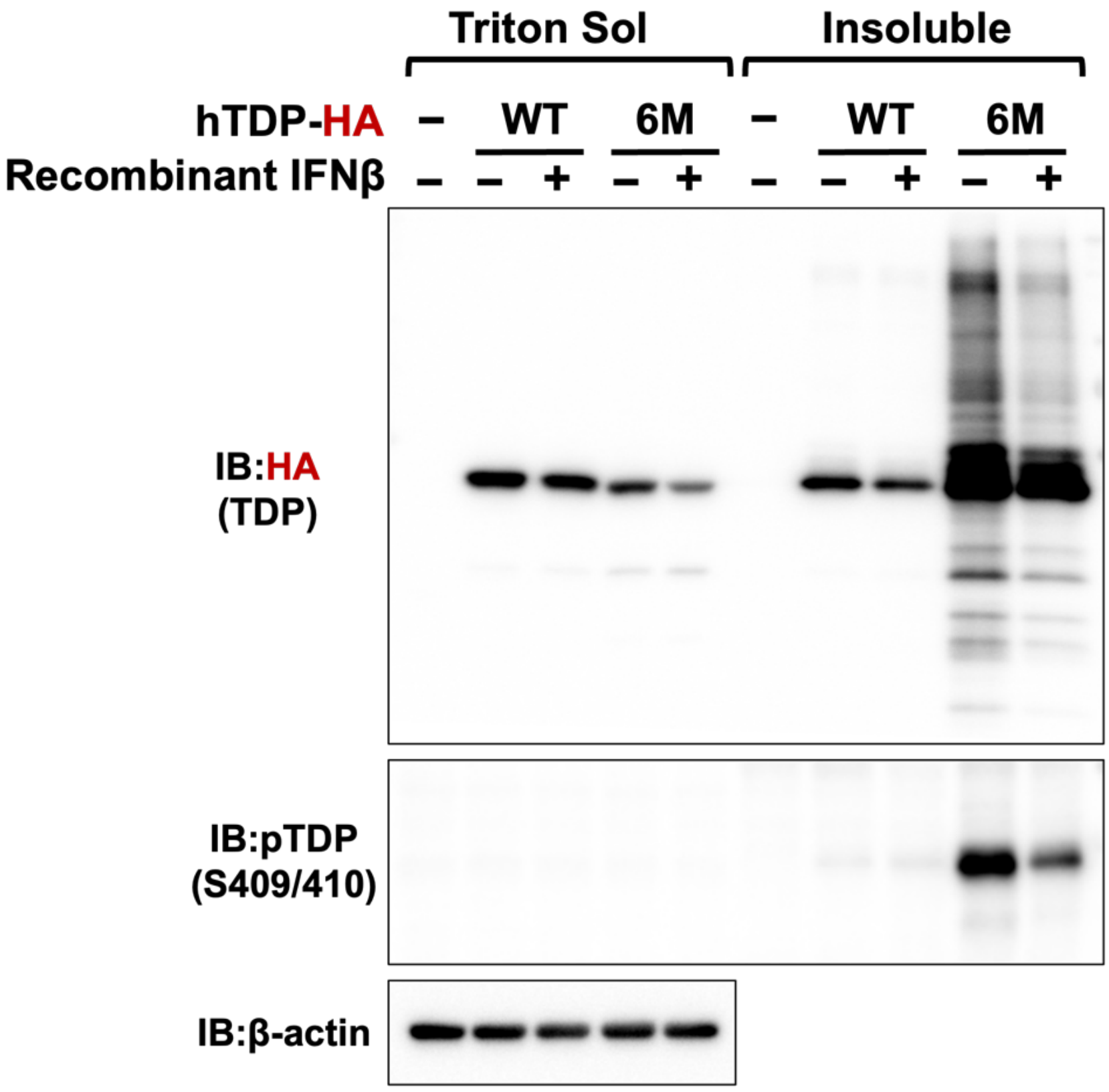

Appendix Figure S7. Recombinant IFNβ treatment reduces HA-tagged TDP-43^{6M} in cells. Neuro2a cells transiently transfected with the indicated HA-tagged TDP-43 vectors were treated with either PBS or 10 ng/mL mouse IFNβ for 42 h. The cells were then fractionated into soluble and insoluble fractions using 1% Triton X-100 and analyzed by immunoblotting.

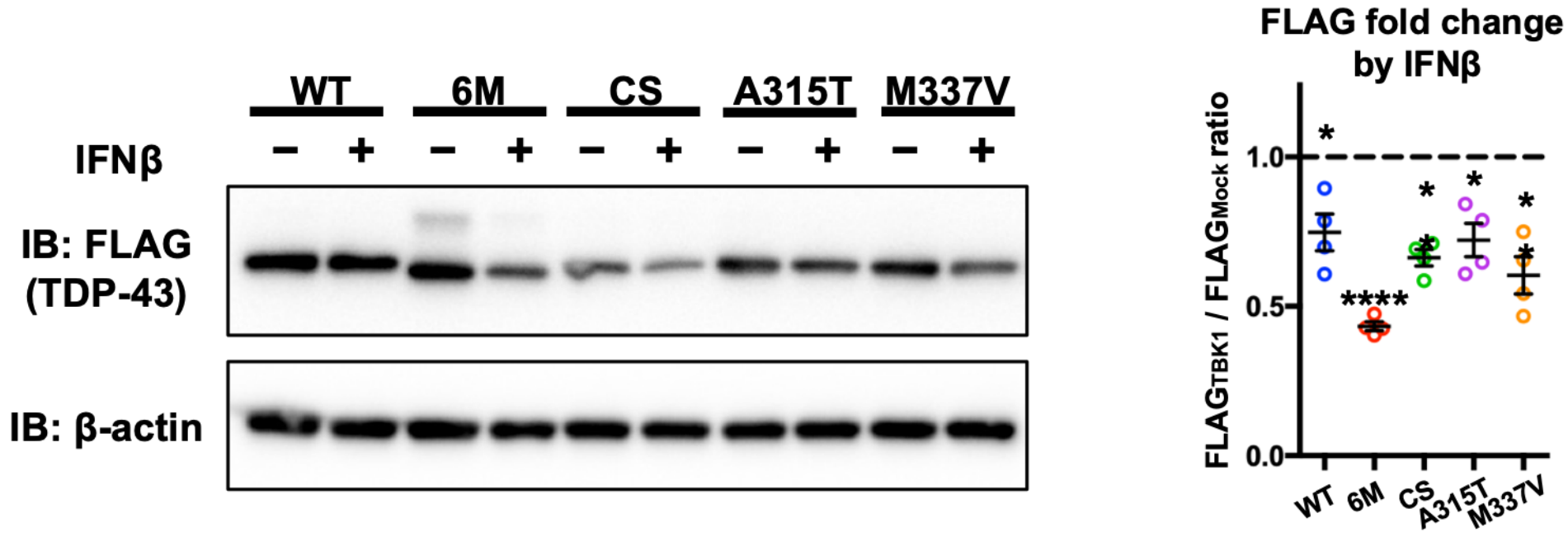

Appendix Figure S8. Recombinant IFNβ treatment reduces wild-type and mutant TDP-43 in cells.

Neuro2a cells transiently transfected with expression vectors of the indicated TDP-43 mutants were treated with either PBS or 10 ng/mL mouse IFNβ for 42 h. Representative immunoblotting images with quantified FLAG fold changes are shown. *n* = 4, biologically independent experiments. All data are presented as means ± SEM. Unpaired t-test with Welch's correction. $*p < 0.05$, $****p < 0.0001$.

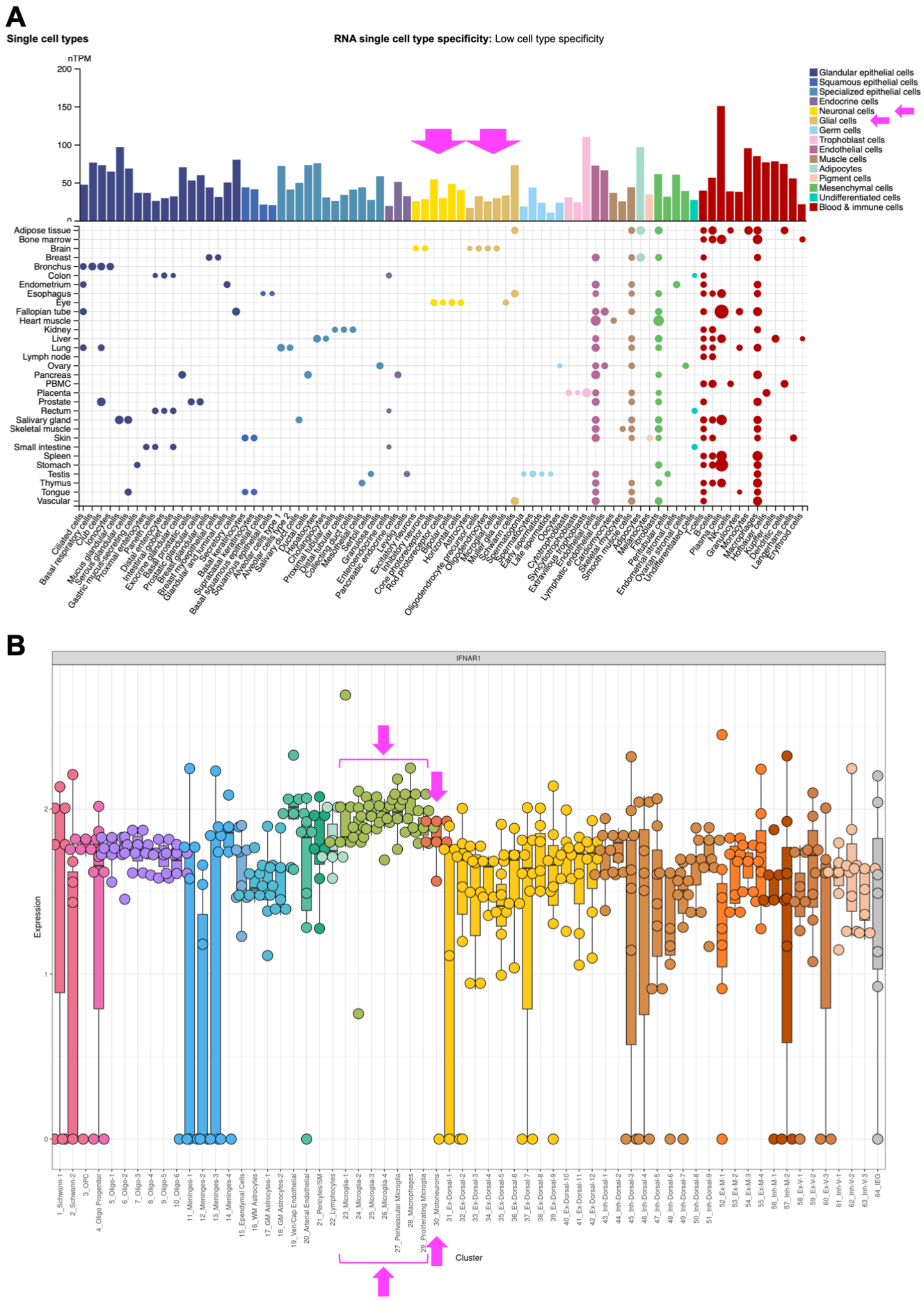

Appendix Figure S9. ***IFNAR1*** **is expressed ubiquitously in human cells.**

A. Relative mRNA expression levels of IFNAR1 in various human cell types obtained from the Human Protein Atlas (http://www.proteinatlas.org/celltype) (Karlsson *et al*, 2021) are shown. Magenta arrows indicate neuronal and glial cells. **B**. Relative mRNA expression levels of IFNAR1 in various human cell types obtained from a human lumbar spinal cord single-nucleus RNA sequencing dataset (Yadav *et al*, Neuron, 2023) are shown. The box plot was generated by a publicly available and web interface (https://vmenon.shinyapps.io/humanspinalcord/). Magenta arrows indicate motor neuron and microglia clusters.

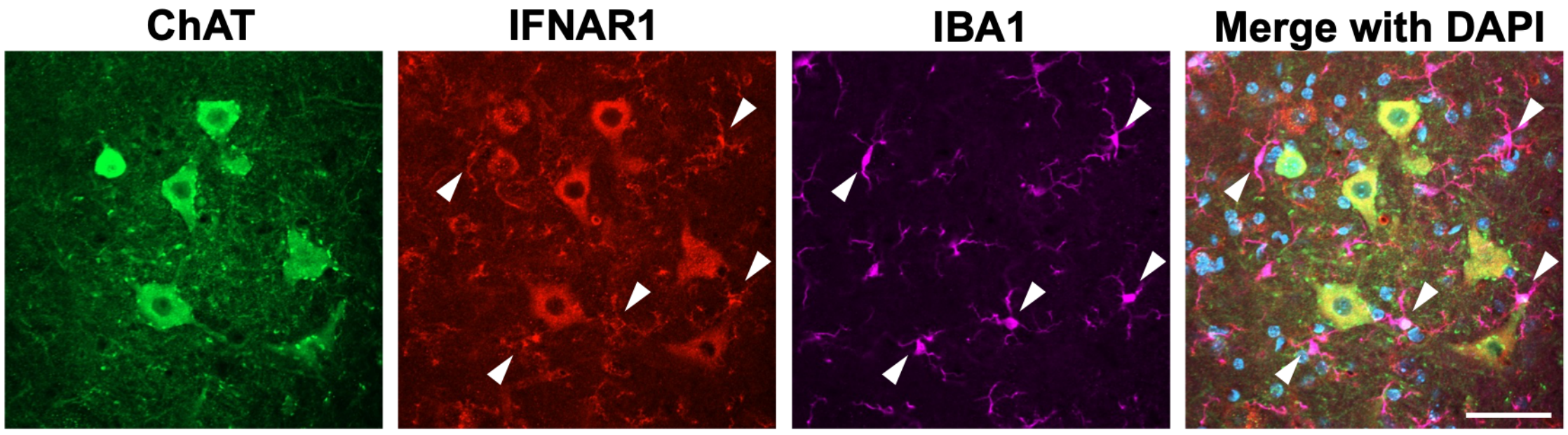

Appendix Figure S10. Spinal motor neurons and microglia express IFNAR1 at the protein level.

Representative images show IFNAR1 immunostaining with a spinal motor neuron marker ChAT and a microglial marker IBA1 in lumbar spinal cords from WT mice. The arrowheads indicate IFNAR1/IBA1 double-positive cells. Scale bar, 50 μm.

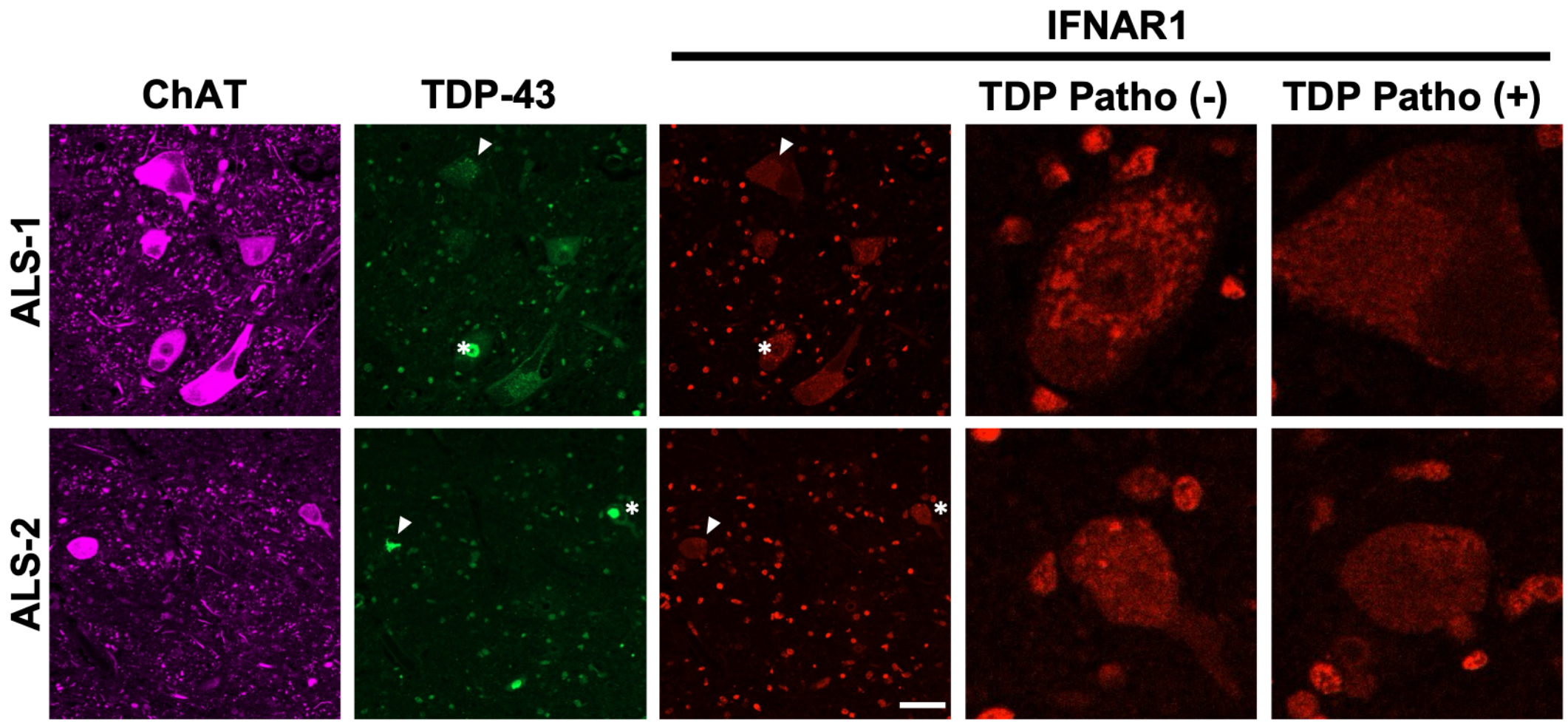

Appendix Figure S11. IFNAR1 expression was lower in TDP-43 pathology-positive neurons than in pathology-negative neurons in ALS patients (related to Figs. 5C–F).

Representative images show IFNAR1 immunostaining with ChAT and TDP-43 in spinal cords from ALS patients (D). The asterisk and arrowheads indicate TDP-43 pathology-negative and pathology-positive motor neurons, respectively. Scale bar, 50 µm.

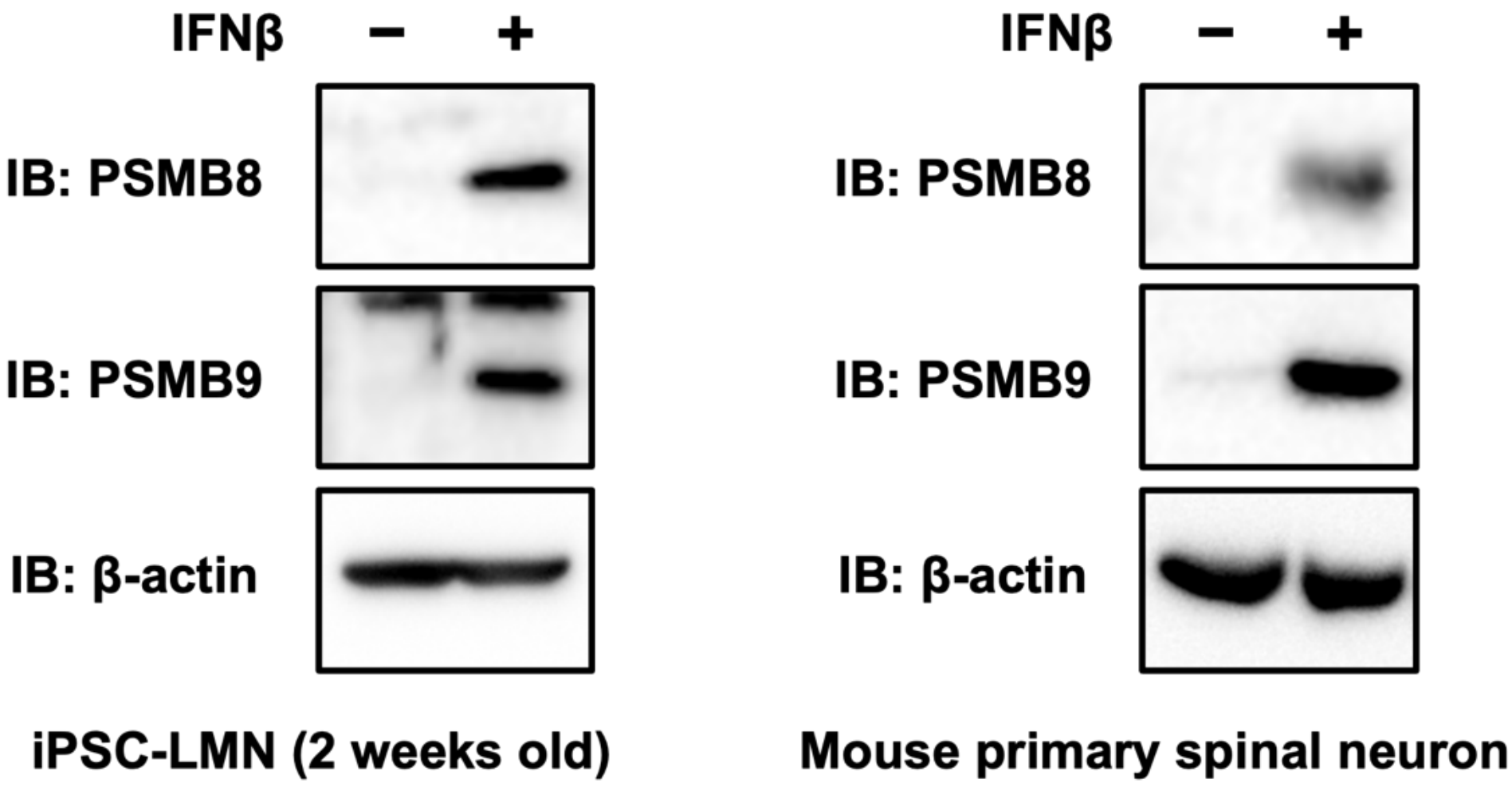

Figure S12. IFNβ induces expression of immunoproteasome subunits in iPSC-LMNs and mouse primary spinal neurons (related to Figs. 6F–H).

iPSC-derived LMNs at 2 weeks and mouse primary spinal neurons were treated with either PBS or IFNβ and analyzed by immunoblotting.

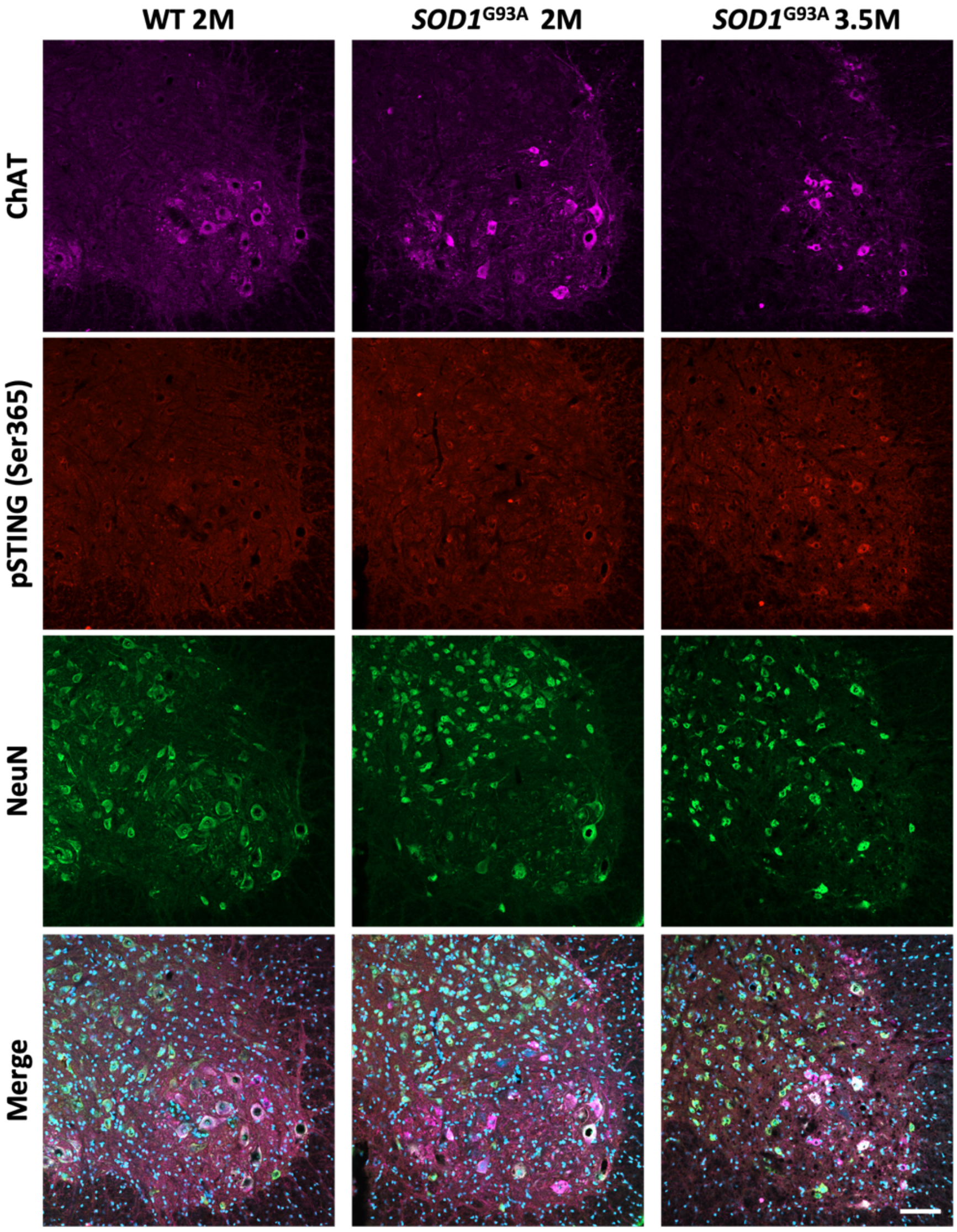

Appendix Figure S13. The phosphorylation of STING at Ser365 is observed primarily in neurons of *SOD1*^{G93A} mice at disease onset.

Representative images of phosphorylated STING (Ser365) immunostaining with NeuN and ChAT in the lumbar spinal cords of the indicated mice. Scale bar, 100 µm. M, months old.

Case	Clinical diagnosis	Age	Sex	Disese duration	Region
Control-1	MSA	75	male	4 years	CSC (C6)
Control-2	CVD	74	male	-	CSC (C7)
Control-3	PD	82	female	4 years	LSC (L2)
Control-4	FAP	71	male	-	LSC (L2)
ALS-1	ALS	68	male	6 months	LSC (L4)
ALS-2	ALS	68	female	6 months	LSC (L4-5)
ALS-3	ALS	85	male	1 year	CSC (C6)
ALS-4	ALS	68	male	2 years	LSC (L4)
ALS-5	ALS	69	male	2 years	LSC (L5)
ALS-6	ALS	72	female	7 years	CSC (C5-6)

ALS, amyotrophic lateral sclerosis; MSA, Multiple system atrophy; CVD, cerebrovascular disease; PD, Parkinson's disease; FAP, familial amyloidotic polyneuropathy; CSC, cervical spinal cord; LSC; lumbar spinal cord

Appendix Table S1. Clinical information of patients with ALS or other diseases.
