# Supplementary material for: TBK1 eliminates aggregation-prone monomeric TDP-43 through an IFNβ-immunoproteasome pathway in ALS models
Source: EMBO Rep. 2026 Oct 5;27(21):6251–76. doi: 10.1038/s44319-026-00953-8 (PMC13743774; doi:10.1038/s44319-026-00953-8)
Supplement: Supplementary file 2 [file 44319_2026_953_MOESM2_ESM.pdf]

EMBO reports

TBK1 eliminates aggregation-prone monomeric TDP-43 through an IFNβ-immunoproteasome pathway in ALS models

Shohei Sakai, Kotaro Oiwa, Yohei Iguchi, Izzet Mehmet Akcay, Seiji Watanabe, Okiru Komine, Noriko Nagata, Mai Horiuchi, Jemeen Sreedharan, Masahisa Katsuno, and Koji Yamanaka

Corresponding author(s): Koji Yamanaka (yamanaka.koji.p4@f.mail.nagoya-u.ac.jp)

Review Timeline:

Submission Date:	6th Oct 25
Editorial Decision:	4th Nov 25
Revision Received:	6th Aug 26
Editorial Decision:	25th Aug 26
Revision Received:	7th Sep 26
Accepted:	18th Sep 26

Editor: Esther Schnapp

Transaction Report:

(Note: With the exception of the correction of typographical or spelling errors that could be a source of ambiguity, letters and reports are not edited. Depending on transfer agreements, referee reports obtained elsewhere may or may not be included in this compilation. Referee reports are anonymous unless the Referee chooses to sign their reports.)

1st Editorial Decision **4th Nov 2025**

Dear Prof. Yamanaka,

Thank you for the submission of your manuscript to EMBO reports. We have now received the full set of referee reports that is pasted below.

As you will see, all referees acknowledge that the findings are interesting and novel, which is great. The referees also have several suggestions for how the study could be improved, and I think all suggestions are good and should be addressed, except may be point 3 by referee 3, which does not seem to be a crucial point, given that you already tested several cell lines. Naturally, if you have such data, please add them to this study. Please let me know if you have any comments or concerns regarding the set of referee reports and we can discuss the exact revision requirements further, also in a video chat, if you like.

I would thus like to invite you to revise your manuscript with the understanding that the referee concerns must be fully addressed and their suggestions taken on board. Please address all referee concerns in a complete point-by-point response. Acceptance of the manuscript will depend on a positive outcome of a second round of review. It is EMBO reports policy to allow a single round of major revision only and acceptance or rejection of the manuscript will therefore depend on the completeness of your responses included in the next, final version of the manuscript.

We realize that it is difficult to revise to a specific deadline. In the interest of protecting the conceptual advance provided by the work, we recommend a revision within 3 months (4th Feb 2026). Please discuss the revision progress ahead of this time with the editor if you require more time to complete the revisions.

IMPORTANT NOTE: we perform an initial quality control of all revised manuscripts before re-review. Your manuscript will FAIL this control and the handling will be DELAYED if the following APPLIES:
1) A data availability section providing access to data deposited in public databases is missing. If you have not deposited any data, please add a sentence to the data availability section that explains that.
2) Your manuscript contains statistics and error bars based on n=2. Please use scatter blots in these cases. No statistics should be calculated if n=2.

When submitting your revised manuscript, please carefully review the instructions that follow below. Failure to include requested items will delay the evaluation of your revision.

1) a .docx formatted version of the manuscript text (including legends for main figures, EV figures and tables). Please make sure that the changes are highlighted to be clearly visible.

2) individual production quality figure files as .eps, .tif, .jpg (one file per figure). See https://wol-prod-cdn.literatumonline.com/pb-assets/embo-site/EMBOPress_Figure_Guidelines_061115-1561436025777.pdf for more info on how to prepare your figures.

3) We replaced Supplementary Information with Expanded View (EV) Figures and Tables that are collapsible/expandable online. A maximum of 5 EV Figures can be typeset. EV Figures should be cited as 'Figure EV1, Figure EV2" etc... in the text and their respective legends should be included in the main text after the legends of regular figures.

- For the figures that you do NOT wish to display as Expanded View figures, they should be bundled together with their legends in a single PDF file called *Appendix*, which should start with a short Table of Content. Appendix figures should be referred to in the main text as: "Appendix Figure S1, Appendix Figure S2" etc. See detailed instructions regarding expanded view here: <https://www.embopress.org/page/journal/14693178/authorguide#expandedview>

- Additional Tables/Datasets should be labeled and referred to as Table EV1, Dataset EV1, etc. Legends have to be provided in a separate tab in case of .xls files. Alternatively, the legend can be supplied as a separate text file (README) and zipped together with the Table/Dataset file.

4) a .docx formatted letter INCLUDING the reviewers' reports and your detailed point-by-point responses to their comments. As part of the EMBO Press transparent editorial process, the point-by-point response is part of the Review Process File (RPF), which will be published alongside your paper.

5) a complete author checklist, which you can download from our author guidelines <https://www.embopress.org/page/journal/14693178/authorguide>. Please insert information in the checklist that is also reflected in the manuscript. The completed author checklist will also be part of the RPF.

6) Please note that all corresponding authors are required to supply an ORCID ID for their name upon submission of a revised manuscript (<https://orcid.org/>). Please find instructions on how to link your ORCID ID to your account in our manuscript tracking system in our Author guidelines
<https://www.embopress.org/page/journal/14693178/authorguide#authorshipguidelines>

7) Before submitting your revision, primary datasets produced in this study need to be deposited in an appropriate public database (see https://www.embopress.org/page/journal/14693178/authorguide#datadeposition). Please remember to provide a reviewer password if the datasets are not yet public. The accession numbers and database should be listed in a formal "Data Availability" section placed after Materials & Method (see also https://www.embopress.org/page/journal/14693178/authorguide#datadeposition). Please note that the Data Availability Section is restricted to new primary data that are part of this study. * Note - All links should resolve to a page where the data can be accessed. *
If your study has not produced novel datasets, please mention this fact in the Data Availability Section.

8) At EMBO Press we ask authors to provide source data for the main manuscript figures. You will receive a separate email with instructions for providing source data with your revised manuscript, including how to upload and organize the files.

9) Our journal also encourages inclusion of *data citations in the reference list* to directly cite datasets that were re-used and obtained from public databases. Data citations in the article text are distinct from normal bibliographical citations and should directly link to the database records from which the data can be accessed. In the main text, data citations are formatted as follows: "Data ref: Smith et al, 2001" or "Data ref: NCBI Sequence Read Archive PRJNA342805, 2017". In the Reference list, data citations must be labeled with "[DATASET]". A data reference must provide the database name, accession number/identifiers and a resolvable link to the landing page from which the data can be accessed at the end of the reference. Further instructions are available at https://www.embopress.org/page/journal/14693178/authorguide#referencesformat

10) Regarding data quantification (see Figure Legends: https://www.embopress.org/page/journal/14693178/authorguide#figureformat)

The following points must be specified in each figure legend:

- the name of the statistical test used to generate error bars and P values,

- the number (n) of independent experiments (please specify technical or biological replicates) underlying each data point,

- the nature of the bars and error bars (s.d., s.e.m.),

- If the data are obtained from n Program fragment delivered error ``Can't locate object method "less" via package "than" (perhaps you forgot to load "than"?) at //ejpvfs23/sites23b/embor_www/letters/embor_decision_revise_and_review.txt line 56.' 2, use scatter blots showing the individual data points.

Discussion of statistical methodology can be reported in the materials and methods section, but figure legends should contain a basic description of n, P and the test applied.

- Please also include scale bars in all microscopy images.

11) The journal requires a statement specifying whether or not authors have competing interests (defined as all potential or actual interests that could be perceived to influence the presentation or interpretation of an article). In case of competing interests, this must be specified in your disclosure statement. Further information: https://www.embopress.org/competing-interests

12) All Materials and Methods need to be described in the main text using our 'Structured Methods' format, which is required for all research articles. According to this format, the Methods section includes a separate Reagents and Tools Table file (listing key reagents, experimental models, software and relevant equipment and including their sources and relevant identifiers) and a Methods and Protocols section describing the methods using a step-by-step protocol format. The aim is to facilitate adoption of the methodologies across labs. More information on how to adhere to this format as well as a downloadable template (.docx) for the Reagents and Tools Table can be found in our author guidelines: https://www.embopress.org/page/journal/14693178/authorguide#structuredmethods.

An example of a Method paper with Structured Methods can be found here: https://www.embopress.org/doi/full/10.1038/s44320-024-00037-6#sec-4

We would also welcome the submission of cover suggestions, or motifs to be used by our Graphics Illustrator in designing a cover.

As part of the EMBO publication's Transparent Editorial Process, EMBO reports publishes online a Review Process File (RPF) to accompany accepted manuscripts. This File will be published in conjunction with your paper and will include the referee reports, your point-by-point response and all pertinent correspondence relating to the manuscript.

You are able to opt out of this by letting the editorial office know (emboreports@embo.org). If you do opt out, the Review Process File link will point to the following statement: "No Review Process File is available with this article, as the authors have chosen not to make the review process public in this case."

I look forward to seeing a revised form of your manuscript when it is ready.

Best regards,
Esther

Esther Schnapp, PhD
Senior Editor
EMBO reports

Referee #1:

This paper focuses on the dysfunction of TBK-1 in ALS pathogenesis and discovers a novel pathway: the induction of the immunoproteasome via the STING-IRF3-IFNβ axis for the degradation of pathological TDP-43. It is commendable that this study clearly demonstrates, for the first time, the involvement of the immunoproteasome in the degradation of ALS-related proteins. Interestingly, this pathway was shown to be involved not only in TDP-43 pathology but also in ALS pathology caused by mutant SOD1. More surprisingly, increased TBK-1 expression drives proteolysis through a non-cell-autonomous mechanism mediated by IFNβ.
These are entirely novel findings. The authors propose a new mechanism for protein quality control in ALS and present a remarkable achievement by elegantly linking two seemingly opposing biological processes: neuroinflammation and protein quality control. However, the decisive role of monomeric TDP-43 in ALS pathogenesis remains controversial, and there are several hurdles that must be overcome before these findings can be definitively established as a central mechanism in ALS patients.
Major Points
Q1. The degradation of TDP-43 by TBK-1 is mediated by IFNβ in a non-cell-autonomous manner, as evidenced by the effects of the cytokine IFNβ and conditioned medium. If this is the case, what is the upstream trigger that initiates TBK-1 expression in response to misfolded TDP-43? Please clarify the sequence of events in this pathway and provide a mechanistic rationale for how misfolded protein accumulation leads to increased TBK-1 expression.
Q2. The authors demonstrate TBK-1-mediated degradation only with monomeric TDP-43. Does this degradation mechanism also apply to FALS-associated TDP-43 mutations (e.g., A315T, M337V)? Given the ongoing debate about whether monomers, oligomers, or aggregates are the primary pathogenic species in ALS, conclusions about TDP-43 proteinopathy based solely on monomeric TDP-43 may be premature and could be misleading.
Q3. Most of the foundational data were generated in N2A cells. Data demonstrating IFNβ induction by TBK-1 overexpression in NSC34 cells, a motor neuron model, are not presented. While TBK-1's direct role and STING/IRF3 phosphorylation have been demonstrated in other cell types, was this pathway confirmed similarly in NSC34 cells? This validation is critical given the motor neuron-specific nature of ALS pathology.
Q4. The immunohistochemistry data are not fully convincing and require clarification on several points:
Is immunoproteasome/IFNβ expression reduced specifically in dying motor neurons, or is the reduction uniform across all motor neurons?
Conversely, do surviving neurons without TDP-43 mislocalization show higher expression?
Is there a difference in IFNβ expression patterns between neurons with round inclusions versus those with skein-like or diffuse dot patterns of TDP-43?
Was IFNβ expression examined at the transcriptional level via qRT-PCR or similar methods?
Please provide higher magnification images showing both TDP-43-negative and TDP-43-positive neurons within the same low-magnification field to allow direct comparison.
Q5. Please discuss potential mechanisms by which IFNβ receptor expression becomes reduced in specific neurons during disease progression. Is this due to cell-intrinsic dysfunction (e.g., transcriptional failure, feedback inhibition) or external factors (e.g., loss of paracrine signaling, microenvironmental changes)?
Q6. Figure 6 shows aging-induced immunoproteasome activation in aged mice. Did you observe corresponding induction of TBK-1 or IFNβ during this aging process? If so, what is the relationship between aging-related and disease-related immunoproteasome activation?
Q7. The authors mention that crossing TBK-1+/– mice with SOD1 mice results in earlier disease onset compared to SOD1 mice alone, but these data are either not provided or are unclear in the manuscript. Please present Kaplan-Meier survival curves for both disease onset and survival endpoints to support this claim.
Q8. The proposal of a novel axis in which the immunoproteasome degrades ALS pathogenic proteins via IFNβ's paracrine effect is highly innovative and impressive. It would be valuable to discuss in detail how this paracrine immunoproteasome pathway differs from and potentially complements the conventional cell-intrinsic unfolded protein response (UPR). How do these two protein quality control systems interact or regulate each other in the context of ALS?
Minor Points

1. Why was TDP-43-3xFLAG used instead of TDP-43-1xFLAG? Does the single FLAG tag affect aggregation propensity or degradation kinetics differently? Please provide justification for this choice.
Overall Assessment
This study presents conceptually novel and mechanistically interesting findings that advance our understanding of protein quality control in ALS. The identification of the TBK-1/IFNβ/immunoproteasome axis represents an important contribution to the field. However, the concerns raised above, particularly regarding the reliance on monomeric TDP-43, the limited validation in motor neuron-relevant models, and the incomplete characterization of the pathway in patient tissues, need to be adequately addressed before the manuscript can be accepted for publication.

Referee #2:

The present study by Sakai from the laboratory of Koji Yamanaka studies how TANK-binding kinase 1 (TBK1) is involved in the degradation of pathological protein forms, with dominant loss-of-function mutations in TBK1 being linked to ALS/FTD.
The study builds on two previous studies from the same laboratory:
1) Watanabe et al., (PNAS, 2023, PMID: 37967220) that showed (but not as the principle result) a reduction of activated (phospohorylated) TBK1 in brain and spinal cord tissues of sporadic ALS patients, suggesting that reduced TBK1 activity could also contribute more generally to ALS.
2) Oiwa et al., (Sci Adv, 2023, PMID: 37540751) that showed a reduced dimerization of TDP43, the main ALS pathological hallmark, in brain and spinal cord tissues of sporadic ALS patients. They further show, in vitro (Neuro2a/iPSc-MN), that an engineered TDP43 protein that lacks dimerization domains (termed TDP43-6M), acts as a monomer and leads to aggregates and nuclear exclusion, suggesting that TDP43 monomerization is part of the ALS-TDP43 pathology. They also found a specific anti-TDP43 Ab that seems to recognise, in postmortem spinal cord tissues, the (pathological) cytoplasmic monomer form and less the (normal) nuclear form.

Based on these earlier results, the present study asks how TBK1 could be implicated in the degradation of pathological monomeric TDP43. The authors use an in vitro approach to overexpress their TDP43-6M in Neuro2a cells, and test what co-overexpression of TBK1 does. In the view that they use Western blot analysis, this is an acceptable in vitro approach (iPSc-MN would be not feasable for this). Both TBK1 and TDP43-6M are tagged (Myc and FLAG) for easy recognision - although not optimal, acceptable.

The authors find then a clear reduction of TDP43-6M by TBK1 overexpression (FIG.1). But very surprisingly, this seems not to be mediated by the classic autophagy-lysosomal pathway, as Bafilomycin A1 did NOT affect the TBK1-mediated reduction of TDP43-6M (FIG.1). This is a central finding of the study and from there the authors search for an alternative pathway - basically a new way how TBK1 could be implicated in degradation, especially of pathological proteins. They focus on the known immune role of TBK1 in the induction of Type I interferons (IFNb), and find convincing evidence that TBK1-induced IFNb acts paracrine on other neuronal cells to induce immunoproteasome activity, which then leads to the degradation of TDP43-6M - thus, the pathway does not go through the (neuronal) TBK1-autophagy, but a new (neuronal) TBK1-IFNb-Immunoproteasome axis (FIGs.2-3/5).

Interestingly, the authors further show that in human ALS postmortem spinal cord tissue there is a reduction of the IFNAR1 on motor neurons compared to control, and even more so in motor neurons with TDP43 pathology, thus suggesting a partial impairment of this proposed IFNb-IFNAR1 axis (FIG.4). In the spinal cord of mutant SOD1 ALS mice they further show that immunoproteasome specific subcomponents get induced with disease, while canonical proteasome subunits get reduced during disease, suggesting a compensatory response to degrade mutant proteins by this immunoproteasome axis (FIG.7). Finally, in a double-transgenic mutant SOD1 ALS mice with systemic Tbk1 reduction (heterozygous deletion), they show that this induction of immunoproteasome specific subcomponents is reduced, while they measured an increase in ubiquinated proteins, further suggesting that a TBK1-IFNb-IFNAR1-Immunoproteasome axis is implicated in protein degradation (FIG.7).

As such, this is a highly interesting study, both on a fundamental level for TBK1 functions, and with respect to pathological protein accumulations in ALS. While the study uses principally in vitro neuronal cell line systems, the effort was done to go to human postmortem tissues as well as ALS mice. To link the immune cell function of TBK1 (activation of IFNb) to an alternative protein degradation function of TBK1, not in autophagy as classically known, but instead potentially through the immunoproteasome, is very interesting, and opens up new pathways how we might be able to target degradation of pathological proteins in ALS.

However, there are some issues that need to be clarified, and expanded upon, before a possible publication can be considered.

MAJOR ISSUES :

• FIGURES 1:
1) FIG.1C: as this is a central finding (Baf A1 does NOT block the TBK1-driven TDP43-6M reduction), it would be great to have

some sort of a "positive" control for Baf A1 - to show that indeed autophagy is blocked. Also, maybe this is obvious to autophagy-experts, but why does Baf A1 not block induction of p62/LCIII by TBK1 overexpression ? Likewise, in the view of their results in Fig.5A (block of IFNb induced TDP43-6M reduction by proteasome inhibition), it would be of course very convincing to do here a proteasome inhibition, side-by-side with the autophagy inhibition.

• FIGURE 4:
2) FIG.4A/B: while the IFNAR1-immunos in the human tissues (Fig.4C/D) are quite convincing, the stains in the mouse spinal cord and especially in cerbral cortex are less so. As it is shown, IFNAR1 is mainly expressed on motor neurons. Should it not also be expressd on microglia ? What does published RNAseq data from spinal cord microglia indicate ? What does the Allen Brain Atlas show ? Or could you do a confirmatory RNAscope for IFNAR1 - just to be sure that this antibody is really specific - as this is a major point for this study. The same is also an issue for the human tissue, although I am aware that this is much more difficult due to limited material (but maybe check existing snRNAseq data for expression in MN vs microglia).

• FIGURE 5:
3) FIG.5A: The authors show very convincingly that proteasome inhibition fully blocks the IFNb induced TDP43-6M reduction (stable/inducible NSC34 lines). What would have been even more convincing, would be to show in parallel that autophagy inhibition should NOT affect this. I am aware that this has been somehow shown using transient TDP43-6M+TBK1 in Neuro2a cells in Fig.1C, but not side-by-side.

• FIGURE 6:
4) Overall, this is a bit difficult to understand. The Idea is to show that during aging in healthy mice, the IFNb downstream genes and immunoproteasome specific subunits get upregulated, in spinal cord and cerebral cortex. But how is TBK1 regulated during aging ? And the more classic autophagy genes ?
Also, while this is interesting (concerning the presented data), it would only be really powerful when we see these regulations on a cellular level. While there was an effort for PSMB8 (immunoproteasome), the immunos are not really convincing. RNAscope might be an alternative. Also, there are published RNAseq studies of (healthy/normal) aging of both motor neurons and spinal cord microglia in mice (Castro, 2023, PMID: 37154159; Castro, 2024, PMID: 37737058) - the data could be verified in there. With respect to compare aging with ALS, it would be interesting to check if the IFNAR1 is downregulated on motor neurons during normal aging in mice (using immunos, as in Fig.4A), comparable to what the authors showed for ALS MN in human postmortem tissues. Again, the aging link is interesting but stands a bit "alone" right now. Also as a comment, 12 months in mice is rather middle-aged than really "aged/old" as B6 mice live up to 30 months.

• FIGURE 7:
5) FIG.7A-E: What is very impressive here is this finding that the immunoproteasome subunits get induced with ALS disease in mutant SOD1 ALS mouse spinal cord, while the canonical proteasome subunits rather get reduced, and TBK1 reduction reduces the immunoproteasome induction. Again, also I understand the difficulty, but cell specifity would be interesting for PSMB8/9 - maybe RNAscope is possible (if fixed, floating sections are still available, this could be doable) - if not in the full Tbk1/G93A mice, then at least for the base G93A ? The paper clearly claims that this happens in MN, and the immunos in Fig.6E are not so convincing.
FIG.7F: Here the authors assess hSOD1, p62 and ubiquinated proteins in the Tbk1-het/G93A mouse spinal cord by Western, and find no effect on hSOD1 and p62, while they find increased ubiquinated proteins in the insoluble fraction - thus, they claim that autophagy is not affected but proteasome function reduced by Tbk1 reduction (supporting their idea that the TBK1-IFNb-IFNAR1-immunoproteasome axis is active in baseline ALS mice). However, we know from the Brenner 2019 study (PMID: 30635357) that early in the disease p62 is clearly increased in the same Tbk1-het/G93A mouse, while at later stages this seems to be inversed, comparable to the present study (although Brenner used immunos, while the present study used Western). Also in human TBK1-ALS-iPSc-MN, there is increased p62 detectable (PMID: 30939964). More experiments have to be done here, or the data be put more carefully into correlation with existing data. The claim that only the immunoproteasome axis is affected by Tbk1-reduction in ALS mice, seems too strong, likely both (TBK1/autophagy & TBK1/immunoproteasome) are affected, and contribute to the disease. But again, the authors finding that the TBK1/immunoproteasome axis is affected/implicated is important and novel. Also - as mutant hSOD1 is itself not affected by Tbk1-reduction (soluble/insoluble fractions are not changed), what are the proteins that are ubiquinated and thus would lead to increased toxicity - or is that not what the authors somehow would claim here ?

6) TITLE: While the title is acceptable, the addition "in ALS." seems a bit strong. The actual functional assay was only done in cell lines, and the in vivo data stems from a reduced IFNAR1 stain in ALS postmortem tissues and less immunoproteasome induction in Tbk1-het/G93A mice (plus increased amounts of insoluble ubiquinated proteins).

MINOR ISSUES :

• Fig.1A: While the explanations in the main text are easy to follow, the reading of the actual Figures with its Western Blots is less intuitive. The color blue/red is used for different things, thus not too helpful. e.g. 1A, the "FLAGtbk1/FLAGmock ratio" is not very intuitive.

• Fig.2A: typo "konckdown"

• All SFigs1-9 seem blurry ?

Referee #3:

The manuscript by Sakai et al., entitled "TBK1 eliminates aggregation-prone monomeric TDP-43 through the IFN-β-immunoproteasome pathway in ALS," investigates the role of TBK1 in ALS (amyotrophic lateral sclerosis) pathogenesis. Building on their previous work (Oiwa et al., Sci Adv 2023) characterizing an aggregation-prone TDP-43 mutant (TDP-43 6M), the authors show that TBK1 overexpression reduces, and TBK1 knockdown increases, insoluble TDP-43 6M. Although TBK1 has been thought to function primarily in the autophagy-lysosome pathway and ALS-FTD mutations appear to disrupt mitophagy (Harding et al., PNAS 2021), the authors uncover that a TBK1-mediated paracrine signaling mechanism is required to reduce TDP-43 6M aggregation. They further show that TBK1 acts through STING and IRF3 to induce secretion of IFN-β, which activates IFN-β receptors and upregulates immunoproteasome expression. This TBK1-IFN-β-immunoproteasome pathway appears to be specific to neuronal cell lines (SH-SY5Y, Neuro2a, and NSC-34) and not present in non-neuronal lines (HEK293 and HeLa). Intriguingly, immunoproteasome subunits exhibit an age-dependent increase in mouse spinal cord and cerebral cortex. Furthermore, IFNAR1, a subunit of the IFN-α/β receptor (IFNAR), is present in neurons within the spinal cord, and IFNAR1 expression appears reduced in motor neurons of ALS patients. Finally, the authors crossed SOD1-G93A mice with TBK1-heterozygous knockout mice and found that expression of canonical proteasome subunits decreased while immunoproteasome subunits increased in SOD1 mice. Moreover, the induction of immunoproteasome expression was blunted in SOD1-G93A/TBK1-heterozygous knockout mice, which exhibited higher ubiquitinated protein loads.

Overall, this is an interesting and well-executed study that reveals a potentially novel mechanism involving the TBK1-IFN-β-immunoproteasome axis in ALS pathogenesis. The manuscript is well written, with clear explanations and logical flow. The experiments are thoughtfully designed and appropriately controlled. I congratulate the authors on a job well done. The paper will be of broad interest to this journal's readership as well as to researchers in neurodegeneration. This referee is excited about these results and believes they will open a range of follow-up studies. Accordingly, I will limit my comments to the current data presented. Below are comments and suggestions to strengthen and improve the manuscript for the authors' and editor's consideration.

Major comments/suggestions:

1. Statistical analyses for human motor neurons (Figure 4E-F, Figure S7)

I appreciate that the authors provided the original data in Figure S7. If I understand correctly, the data shown in Figures 4E and 4F were pooled across subjects/patients, which inflates statistical power because motor neurons from the same subject/patient are not independent observations. The authors should consider a linear mixed-effects model or other mixed-effects approaches, or compute subject-level means and treat these as independent units (simpler but with lower power). The SuperPlots guidelines (Lord et al., J Cell Biol, 2020; doi: 10.1083/jcb.202001064) provide useful recommendations and presentation for representing subject-level data.

2. Strengthen the age-dependent increase of immunoproteasome expression (Figure 6)

The attempt to validate increased expression of immunoproteasome subunits in neurons by immunofluorescence (Figure 6E and 6F) is appreciated; however, immunofluorescence is intrinsically limited for precise quantification. Would the authors consider single-molecule RNA FISH (smFISH) combined with neuronal markers (ChAT and CTIP2)? This would corroborate the RT-qPCR data (Figure 6C-D) and further strengthen the claim.

3. Strengthen the neuronal specificity of IFN-β-mediated immunoproteasome induction (Figure 5)

The finding that IFN-β induces PSMB8 and PSMB9 in neuronal cell lines (SH-SY5Y, Neuro2a, NSC-34) but not in HEK293 or HeLa cells is compelling. To reinforce the claim in bona fide neurons, please consider testing iPSC-derived neurons (as in Oiwa et al., Sci Adv 2023) and/or primary neurons.

4. Clarification of TBK1 effects on wild-type TDP-43 (Figure 1A and 1C)

The data provided in this manuscript convincingly suggest that TDP-43 6M (aggregation prone) is degraded via the TBK1-IFN-β-immunoproteasome pathway. However, insoluble wild-type TDP-43 also appears reduced upon TBK1 overexpression (Figure 1A). Please clarify whether wild-type TDP-43 is cleared via autophagy-dependent or autophagy-independent mechanisms by performing experiments analogous to Figure 1C.

Minor points:

1. The effect of recombinant IFN-β on mutant SOD1 in Neuro2a cells (Figure S5) is less uniform than on TDP-43 (Figure 3): soluble SOD1-G93A and insoluble SOD1-G85R are reduced, whereas the other fractions are less affected. Please clarify this in the text (Lines 142-143).

2. It would be helpful to show TDP-43 levels in total lysate (before any spin) for selected experiments-especially those showing reductions in both soluble and insoluble fractions (e.g., Figures 1A, 1G, 3A)-to exclude the possibility that total TDP-43 levels are altered by TBK1 expression. This is likely not the case, but presenting total lysate controls would confirm it.

Response to the referees:

First of all, we appreciate the time and effort that the referees have dedicated to our manuscript to provide thoughtful and constructive feedback. We are pleased to resubmit the revised manuscript to *EMBO Reports*. To facilitate the review process, we have addressed each comment point-by-point.

In this revision, we have substantially revised the figures. In particular, we added a new figure describing the activation mechanism of TBK1 in response to TDP-43 as Figure 1 (with Figs. S1–S3), and renumbered the original Figures 1–5 as Figures 2–6, respectively. We have also added human iPSC-derived motor neuron data to further strengthen our conclusions. In contrast, we have removed the original Figure 6 because we were unable to address the concerns raised by the referees.

We hope that our revised manuscript, figures, and responses to the comments sufficiently address the concerns of the referees.

The newly added data are listed below:

・Figure 1A–G
・Figure 1H–K (moved and revised from the original Fig. S2A–D)
・Figure 3F and J
・Figure 4F–J
・Figure 5G
・Figure 6A, G, H, K, and M (moved from the original Fig. S8)
・Figure 7C–F
・Figure 8
・Figure S1, S2, S3, S5, S7, S8B, S9, S10, and S11
・Figure S12 (moved and revised from the original Fig. S9)

Referee #1:

This paper focuses on the dysfunction of TBK-1 in ALS pathogenesis and discovers a novel pathway: the induction of the immunoproteasome via the STING-IRF3-IFNβ axis for the degradation of pathological TDP-43. It is commendable that this study clearly demonstrates, for the first time, the involvement of the immunoproteasome in the degradation of ALS-related proteins. Interestingly, this pathway was shown to be involved not only in TDP-43 pathology but also in ALS pathology caused by mutant SOD1. More surprisingly, increased TBK-1 expression drives proteolysis through a non-cell-autonomous mechanism mediated by IFNβ.

These are entirely novel findings. The authors propose a new mechanism for protein quality control

in ALS and present a remarkable achievement by elegantly linking two seemingly opposing biological processes: neuroinflammation and protein quality control. However, the decisive role of monomeric TDP-43 in ALS pathogenesis remains controversial, and there are several hurdles that must be overcome before these findings can be definitively established as a central mechanism in ALS patients.

Response:

Thank you for your positive evaluation of our initial manuscript and for your many helpful suggestions to strengthen our study. In this revision, we have revised the figures and text based on your feedback, as listed below:

Major Points

- Q1 → Fig. 1A–G (new data), Fig. S1-S3 (new data), Fig. 1H–K (moved and revised from the original Fig. S2A–D), Fig. 3J (new data), and Fig. 8 (new schematic summary)
- Q2 → Fig. S7 (new data)
- Q3 → Fig. 3F (new data)
- Q4 → Fig. S10 (new data)
- Q5 → Fig. 5G (new data)
- Q6 → withdrawal of the original Fig. 6
- Q7 → description only
- Q8 → description only

Minor Points

- 1 → description only

Major Points

Q1. The degradation of TDP-43 by TBK-1 is mediated by IFNβ in a non-cell-autonomous manner, as evidenced by the effects of the cytokine IFNβ and conditioned medium. If this is the case, what is the upstream trigger that initiates TBK-1 expression in response to misfolded TDP-43? Please clarify the sequence of events in this pathway and provide a mechanistic rationale for how misfolded protein accumulation leads to increased TBK-1 expression.

Response:

This is a very important point. Based on your comment, we investigated whether and how monomeric TDP-43 induces TBK1 activation. Here, instead of assessing the total TBK1 expression level, we evaluated phosphorylation of TBK1, which is a well-established indicator of TBK1 activation. As shown in the new Figure 1, we demonstrated that cytoplasmic monomeric TDP-43 induces TBK1 phosphorylation via a physical interaction with TBK1 (Fig. 1A–G). To our knowledge, this is the first report demonstrating a physical interaction between TDP-43 and TBK1, and we believe that the human

data in Fig. 1C are particularly noteworthy. We also demonstrated that significance of activated TBK1 in the context of TDP-43 pathology (Fig. 1H–K: moved from the original Fig.S2). Importantly, knockdown of IFNβ in cells overexpressing monomeric TDP-43 exacerbated TDP-43 pathology (Fig. 3J), consistent with TBK1 suppression (Fig. 1H–K). Based on these results, we conclude that the cytoplasmic monomeric TDP-43 burden serves as a trigger of the TBK1-IFNβ-immunoproteasome axis. We have also incorporated this initiation event into the schematic summary in Fig. 8.

Q2. The authors demonstrate TBK-1-mediated degradation only with monomeric TDP-43. Does this degradation mechanism also apply to FALS-associated TDP-43 mutations (e.g., A315T, M337V)? Given the ongoing debate about whether monomers, oligomers, or aggregates are the primary pathogenic species in ALS, conclusions about TDP-43 proteinopathy based solely on monomeric TDP-43 may be premature and could be misleading.

Response:

According to your suggestion, we examined the effects of IFN-β treatment on another aggregation-prone mutant, C173/175S (Shodai et al., JBC, 2013), as well as the FALS-associated mutations A315T and M337V. As shown in Fig. S7, we demonstrate that this degradation mechanism also applies to these mutants. The effects on the FALS mutants were similar to those observed for wild-type TDP-43 and were milder than those observed for monomeric TDP-43. This is perhaps not surprising, as these mutants are not strongly aggregation-prone, at least in cell-based models.

Q3. Most of the foundational data were generated in N2A cells. Data demonstrating IFNβ induction by TBK-1 overexpression in NSC34 cells, a motor neuron model, are not presented. While TBK-1's direct role and STING/IRF3 phosphorylation have been demonstrated in other cell types, was this pathway confirmed similarly in NSC34 cells? This validation is critical given the motor neuron-specific nature of ALS pathology.

Response:

According to your suggestion, we overexpressed wild-type or ALS/FTD-associated mutant TBK1 in NSC-34 cells and confirmed that wild-type TBK1 induced IFN-β expression, whereas the ALS/FTD-linked kinase-dead mutants did not. These data are incorporated into the revised Fig. 3F.

Q4. The immunohistochemistry data are not fully convincing and require clarification on several points:

Is immunoproteasome/IFNβ expression reduced specifically in dying motor neurons, or is the reduction uniform across all motor neurons?

Conversely, do surviving neurons without TDP-43 mislocalization show higher expression?

Is there a difference in IFNβ expression patterns between neurons with round inclusions versus those

with skein-like or diffuse dot patterns of TDP-43?

Was IFNβ expression examined at the transcriptional level via qRT-PCR or similar methods?

Please provide higher magnification images showing both TDP-43-negative and TDP-43-positive neurons within the same low-magnification field to allow direct comparison.

Response:

We agree that this is a critical point. To investigate immunoproteasome and IFNβ expression in human postmortem tissues with TDP-43 pathology, we first needed to identify reliable antibodies against human immunoproteasome subunits and IFNβ. We screened more than ten commercially available antibodies and identified antibodies against both PSMB8 (an immunoproteasome subunit) and IFNβ that worked well with methanol-fixed samples, but not with PFA-fixed samples (please see the "figure for referees only" below).

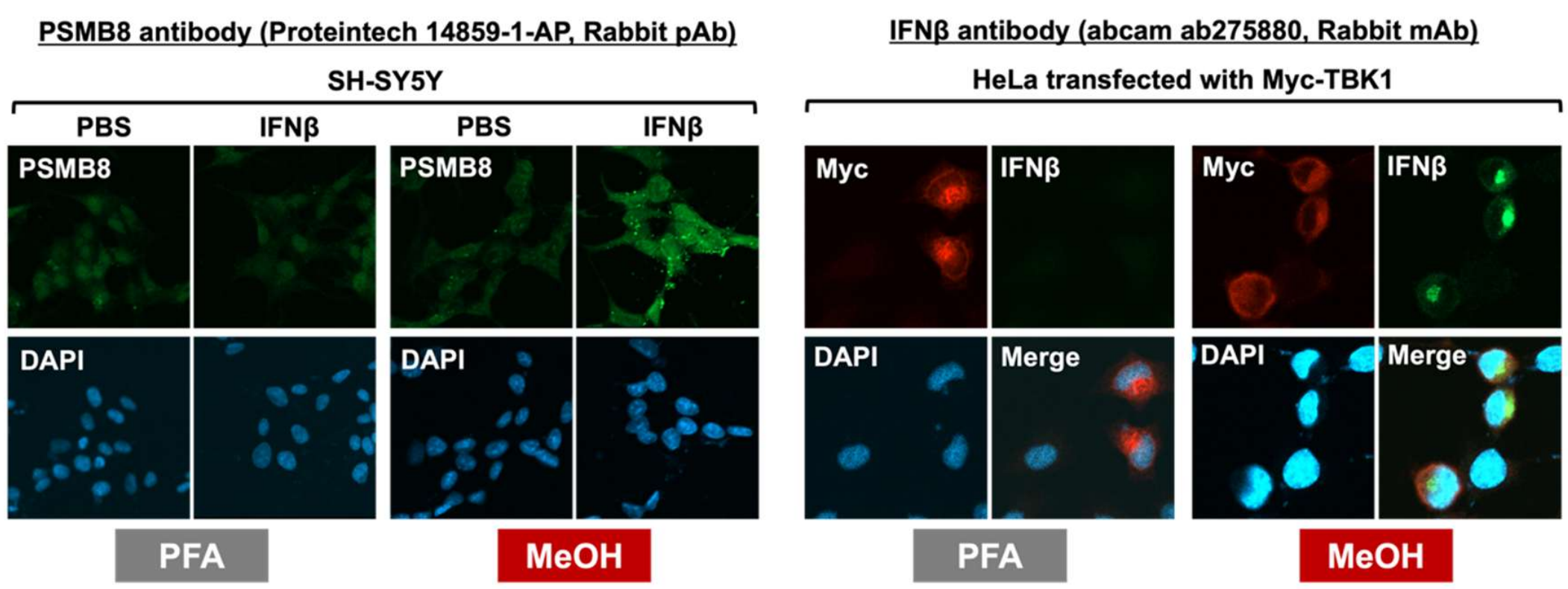

Figure for Referees only: The identified PSMB8 and IFN-β antibodies were not compatible with PFA-fixed samples.

IFNβ treatment and TBK1 transfection were performed to prepare positive controls for PSMB8 and IFNβ, respectively.

Next, we tested these antibodies in formalin-fixed, paraffin-embedded (FFPE) human tissues; however, we were unable to obtain interpretable results (please see the "figure for referees only" below), likely because these antibodies were not compatible with formalin-fixed samples, as suggested by their failure to work with PFA-fixed samples. As you pointed out, we agree that characterization of the immunoproteasome and IFNβ in patient tissues is critically important. We plan to address this point in future studies.

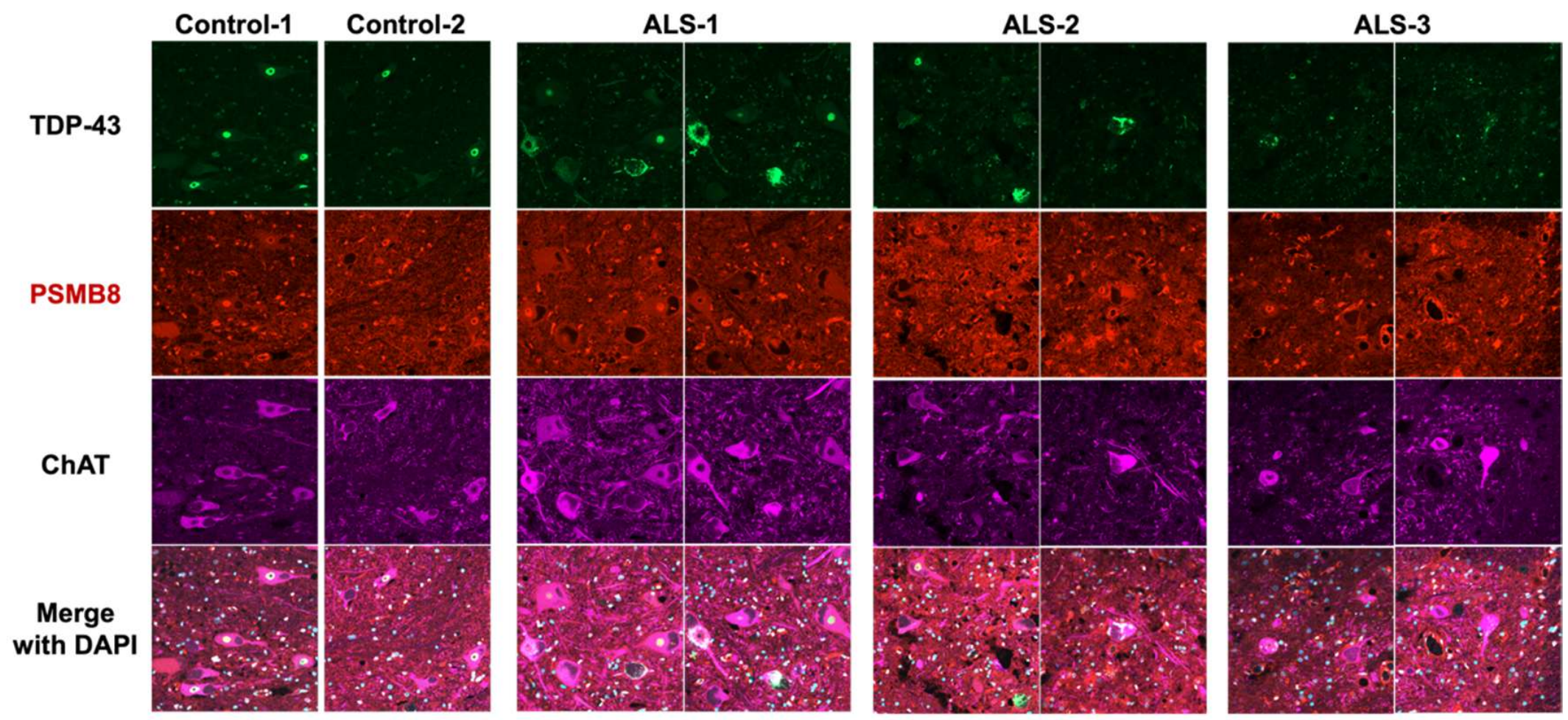

Figure for Referees only: PSMB8 staining in human spinal cords.

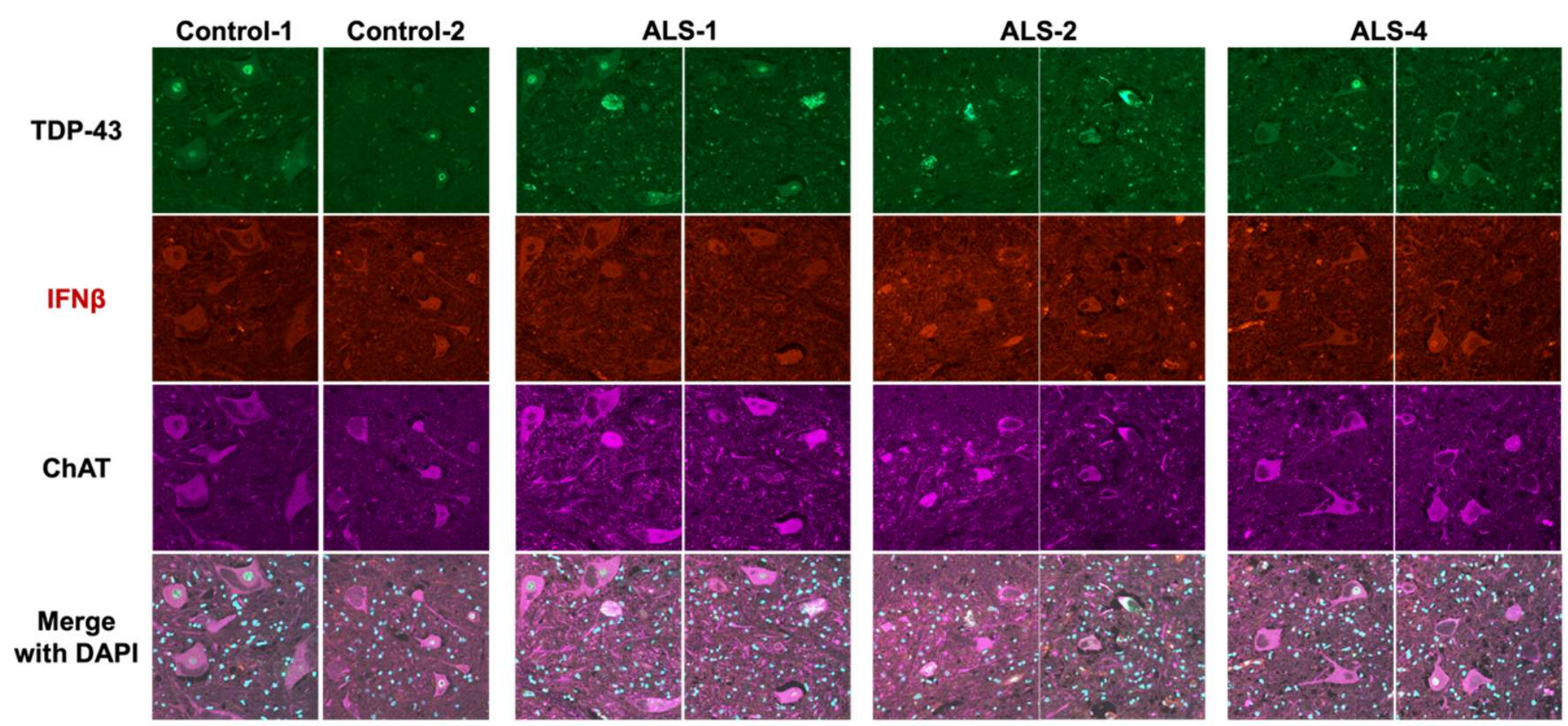

Figure for Referees only: IFNβ staining in human spinal cords.

Based on your suggestion, we have added higher magnification images showing both TDP-43 pathology-negative and positive neurons within the same low-magnification field in Fig. S10, to allow direct comparison of IFNAR1 immunofluorescence intensity between these neurons.

Q5. Please discuss potential mechanisms by which IFNβ receptor expression becomes reduced in specific neurons during disease progression. Is this due to cell-intrinsic dysfunction (e.g., transcriptional failure, feedback inhibition) or external factors (e.g., loss of paracrine signaling, microenvironmental changes)?

Response:

Based on your comment, we reviewed the literature and found a report demonstrating that viruses downregulate IFNAR1 via ER stress as a viral survival strategy (Liu et al., Cell Host & Microbe,

2009). We then demonstrated that ER stress downregulates IFNAR1 in NSC-34 cells and human iPSC-derived motor neurons (Fig. 5G). Based on these findings, we hypothesize that the downregulation of IFNAR1 in ALS motor neurons is due to cell-intrinsic dysfunction associated with ER stress.
We have incorporated this discussion into the revised manuscript (Discussion section, lines 347–352).

Q6. Figure 6 shows aging-induced immunoproteasome activation in aged mice. Did you observe corresponding induction of TBK-1 or IFNβ during this aging process? If so, what is the relationship between aging-related and disease-related immunoproteasome activation?

Response:

Unexpectedly, we did not observe a corresponding induction of TBK1 or IFNβ during this aging process. Additionally, heterozygous deletion of *Tbk1* had little effect on immunoproteasome expression levels in the lumbar spinal cord or cerebral cortex of 12-month-old mice (please see the "figure for referees only" below). Based on these results, we conclude that TBK1 does not play a significant role in immunoproteasome induction during normal aging, but is specifically involved in disease-related immunoproteasome activation. Accordingly, we have decided to remove the original Figure 6 from the revised manuscript. We appreciate the referee for the thoughtful comment.

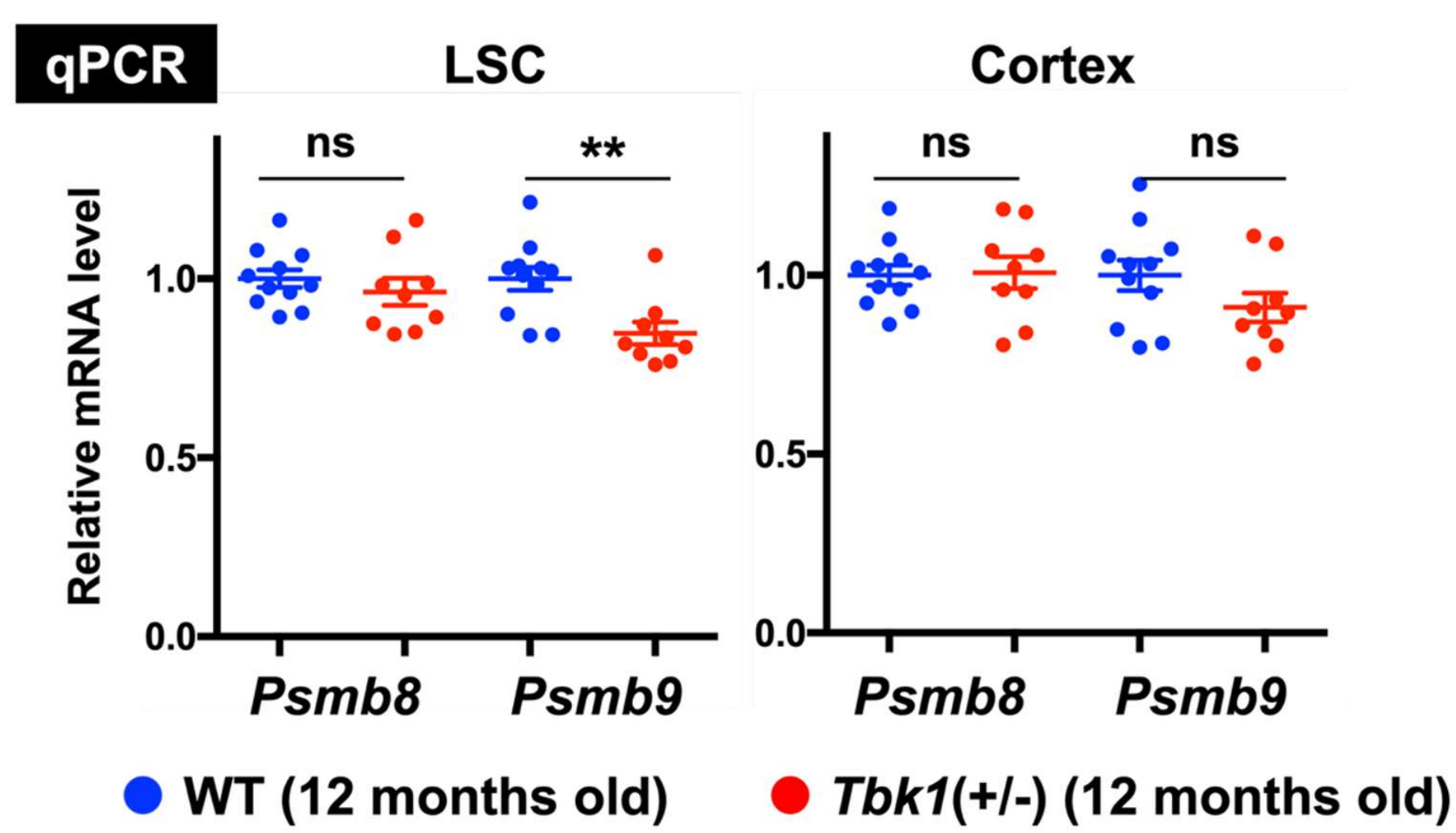

Figure for Referees only:
Heterozygous deletion of *Tbk1* had little effect on immunoproteasome expression in the lumbar spinal cord and cerebral cortex of 12-month-old mice

Q7. The authors mention that crossing TBK-1+/− mice with SOD1 mice results in earlier disease onset compared to SOD1 mice alone, but these data are either not provided or are unclear in the manuscript. Please present Kaplan-Meier survival curves for both disease onset and survival endpoints to support this claim.

Response:

As the referee pointed out, we did not provide onset or survival data in the original manuscript because we did not perform such phenotypic analyses in this study. During the revision period, we attempted to conduct this type of cohort analysis; however, the time allowed for the revision was insufficient to generate an adequate number of animals for each genotype. We would appreciate the referee's understanding of this limitation. Notably, a similar cohort analysis using the same mouse genotype has already been reported in a previous study (Brenner et al., *JEM*, 2019). Please see the "figure for referees only" below.

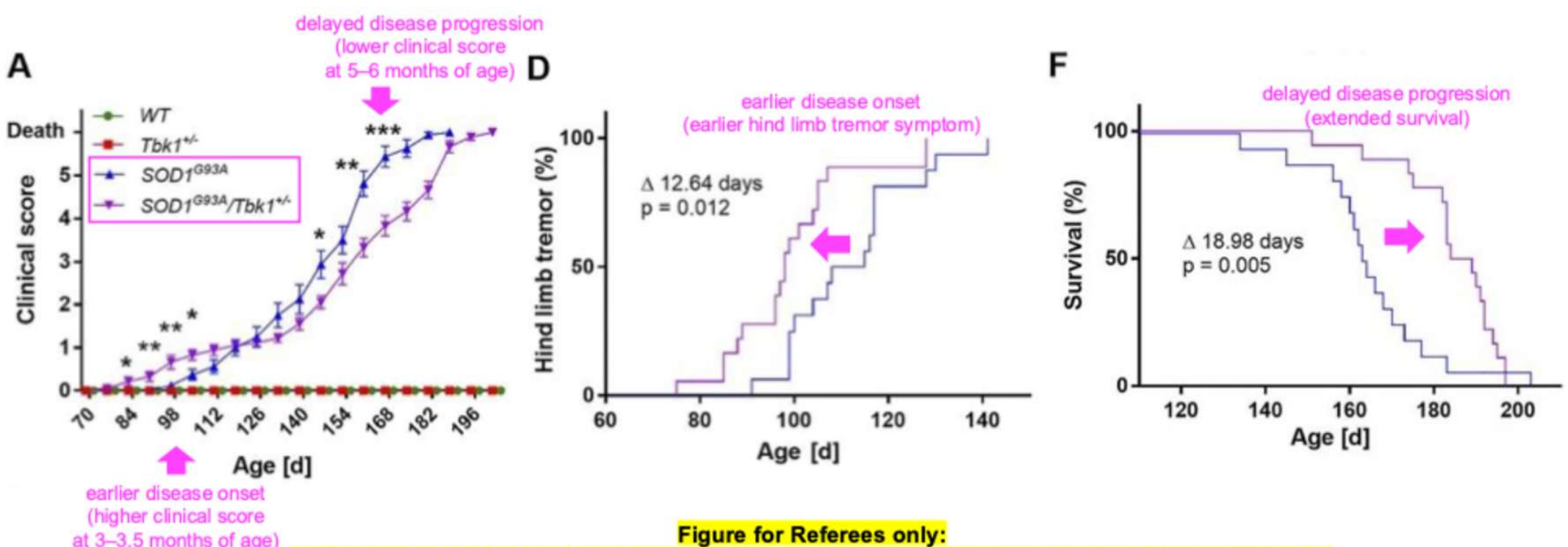

Figure for Referees only:
Heterozygous deletion of Tbk1 accelerates disease onset but extends survival in *SOD1*^{G93A} mice,
as reported in a previous study (Brenner et al., JEM, 2019). Figures are modified from the Fig.1 of the original paper.

Q8. The proposal of a novel axis in which the immunoproteasome degrades ALS pathogenic proteins via IFNβ's paracrine effect is highly innovative and impressive. It would be valuable to discuss in detail how this paracrine immunoproteasome pathway differs from and potentially complements the conventional cell-intrinsic unfolded protein response (UPR). How do these two protein quality control systems interact or regulate each other in the context of ALS?

Response:

We thank the reviewer for this insightful comment. As noted in our response to Q1, TBK1 activation is induced under cell-intrinsic proteostatic stress. We therefore speculate that stressed cells may promote IFNβ-mediated paracrine signaling, thereby enhancing immunoproteasome activity in neighboring cells and helping to alleviate proteostatic burden "at the tissue level". In this sense, this pathway may represent a tissue microenvironment-level extension of proteostasis mechanisms, distinct from canonical cell-intrinsic UPR pathways such as translational attenuation and ERAD. However, as direct evidence for this model remains limited, we have described the potential contribution of this pathway to proteostatic regulation in motor neurons in the Discussion section, while taking care not to overstate this concept . We have incorporated this discussion into the revised manuscript (Discussion section, lines 303-306).

Minor Points

1. Why was TDP-43-3xFLAG used instead of TDP-43-1xFLAG? Does the single FLAG tag affect aggregation propensity or degradation kinetics differently? Please provide justification for this choice.

Response:

We have consistently used the 3xFLAG tag in our previous studies (Watanabe et al, JBC, 2013; Oiwa et al., Sci Adv, 2023), as it provides higher sensitivity than a single FLAG tag. However, as the referee noted, the 3xFLAG tag carries a relatively high negative charge and is comparatively large, which could potentially affect aggregation propensity or degradation kinetics. To address this concern, we have confirmed that both the aggregation-prone nature of monomeric TDP-43 and its IFNβ-dependent degradation were also observed with the HA tag, a representative small tag (please see the "figure for referees only" below).

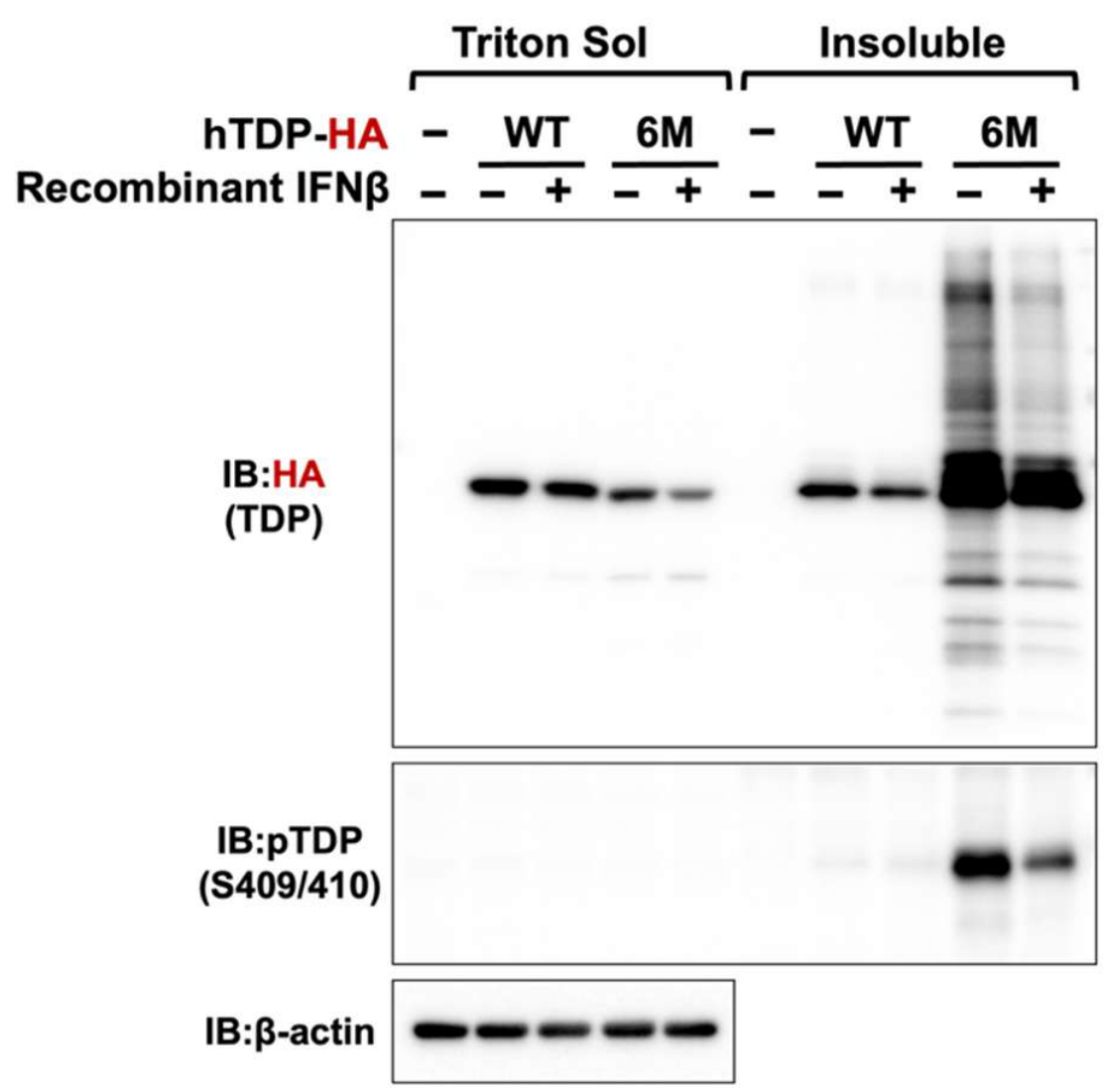

Figure for Referees only: IFNβ also reduces HA-tagged hTDP-43.

N2a cells transiently transfected with HA-tagged hTDP-43 (WT or 6M) were treated with IFN-β, fractionated into 1% Triton X-100–soluble and –insoluble fractions, and analyzed by immunoblotting.

Overall Assessment

This study presents conceptually novel and mechanistically interesting findings that advance our understanding of protein quality control in ALS. The identification of the TBK-1/IFNβ/immunoproteasome axis represents an important contribution to the field. However, the concerns raised above, particularly regarding the reliance on monomeric TDP-43, the limited

validation in motor neuron-relevant models, and the incomplete characterization of the pathway in patient tissues, need to be adequately addressed before the manuscript can be accepted for publication.

Response:

We sincerely thank the referee for this positive assessment. We believe that the revised manuscript, which now includes human iPS-derived motor neuron data, has been substantially improved through the thoughtful and constructive comments raised in this review. We acknowledge that important issues remain, particularly regarding characterization of this pathway in patient tissues, and we plan to address these in future studies.

Referee #2:

The present study by Sakai from the laboratory of Koji Yamanaka studies how TANK-binding kinase 1 (TBK1) is involved in the degradation of pathological protein forms, with dominant loss-of-function mutations in TBK1 being linked to ALS/FTD.

The study builds on two previous studies from the same laboratory:

1) Watanabe et al., (PNAS, 2023, PMID: 37967220) that showed (but not as the principle result) a reduction of activated (phospohorylated) TBK1 in brain and spinal cord tissues of sporadic ALS patients, suggesting that reduced TBK1 activity could also contribute more generally to ALS.

2) Oiwa et al., (Sci Adv, 2023, PMID: 37540751) that showed a reduced dimerization of TDP43, the main ALS pathological hallmark, in brain and spinal cord tissues of sporadic ALS patients. They further show, in vitro (Neuro2a/iPSc-MN), that an engineered TDP43 protein that lacks dimerization domains (termed TDP43-6M), acts as a monomer and leads to aggregates and nuclear exclusion, suggesting that TDP43 monomerization is part of the ALS-TDP43 pathology. They also found a specific anti-TDP43 Ab that seems to recognise, in postmortem spinal cord tissues, the (pathological) cytoplasmic monomer form and less the (normal) nuclear form.

Based on these earlier results, the present study asks how TBK1 could be implicated in the degradation of pathological monomeric TDP43. The authors use an in vitro approach to overexpress their TDP43-6M in Neuro2a cells, and test what co-overexpression of TBK1 does. In the view that they use Western blot analysis, this is an acceptable in vitro approach (iPSc-MN would be not feasable for this). Both TBK1 and TDP43-6M are tagged (Myc and FLAG) for easy recognision - although not optimal, acceptable.

The authors find then a clear reduction of TDP43-6M by TBK1 overexpression (FIG.1). But very surprisingly, this seems not to be mediated by the classic autophagy-lysosomal pathway, as Bafilomycin A1 did NOT affect the TBK1-mediated reduction of TDP43-6M (FIG.1). This is a central finding of the study and from there the authors search for an alternative pathway - basically a new way how TBK1 could be implicated in degradation, especially of pathological proteins. They focus on the known immune role of TBK1 in the induction of Type I interferons (IFNb), and find convincing evidence that TBK1-induced IFNb acts paracrine on other neuronal cells to induce immunoproteasome activity, which then leads to the degradation of TDP43-6M - thus, the pathway does not go through the (neuronal) TBK1-autophagy, but a new (neuronal) TBK1-IFNb-Immunoproteasome axis (FIGs.2-3/5).

Interestingly, the authors further show that in human ALS postmortem spinal cord tissue there is a reduction of the IFNAR1 on motor neurons compared to control, and even more so in motor neurons

with TDP43 pathology, thus suggesting a partial impairment of this proposed IFNb-IFNAR1 axis (FIG.4). In the spinal cord of mutant SOD1 ALS mice they further show that immunoproteasome specific subcomponents get induced with disease, while canonical proteasome subunits get reduced during disease, suggesting a compensatory response to degrade mutant proteins by this immunoproteasome axis (FIG.7). Finally, in a double-transgenic mutant SOD1 ALS mice with systemic Tbk1 reduction (heterozygous deletion), they show that this induction of immunoproteasome specific subcomponents is reduced, while they measured an increase in ubiquinated proteins, further suggesting that a TBK1-IFNb-IFNAR1-Immunoproteasome axis is implicated in protein degradation (FIG.7).

As such, this is a highly interesting study, both on a fundamental level for TBK1 functions, and with respect to pathological protein accumulations in ALS. While the study uses principally in vitro neuronal cell line systems, the effort was done to go to human postmortem tissues as well as ALS mice. To link the immune cell function of TBK1 (activation of IFNb) to an alternative protein degradation function of TBK1, not in autophagy as classically known, but instead potentially through the immunoproteasome, is very interesting, and opens up new pathways how we might be able to target degradation of pathological proteins in ALS.

However, there are some issues that need to be clarified, and expanded upon, before a possible publication can be considered.

Response:

We appreciate your positive evaluation of our initial manuscript and many helpful suggestions to strengthen our study. In this revision, we have revised the figures and text based on your feedback, as listed below:

Major Issues

- 1 → Fig. S5 (new data)
- 2 → Fig. S8A (moved from the previous Fig. S6), Fig. S8B (new data), Fig. S9 (new data)
- 3 → Fig. 6A (new data)
- 4 → withdrawal of the original Fig. 6
- 5 → Fig. 7C–F (new data)
- 6 → description only

Minor Issues

- Fig. 1H, 1J, 2A, 2G, 3I, 3J, 4A, 4D, 4E, 4I, 4J, 6A, 6B, 6L (revised data presentation)

・Fig. 3A (correction of typo)

MAJOR ISSUES:

• FIGURES 1:

1) FIG.1C: as this is a central finding (Baf A1 does NOT block the TBK1-driven TDP43-6M reduction), it would be great to have some sort of a "positive" control for Baf A1 - to show that indeed autophagy is blocked. Also, maybe this is obvious to autophagy-experts, but why does Baf A1 not block induction of p62/LCIII by TBK1 overexpression ? Likewise, in the view of their results in Fig.5A (block of IFNb induced TDP43-6M reduction by proteasome inhibition), it would be of course very convincing to do here a proteasome inhibition, side-by-side with the autophagy inhibition.

Response:

Regarding the first point (positive control for Baf A1), we would like to draw attention to the LC3-II bands of the 1st (Mock-DMSO) and the 3rd (Mock-Baf A1) lanes in Fig. 2C (formerly Fig. 1C): Baf A1 visibly increased LC3-II levels, which is a well-established indicator of autophagosome accumulation, reflecting the blockade of autophagy-lysosomal degradation. We have described this in the revised manuscript (**lines 113–114**).

Regarding the second point (why Baf A1 does not block the induction of p62/LC3-II by TBK1 overexpression), Baf A1 is an inhibitor of V-ATPase, which is required for lysosomal acidification, and therefore blocks lysosomal degradation but does not inhibit autophagosome maturation or substrate incorporation into autophagosomes. Thus, we believe it is reasonable that TBK1 induces p62 phosphorylation (note that p62 is a direct substrate of TBK1) and LC3 lipidation (autophagosome maturation) even in the presence of Baf A1.

Regarding the third point (performing proteasome inhibition alongside autophagy inhibition), we have addressed this point and added the data in Fig. S5. In this experiment, we used a stable cell line expressing TDP^{6M} in a doxycycline-dependent manner, rather than a transient expression approach, to minimize transfection-associated toxicity and ensure consistent results.

• FIGURE 4:

2) FIG.4A/B: while the IFNAR1-immunos in the human tissues (Fig.4C/D) are quite convincing, the stains in the mouse spinal cord and especially in cerbral cortex are less so. As it is shown, IFNAR1 is mainly expressed on motor neurons. Should it not also be expressd on microglia ? What does published RNAseq data from spinal cord microglia indicate ? What does the Allen Brain Atlas show ? Or could you do a confirmatory RNAscope

for IFNAR1 - just to be sure that this antibody is really specific - as this is a major point for this study. The same is also an issue for the human tissue, although I am aware that this is much more difficult due to limited material (but maybe check existing snRNAseq data for expression in MN vs microglia).

Response:

We appreciate this thoughtful comment. We agree that IFNAR1 should also be expressed in microglia. Based on this comment, we performed co-immunostaining of IFNAR1 with ChAT and a microglial marker IBA1 in mouse spinal cord tissue and confirmed IFNAR1 signals in IBA1-positive cells. These results have been incorporated into the revised manuscript as Fig. S9 and we have also mentioned microglial IFNAR1 in the text (line 183). Note that IFNAR1 staining in Fig. 5A was performed on slide-mounted sections, whereas immunostaining in Fig. S9 was performed using a free-floating method to enhance sensitivity; this accounts for the slight differences in the staining patterns of IFNAR1 between the two figures.

We also examined published data to investigate IFNAR1 expression across CNS cell types. As shown in the previous Fig. S6 (currently renumbered as Fig. S8A), the integrated single-cell/nucleus RNA-sequencing data from various tissues in the Human Protein Atlas (http://www.proteinatlas.org/celltype) (Karlsson et al, 2021) indicates that IFNAR1 mRNA is broadly expressed across various CNS cell types, including neurons and glial cells. Additionally, we examined human spinal cord snRNA-seq data (Yadav et al., Neuron, 2023). As shown in Fig. S8B, this dataset shows that spinal motor neurons express *Ifnar1* at a similar level to other cell types, including microglia. Collectively, these published data indicate that motor neurons express *Ifnar1,* at least at the mRNA level. However, we acknowledge that this evidence does not conclusively demonstrate that the IFNAR1 antibody signal in spinal motor neurons is specific. Further validation will be required in the future. To avoid overstatement in this revision, we have revised the description from "primarily expressed in neurons" to "expressed in neurons" (Figure Legend; line 970).

Notably, regardless of whether the protein expression level of IFNAR1 in motor neurons is higher than that in other cell types, we are confident that motor neurons do express IFNAR1, as demonstrated by the responsiveness of human iPSC-motor neurons to recombinant IFNβ in Fig. 4J, 6G, 6H, and 6K.

• FIGURE 5:

3) FIG.5A: The authors show very convincingly that proteasome inhibition fully blocks the IFNb induced TDP43-6M reduction (stable/inducible NSC34 lines). What would have been even more convincing, would be to show in parallel that autophagy inhibition should NOT affect this. I am aware that this has been somehow shown using transient TDP43-6M+TBK1 in Neuro2a cells in Fig.1C, but not side-by-side.

Response:

As in Fig. S5, we examined the effects of autophagy and proteasome inhibition side by side and demonstrated that inhibition of the proteasome, but not autophagy, fully blocks the IFNβ-induced reduction of TDP-43^{6M} (Fig. 6A). We believe that this result further strengthens our conclusion. We thank the referee for this valuable suggestion.

• FIGURE 6:

4) Overall, this is a bit difficult to understand. The Idea is to show that during aging in healthy mice, the IFNb downstream genes and immunoproteasome specific subunits get upregulated, in spinal cord and cerebral cortex. But how is TBK1 regulated during aging ? And the more classic autophagy genes ? Also, while this is interesting (concerning the presented data), it would only be really powerful when we see these regulations on a cellular level. While there was an effort for PSMB8 (immunoproteasome), the immunos are not really convincing. RNAscope might be an alternative. Also, there are published RNAseq studies of (healthy/normal) aging of both motor neurons and spinal cord microglia in mice (Castro, 2023, PMID: 37154159; Castro, 2024, PMID: 37737058) - the data could be verified in there. With respect to compare aging with ALS, it would be interesting to check if the IFNAR1 is downregulated on motor neurons during normal aging in mice (using immunos, as in Fig.4A), comparable to what the authors showed for ALS MN in human postmortem tissues. Again, the aging link is interesting but stands a bit "alone" right now. Also as a comment, 12 months in mice is rather middle-aged than really "aged/old" as B6 mice live up to 30 months.

Response:

We thank you for these constructive comments. Several points raised here overlap with those raised by Referee #1 (Q6), and we refer the referee to our response to that comment for additional details.

As noted in our response to Referee #1, we were unable to obtain convincing evidence that TBK1 plays a significant role in immunoproteasome induction in this middle-aging model. We also note, as the referee correctly points out, that 12-month-old C57BL/6 mice are more accurately described as middle-aged rather than aged. Based on these considerations, we conclude that TBK1 plays a selective role in disease-related, rather than aging-related immunoproteasome activation, and have accordingly removed the original Fig. 6 from the revised manuscript.

We fully agree that the remaining questions raised by the referee are important and represent valuable directions for future investigation.

• FIGURE 7:

5) FIG.7A-E: What is very impressive here is this finding that the immunoproteasome subunits get induced with ALS disease in mutant SOD1 ALS mouse spinal cord, while the canonical proteasome subunits rather get reduced, and TBK1 reduction reduces the immunoproteasome induction. Again,

also I understand the difficulty, but cell specifity would be interesting for PSMB8/9 - maybe RNAscope is possible (if fixed, floating sections are still available, this could be doable) - if not in the full Tbk1/G93A mice, then at least for the base G93A ? The paper clearly claims that this happens in MN, and the immunos in Fig.6E are not so convincing.

FIG.7F: Here the authors assess hSOD1, p62 and ubiquinated proteins in the Tbk1-het/G93A mouse spinal cord by Western, and find no effect on hSOD1 and p62, while they find increased ubiquinated proteins in the insoluble fraction - thus, they claim that autophagy is not affected but proteasome function reduced by Tbk1 reduction (supporting their idea that the TBK1-IFNb-IFNAR1-immunoproteasome axis is active in baseline ALS mice). However, we know from the Brenner 2019 study (PMID: 30635357) that early in the disease p62 is clearly increased in the same Tbk1-het/G93A mouse, while at later stages this seems to be inversed, comparable to the present study (although Brenner used immunos, while the present study used Western). Also in human TBK1-ALS-iPSc-MN, there is increased p62 detectable (PMID: 30939964). More experiments have to be done here, or the data be put more carefully into correlation with existing data. The claim that only the immunoproteasome axis is affected by Tbk1-reduction in ALS mice, seems too strong, likely both (TBK1/autophagy & TBK1/immunoproteasome) are affected, and contribute to the disease. But again, the authors finding that the TBK1/immunoproteasome axis is affected/implicated is important and novel.

Also - as mutant hSOD1 is itself not affected by Tbk1-reduction (soluble/insoluble fractions are not changed), what are the proteins that are ubiquinated and thus would lead to increased toxicity - or is that not what the authors somehow would claim here ?

Response:

Regarding the first point, we attempted to establish RNAscope in-house, but we were unable to optimize the protocol for detecting immunoproteasome subunits during the revision period. As an alternative approach, we undertook additional expression analysis of canonical proteasome and immunoproteasome subunits in laser microdissection (LMD)-isolated motor neurons from SOD1-G93A mice (Fig. 7C–F). Consistent with the results obtained from whole spinal cords (Fig. 7A, B), LMD-isolated motor neurons exhibited reduced expression of the canonical proteasome β-subunits Psmb6 and Psmb7, together with increased expression of the immunoproteasome β-subunit Psmb8 (Fig. 7F). These data indicate that induction of the immunoproteasome in response to proteostatic stress occurs in motor neurons. We have incorporated these new findings, together with the corresponding Results text (lines 234–239) and Fig. 7C–F, into the revised manuscript.

Regarding the second point (moved to Fig. 7J), we agree with the referee and were also surprised that p62 levels were not increased in $SOD1^{G93A}/Tbk1^{+/-}$ mice compared with $SOD1^{G93A}$.

We do not interpret this result as indicating that autophagy is unaffected by Tbk1 reduction. One

notable difference from the study by Brenner and colleagues is the age of the mice: they analyzed p62 at P50, whereas we examined p62 and ubiquitin at 3.5 months. Therefore, it is possible that the contribution of TBK1 to autophagy and proteasomal pathways depends on the disease stage—for example, autophagy may be more prominent before disease onset (e.g., P50), whereas proteasomal regulation may become more relevant around disease onset (e.g., 3.5 months). To further investigate the role of TBK1 in autophagy in this model, we attempted to reproduce the increase in p62 inclusions at P50 reported by Brenner and colleagues. However, unexpectedly, we were unable to observe an increase in the size or number of p62 inclusions in *SOD1*^{G93A}/*Tbk1*$^{+/-}$ mice (please see the "figure for referees only" below). The reason for this discrepancy is currently unclear; the limited sample size (n=3 per genotype) may have contributed. In any case, we believe that both the autophagy and immunoproteasome pathways are affected by Tbk1 deregulation and that the relative contribution of each degradation pathway may depend on the disease stage.

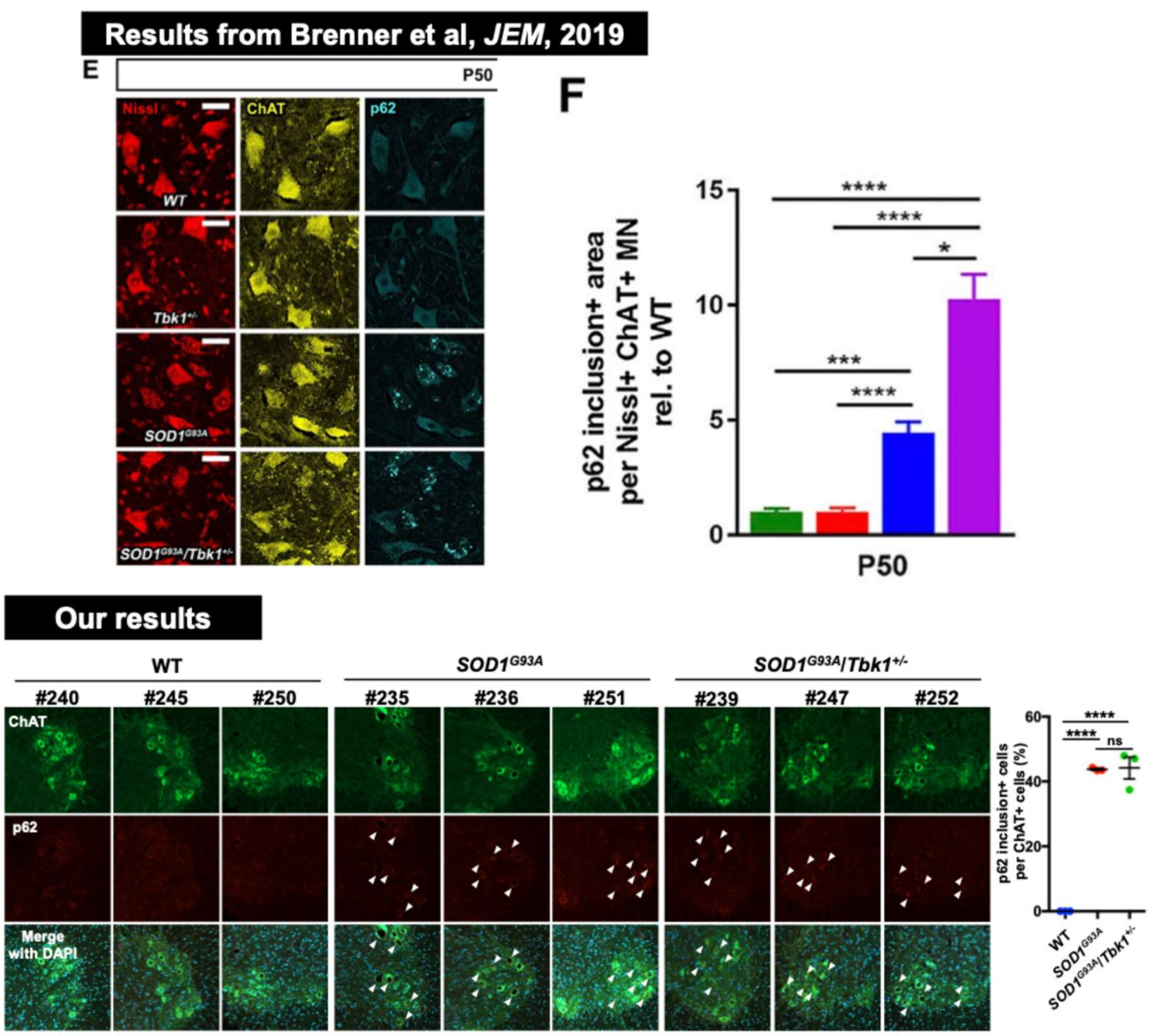

Figure for Referees only: Heterozygous knockout of Tbk1 did not increase p62 inclusion in SOD1^{G93A} at p50.

White arrowheads indicate p62 inclusion in ChAT-positive motor neurons.

Regarding the third point (the identity of the ubiquitinated proteins in the insoluble fraction when mutant hSOD1 itself is unaffected by Tbk1 reduction), we cannot definitively identify the major components of the insoluble ubiquitinated proteins. We speculate that they consist of many different protein species, and that an LC-MS/MS approach will be required to identify them in detail. We were also surprised by the finding that hSOD1 was not increased in Tbk1-het/G93A mice, although this is consistent with the previous report by Brenner and colleagues, who showed unchanged hSOD1 levels in the same Tbk1-het/G93A mice by both immunoblotting and immunohistochemistry (Brenner et al. JEM, 2019, Fig. 3I–K & Fig. S2E and F).

6) TITLE: While the title is acceptable, the addition "in ALS." seems a bit strong. The actual functional assay was only done in cell lines, and the in vivo data stems from a reduced IFNAR1 stain in ALS postmortem tissues and less immunoproteasome induction in Tbk1-het/G93A mice (plus increased amounts of insoluble ubiquinated proteins).

Response:

We thank the referee for this suggestion and appreciate the concern. Accordingly, we have revised the title to "**TBK1 eliminates aggregation-prone monomeric TDP-43 through the IFNβ-immunoproteasome pathway in ALS <u>models</u>**." We hope that this revised title adequately addresses the referee's concern.

<u>MINOR ISSUES:</u>

• Fig.1A: While the explanations in the main text are easy to follow, the reading of the actual Figures with its Western Blots is less intuitive. The color blue/red is used for different things, thus not too helpful. e.g. 1A, the "FLAGtbk1/FLAGmock ratio" is not very intuitive.

Response:

We thank the referee for the comment. As pointed out, the use of blue/red dot plots with bars to represent different types of data makes the figures less intuitive to interpret. We have therefore revised all protein fold-change data, such as the "$FLAG_{TBK1}/FLAG_{Mock}$ ratio," from "dot plots with bars" to "dot plots without bars", to more intuitively distinguish them from data representing relative values, such as "Relative FLAG" or "Relative pTDP." Please refer to Figs. 1H, 1J, 2A, and related panels. We hope that this modification will facilitate clearer interpretation of the figures by readers.

• Fig.2A: typo "konckdown"

Response:

We have corrected the typo. We thank the referee for pointing this out.

• All SFigs1-9 seem blurry ?

Response:

This issue has now been corrected in the revised manuscript. We apologize for the low resolution in the original version.

Referee #3:

The manuscript by Sakai et al., entitled "TBK1 eliminates aggregation-prone monomeric TDP-43 through the IFN-β-immunoproteasome pathway in ALS," investigates the role of TBK1 in ALS (amyotrophic lateral sclerosis) pathogenesis. Building on their previous work (Oiwa et al., Sci Adv 2023) characterizing an aggregation-prone TDP-43 mutant (TDP-43 6M), the authors show that TBK1 overexpression reduces, and TBK1 knockdown increases, insoluble TDP-43 6M. Although TBK1 has been thought to function primarily in the autophagy-lysosome pathway and ALS-FTD mutations appear to disrupt mitophagy (Harding et al., PNAS 2021), the authors uncover that a TBK1-mediated paracrine signaling mechanism is required to reduce TDP-43 6M aggregation. They further show that TBK1 acts through STING and IRF3 to induce secretion of IFN-β, which activates IFN-β receptors and upregulates immunoproteasome expression. This TBK1-IFN-β-immunoproteasome pathway appears to be specific to neuronal cell lines (SH-SY5Y, Neuro2a, and NSC-34) and not present in non-neuronal lines (HEK293 and HeLa). Intriguingly, immunoproteasome subunits exhibit an age-dependent increase in mouse spinal cord and cerebral cortex. Furthermore, IFNAR1, a subunit of the IFN-α/β receptor (IFNAR), is present in neurons within the spinal cord, and IFNAR1 expression appears reduced in motor neurons of ALS patients. Finally, the authors crossed SOD1-G93A mice with TBK1-heterozygous knockout mice and found that expression of canonical proteasome subunits decreased while immunoproteasome subunits increased in SOD1 mice. Moreover, the induction of immunoproteasome expression was blunted in SOD1-G93A/TBK1-heterozygous knockout mice, which exhibited higher ubiquitinated protein loads.

Overall, this is an interesting and well-executed study that reveals a potentially novel mechanism involving the TBK1-IFN-β-immunoproteasome axis in ALS pathogenesis. The manuscript is well written, with clear explanations and logical flow. The experiments are thoughtfully designed and appropriately controlled. I congratulate the authors on a job well done. The paper will be of broad interest to this journal's readership as well as to researchers in neurodegeneration. This referee is excited about these results and believes they will open a range of follow-up studies. Accordingly, I will limit my comments to the current data presented. Below are comments and suggestions to strengthen and improve the manuscript for the authors' and editor's consideration.

Response:

We appreciate your positive evaluation of our original manuscript and your thoughtful suggestions

regarding statistical analyses and additional experiments to strengthen our conclusions. In this revision, we have revised the figures and manuscript accordingly based on your feedback, as listed below:

Major comments/suggestions

・1 → Fig. 5E and 5F (revised data presentation), removal of the original Fig. S7

・2 → description only

・3 → Fig. 4F–J (new data), Fig. 5G (new data), Fig. 6G, H, and K (new data)

・4 → description only

Minor points

・1 → withdrawal of the original Fig. S5

・2 → description only

Major comments/suggestions:

1. Statistical analyses for human motor neurons (Figure 4E-F, Figure S7)

I appreciate that the authors provided the original data in Figure S7. If I understand correctly, the data shown in Figures 4E and 4F were pooled across subjects/patients, which inflates statistical power because motor neurons from the same subject/patient are not independent observations. The authors should consider a linear mixed-effects model or other mixed-effects approaches, or compute subject-level means and treat these as independent units (simpler but with lower power). The SuperPlots guidelines (Lord et al., J Cell Biol, 2020; doi: 10.1083/jcb.202001064) provide useful recommendations and presentation for representing subject-level data.

Response:

The referee is correct, and we apologize for the inadequate statistical analysis in the original submission. Following the referee's suggestion, we performed a linear mixed-effects model-based analysis using R and presented the data in a SuperPlots-style format (Fig. 5E and F). Regarding Fig. 5E, a linear mixed-effects model with patient as a random effect revealed that IFNAR1 intensity was significantly lower in the ALS group than in the control group ($p = 0.0165$). Regarding Fig. 5F, the model also revealed a significant effect of group ($p = 9.27 \times 10^{-12}$). Post hoc comparisons with Tukey's adjustment showed that intensity was significantly lower in both ALS Patho(-) and ALS Patho(+) groups than in the control group ($p < 0.0001$), and was also significantly lower in ALS Patho(+) than in ALS Patho(-) ($p = 0.0068$). We appreciate your constructive comment and have also revised the Method and Figure legend section accordingly **(lines 614–617, lines 984–989)**.

2. Strengthen the age-dependent increase of immunoproteasome expression (Figure 6)

The attempt to validate increased expression of immunoproteasome subunits in neurons by immunofluorescence (Figure 6E and 6F) is appreciated; however, immunofluorescence is intrinsically limited for precise quantification. Would the authors consider single-molecule RNA FISH (smFISH) combined with neuronal markers (ChAT and CTIP2)? This would corroborate the RT-qPCR data (Figure 6C-D) and further strengthen the claim.

Response:

We appreciate the referee's constructive suggestion. However, we decided to remove the original Figure 6 from the revised manuscript because we were unable to obtain convincing evidence that the age-dependent increase in immunoproteasome expression is TBK1-dependent (please refer to Q6 from Referee #1 and our response). We regret that we were unable to incorporate this suggestion into the revised version, and we recognize it as an important direction for future investigation.

3. Strengthen the neuronal specificity of IFN-β-mediated immunoproteasome induction (Figure 5)

The finding that IFN-β induces PSMB8 and PSMB9 in neuronal cell lines (SH-SY5Y, Neuro2a, NSC-34) but not in HEK293 or HeLa cells is compelling. To reinforce the claim in bona fide neurons, please consider testing iPSC-derived neurons (as in Oiwa et al., Sci Adv 2023) and/or primary neurons.

Response:

We thank the referee for this suggestion. We have confirmed IFNβ-dependent immunoproteasome induction in primary spinal neurons (Fig. S11). Moreover, we have established a human iPSC-derived motor neuron model (Fig. 4F and G) and demonstrated that IFNβ also induces the immunoproteasome in these motor neurons, whereas the effect was negligible in undifferentiated iPSCs (Fig. 6G, H, and K). We have also added additional data from iPSC-derived motor neurons to Fig. 4H–J and Fig. 5G to further strengthen our central findings.

4. Clarification of TBK1 effects on wild-type TDP-43 (Figure 1A and 1C)

The data provided in this manuscript convincingly suggest that TDP-43 6M (aggregation prone) is degraded via the TBK1-IFN-β-immunoproteasome pathway. However, insoluble wild-type TDP-43 also appears reduced upon TBK1 overexpression (Figure 1A). Please clarify whether wild-type TDP-43 is cleared via autophagy-dependent or autophagy-independent mechanisms by performing experiments analogous to Figure 1C.

Response:

We thank the referee for the comment. We attempted to clarify whether wild-type TDP-43 is degraded via autophagy or proteasome pathway in the context of TBK1 overexpression. For this experiment, we used a stable cell line, as described for the 6M mutant in Fig. S5, to minimize transfection-related toxicity and to ensure consistent results. However, TBK1 overexpression only minimally reduced wild-type TDP-43 levels in the stable cell line (please see the "figure for referees only" below), and

therefore we were unable to reach a definitive conclusion.

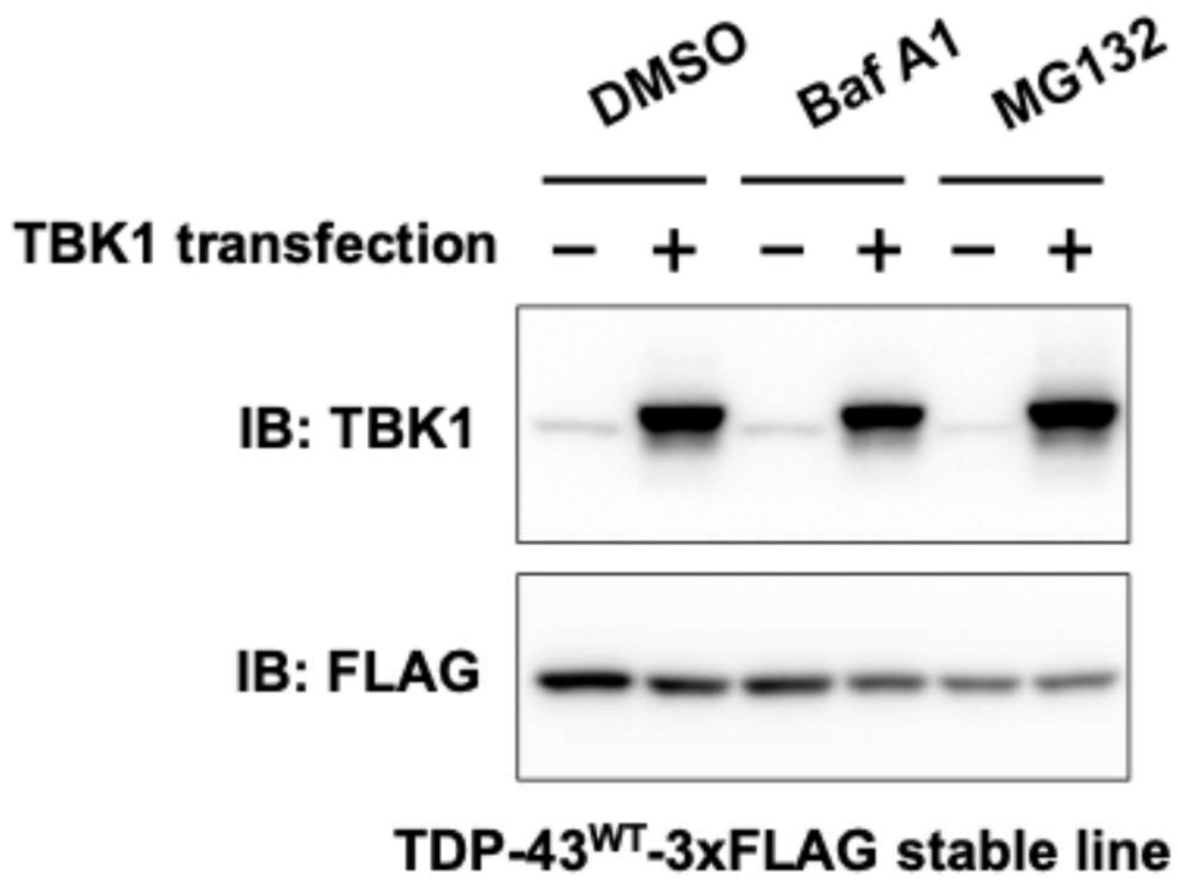

Figure for Referees only: TBK1 overexpression had little effect on wild-type hTDP-43 levels in a stable cell line.

Because the effect of TBK1 on wild-type TDP-43 levels was modest even under conditions of transient overexpression, compared with that on the 6M mutant (Fig. 2A), we speculate that the lower expression level in the stable cell line further attenuated the effect. Notably, a similar trend was observed in the IFNβ treatment experiments, in which IFNβ reduced transiently overexpressed wild-type TDP-43 (Fig. 4A) but not stably expressed wild-type TDP-43 (Fig. 4C). Although we also attempted to address this question using a transient overexpression model, the combination of transfection-related toxicity and inhibitor toxicity resulted in inconsistent outcomes. Given these technical limitations, a definitive mechanistic conclusion regarding wild-type TDP-43 degradation remains elusive, and we plan to address this in future studies. We would appreciate the referee's understanding of this limitation.

Minor points:

1. The effect of recombinant IFN-β on mutant SOD1 in Neuro2a cells (Figure S5) is less uniform than on TDP-43 (Figure 3): soluble SOD1-G93A and insoluble SOD1-G85R are reduced, whereas the other fractions are less affected. Please clarify this in the text (Lines 142-143).

Response:

We appreciate this comment. Frankly, we were also uncertain about how best to describe the observed non-uniform effect on SOD1. Given that $hSOD1^{G93A}$ protein levels were not altered by heterozygous deletion of Tbk1 in mice, and to avoid potential confusion for readers, we have decided to remove these data from the revised manuscript. Instead, following Referee #1's comment (Q2), we analyzed the effect of IFNβ on various TDP-43 mutants and included the results in the revised manuscript (Fig. S7, lines 155–157). We hope that this decision is acceptable.

2. It would be helpful to show TDP-43 levels in total lysate (before any spin) for selected experiments- especially those showing reductions in both soluble and insoluble fractions (e.g., Figures 1A, 1G, 3A)- to exclude the possibility that total TDP-43 levels are altered by TBK1 expression. This is likely not the case, but presenting total lysate controls would confirm it.

Response:

We appreciate this suggestion. We observed and stated that TDP-43 was reduced in both the soluble and insoluble fractions by TBK1 overexpression and IFNβ treatment. Therefore, we believe that total TDP-43 levels were also reduced by TBK1 overexpression and IFNβ treatment. Indeed, the new Fig. S7 shows results from total lysates, demonstrating that IFNβ reduces total TDP-43 levels, particularly for the monomeric mutant (6M). We therefore do not consider the reduction of total TDP-43 to be a concern that affects our conclusions. We apologize if our response has not fully addressed the referee's concern, and we welcome any further clarification.

Dear Prof. Yamanaka.

Thank you for the submission of your revised manuscript. We have now received the enclosed reports from 2 referees and I am happy to say that both support its publication now. Referee 1 only has some more minor suggestions that I would like you to incorporate before we can proceed with the official acceptance of your manuscript. Please submit a point-by-point response to all final comments with your final ms.

A few editorial requests will also need to be addressed:

- Please upload Figures 1 - 8 as separate, high resolution figure files.

- The email address for Kotaro Oiwa (oiwa.kotaro.e1@f.mail.nagoya-u.ac.jp) is not valid. Please add a valid email address.

- If the Japan Society for the Promotion of Science (JSPS) is a funder, please do entered it as a funder in our online submission system.

- Please remove the AUTHOR CONTRIBUTIONS from the manuscript file. All contributions should be entered during online ms submission.

- Please upload the source data checklist with your source data.

- Please remove BIORENDER from the Acknowledgments and move it to a dedicated section in the Methods using this format:
Graphics:
(some of the... OR Figure #... OR synopsis) Graphics were created with BioRender.com.

- Please note that the exact p values are not provided in the legends of 1E,F,G,H,I,J,K; 2A,B,C,E,G; 3B,C,D,E,F,G,H,I,J; 4A,B,C,D,I; 5E,F,G; 6A,B,D,E,J,K,L,M; 7A,B,E,F,G,H,I,J. Please provide exact values as reasonable.

I would like to suggest some minor changes to the title and abstract that needs to be written in present tense. Do you agree with this:

TBK1 eliminates aggregation-prone monomeric TDP-43 through an IFNβ-immunoproteasome pathway in ALS models

Loss-of-function mutations in TANK-binding kinase 1 (TBK1) cause amyotrophic lateral sclerosis (ALS) and frontotemporal dementia (FTD), characterized by cytoplasmic aggregation of TAR DNA-binding protein 43 (TDP-43), known as TDP-43 pathology, but the mechanisms beyond impaired autophagy remain undefined. Here, we identify a TBK1-IFNβ-immunoproteasome axis as a novel autophagy-independent proteostatic pathway regulating TDP-43 clearance. TBK1 is activated by aggregation-prone monomeric TDP-43 via physical interaction, and this activation alleviates TDP-43 pathology in neuronal cells. TBK1 subsequently induces IFNβ expression [OK? Or secretion?], which upregulates the immunoproteasome to promote degradation of monomeric TDP-43 in neurons, including human iPSC-derived lower motor neurons. Importantly, IFNβ receptor expression is downregulated in spinal motor neurons from ALS patients with TDP-43 pathology. Furthermore, heterozygous Tbk1 deletion in SOD1G93A mice impairs immunoproteasome induction and increases polyubiquitinated protein accumulation in spinal cords, supporting the in vivo relevance of this pathway. Collectively, our findings reveal that impairment of the TBK1-IFNβ-immunoproteasome axis represents an autophagy-independent mechanism contributing to the development of TDP-43 pathology in ALS and FTD.

EMBO press papers are accompanied online by A) a short (1-2 sentences) summary of the findings and their significance, B) 2-3 bullet points highlighting key results and C) a synopsis image that is exactly 550 pixels wide and 300 pixels high. The synopsis image should provide a sketch of the major findings, like a graphical abstract. Please note that text needs to be readable at the final size. Please send us this information along with the final manuscript.

I look forward to seeing a final version of your manuscript as soon as possible.

Best regards,
Esther

Esther Schnapp, PhD
Senior Editor
EMBO reports

Referee #1:

The authors have responded to my comments in an exemplary manner. The new Figure 1, which establishes that cytoplasmic monomeric TDP-43 associates with TBK1 and promotes its phosphorylation, supplies the mechanistic starting point that the original manuscript lacked and completes the logic of the proposed axis. The newly established human iPSC-derived lower motor neuron model, the laser microdissection analysis of motor neurons from SOD1-G93A mice, the side-by-side comparison of autophagy and proteasome inhibition, and the demonstration that ER stress reduces IFNAR1 all strengthen the study considerably. I also wish to commend the authors for withdrawing the original Figure 6 rather than retaining an underdetermined claim, and for presenting their negative and inconclusive results transparently in the response. Notwithstanding these substantial improvements, several points remain, as described below.

Major points

1. Quantification of the new Figure 1
The proposition that monomeric TDP-43 activates TBK1 is now one of the central claims of the paper, yet the evidence for their association in Figure 1 is largely qualitative, resting on representative confocal images. The conclusion would be more persuasive if supported by a more quantitative presentation of the existing data. For Figure 1C, reporting the number of patients and control subjects examined, the number of motor neurons assessed, and the proportion showing colocalization would similarly strengthen the panel. Since the images have already been acquired, I anticipate that this can be addressed without additional experiments

2. Calibration of the claim of a physical interaction
At lines 91-92 the authors state that colocalization in patient spinal motor neurons provides the first in vivo evidence of a TDP-43-TBK1 interaction. However, colocalization at the resolution of confocal microscopy does not demonstrate an interaction, and the same reservation applies to the corresponding statement in the Abstract. Furthermore, DSP is a cell-permeable crosslinker that stabilises indirect as well as direct complexes, so the co-immunoprecipitation data in Figure 1E-G establish association within a complex rather than direct binding. The possibility that p-TBK1 is passively recruited into, or sequestered by, cytoplasmic TDP-43 assemblies is not formally excluded by the present data.
I do not regard this as requiring additional experiments, but the wording should be adjusted. I would suggest describing the human data as colocalization consistent with an association in vivo, and replacing "via a physical interaction" with "in association with" or "via a physical association" in the Abstract, Results, and Figure 8 legend. This would leave the authors' conclusion intact while placing it on secure ground.

Minor points

(i) In Figure 7J, the one-way ANOVA includes groups with n = 2 (WT and Tbk1+/−). The principal comparison between SOD1-G93A and SOD1-G93A/Tbk1+/− (n = 6 each) is unaffected, but the two-animal groups should be presented descriptively rather than entered into the statistical model, or the limitation should be stated in the legend.
(ii) The Methods state that outliers were identified by Grubbs' test and excluded from all analyses. With group sizes of three to five, this procedure can appreciably influence the outcome. Please state how many data points were excluded and from which panels.
(iii) The linear mixed-effects analysis in Figure 5F is based on two control and three ALS donors. Estimation of a random effect from so few levels is unstable, and a brief acknowledgement of this in the legend or Methods would be appropriate. The analysis in Figure 5E, with four and six donors, is more secure.
(iv) The demonstration that HA-tagged TDP-43 behaves comparably to the 3xFLAG construct (response to my minor point 1) is a useful control and would be better placed in the Appendix than restricted to the referees, since other readers will raise the same question.
(v) I accept the authors' account of their unsuccessful attempts to characterise PSMB8 and IFNβ in postmortem tissue, and I do not ask for further work on this point. However, because the antibody screening is documented only in the response to referees, the Discussion should state explicitly that expression of the immunoproteasome and IFNβ in patient tissue remains uncharacterised and constitutes a limitation of the study.
(vi) Line 533: "Eequal amounts".

Referee #2:

The authors have made a huge effort to respond to all the comments of the 3 reviewers.

Regarding my own comments (Referee#2), all my concerns have been adequately resolved by the authors and I thank them for the effort and additional experiments. I congratulate them for this excellent study that brings important new findings to the field.

Details about their responses - all accepted:
1) My Comment Figures 1/5:
They explained well were to find the asked for controls in the WBs and added a new SuppFig5 with side-by-side WBs showing the effects of blocking autophagy or proteasome in their system.
2) My Comment Figure 4:
They added a new IF (SuppFig9) showing nice IFNAR1 stains in mouse MN and surrounding microglia.
3) My Comment Figure 6:
The authors removed the entire Figure, also due to comments of Referee#1. I do understand their move, but this is clearly a side-project to follow-up in future sudies (their system in Aging vs Neurodegeneration). It does NOT impact the findings of this study.
4) My Comment Figure 7:
They added a laser-microdissection exp. (Fig7C-F) to show canonical proteasome going down in MN of ALS mice while immunoproteasome going up. For my other comments about Fig7, I accept their explanations about possible differences between the Brenner study and their findings, especially as they actually repeated this exp. and showed it to the referee.
5) My Comment Title:
They adapted accordingly and added "models" to "ALS".

Response to the referees (2nd revision):

First of all, we again appreciate the considerable efforts that the referees have dedicated to our manuscript and are pleased to hear that they support its publication after some minor changes. In this response letter, we have addressed each comment point-by-point.

Referee #1:

The authors have responded to my comments in an exemplary manner. The new Figure 1, which establishes that cytoplasmic monomeric TDP-43 associates with TBK1 and promotes its phosphorylation, supplies the mechanistic starting point that the original manuscript lacked and completes the logic of the proposed axis. The newly established human iPSC-derived lower motor neuron model, the laser microdissection analysis of motor neurons from SOD1-G93A mice, the side-by-side comparison of autophagy and proteasome inhibition, and the demonstration that ER stress reduces IFNAR1 all strengthen the study considerably. I also wish to commend the authors for withdrawing the original Figure 6 rather than retaining an underdetermined claim, and for presenting their negative and inconclusive results transparently in the response. Notwithstanding these substantial improvements, several points remain, as described below.

Major points

1. Quantification of the new Figure 1

The proposition that monomeric TDP-43 activates TBK1 is now one of the central claims of the paper, yet the evidence for their association in Figure 1 is largely qualitative, resting on representative confocal images. The conclusion would be more persuasive if supported by a more quantitative presentation of the existing data. For Figure 1C, reporting the number of patients and control subjects examined, the number of motor neurons assessed, and the proportion showing colocalization would similarly strengthen the panel. Since the images have already been acquired, I anticipate that this can be addressed without additional experiments

Response:

We thank the referee for this suggestion. For Fig. 1C, we have performed quantitative analysis as suggested and incorporated the results and methodological details into Fig. 1C and the corresponding figure legend (lines 837–842).

For Fig. 1A and 1B, however, quantitative comparison between WT and 6M was less straightforward, as, unlike the near all-or-none colocalization pattern observed in Fig. 1C. WT also showed mild cytoplasmic mislocalization and colocalization with p-TBK1 in many cells under these overexpression conditions. Under such conditions, a simple positive/negative classification would not adequately

capture the extent of colocalization and could potentially mask the difference between WT and 6M. A more suitable quantitative measure, such as Peason's correlation coefficient, would require confocal images acquired under specific conditions that were not used for these images, precluding retrospective quantification.

Importantly, as an alternative quantitative approach to assess the association between TBK1 and TDP-43, we performed co-immunoprecipitation experiments in Fig. 1E–G. Quantification of these experiments demonstrated a statistically significant increase in the association of TBK1 with the monomeric TDP-43 mutant 6M compared with WT TDP-43. We acknowledge that these biochemical data do not directly substitute for quantitative imaging analysis; nevertheless, we believe that they provide complementary and objective quantitative evidence supporting the enhanced association between TBK1 and monomeric TDP-43.

2. Calibration of the claim of a physical interaction

At lines 91-92 the authors state that colocalization in patient spinal motor neurons provides the first in vivo evidence of a TDP-43-TBK1 interaction. However, colocalization at the resolution of confocal microscopy does not demonstrate an interaction, and the same reservation applies to the corresponding statement in the Abstract. Furthermore, DSP is a cell-permeable crosslinker that stabilises indirect as well as direct complexes, so the co-immunoprecipitation data in Figure 1E-G establish association within a complex rather than direct binding. The possibility that p-TBK1 is passively recruited into, or sequestered by, cytoplasmic TDP-43 assemblies is not formally excluded by the present data.

I do not regard this as requiring additional experiments, but the wording should be adjusted. I would suggest describing the human data as colocalization consistent with an association in vivo, and replacing "via a physical interaction" with "in association with" or "via a physical association" in the Abstract, Results, and Figure 8 legend. This would leave the authors' conclusion intact while placing it on secure ground.

Response:

We thank the referee for this careful comment and for suggesting alternative wording. We agree with the referee's concern and have replaced "interaction" with "association" throughout the relevant sections of the manuscript, including the Abstract (line 37), Results (lines 91 and 98), Discussion (line 264), and figure legends (lines 828, 830, and 1103).

We would, however, like to clarify several points regarding the possibility that TBK1 is passively recruited into or sequestered by cytoplasmic TDP-43 assemblies.

First, as described in the Methods section (lines 504–517), a soluble fraction extracted using a

relatively mild lysis buffer [50 mM Tris-HCl (pH 7.4), 150 mM NaCl, 2 mM $MgCl_2$, 0.5% (v/v) NP-40, and 1 mM dithiothreitol (DTT)] was used for the co-immunoprecipitation experiments. Given these mild extraction conditions, we consider it unlikely that insoluble cytoplasmic TDP-43 assemblies (i.e., aggregates) were efficiently solubilized. Thus, although our data do not establish direct binding between TDP-43 and TBK1, we believe that the co-immunoprecipitation results are consistent with an association occurring before the formation of insoluble TDP-43 aggregates.

Second, we would also like to point out that the TBK1 kinase inhibitor BX795 significantly reduced the association between TBK1 and TDP-43 in Fig. 1F and 1G. If p-TBK1 were just trapped within pre-existing cytoplasmic TDP-43 assemblies as a bystander, its association with TDP-43 would not be expected to depend on TBK1's own kinase activity. This finding therefore suggests that their association is regulated by an activity-dependent mechanism, rather than simply reflecting passive recruitment or sequestration of TBK1 into cytoplasmic TDP-43 assemblies, although the underlying mechanism remains unclear.

We nevertheless agree with the referee that the present data do not formally exclude such a possibility and have revised the wording throughout the manuscript as suggested.

Minor points

(i) In Figure 7J, the one-way ANOVA includes groups with n = 2 (WT and Tbk1+/−). The principal comparison between SOD1-G93A and SOD1-G93A/Tbk1+/− (n = 6 each) is unaffected, but the two-animal groups should be presented descriptively rather than entered into the statistical model, or the limitation should be stated in the legend.

Response:

The referee is correct, and we apologize for the inadequate statistical analysis in the original manuscript. In our initial submission, we performed a one-way ANOVA including the WT and Tbk1+/− groups (n = 2 each). We subsequently excluded these two groups from the statistical comparison in accordance with the journal guidelines but inadvertently failed to update the statistical test accordingly. We agree that an unpaired t-test would have been the appropriate approach to compare the SOD1-G93A and SOD1-G93A/Tbk1+/− groups. The exact P values obtained using an unpaired t-test with Welch's correction have now been provided in the revised manuscript (lines 1095–1096).

Importantly, this correction did not affect the statistical significance or our overall conclusions. Nevertheless, we apologize for this oversight and for any unnecessary concern it may have caused regarding the validity of the statistical analysis.

(ii) The Methods state that outliers were identified by Grubbs' test and excluded from all analyses. With group sizes of three to five, this procedure can appreciably influence the outcome. Please state how many data points were excluded and from which panels.

Response: We thank the referee for this comment and have added the requested information to the manuscript. Please refer to the "Statistical analysis" in the Method section (lines 618–619).

(iii) The linear mixed-effects analysis in Figure 5F is based on two control and three ALS donors. Estimation of a random effect from so few levels is unstable, and a brief acknowledgement of this in the legend or Methods would be appropriate. The analysis in Figure 5E, with four and six donors, is more secure.

Response: We thank the referee for the suggestion. We have explicitly acknowledged this limitation in the Fig. 5F legend (lines 1010–1012) as follows:

"Given the limited number of donors included in the analysis shown in Fig. 5F (two control and three ALS donors), estimation of the donor-level random effect may be unstable, and the results should therefore be interpreted with caution."

(iv) The demonstration that HA-tagged TDP-43 behaves comparably to the 3xFLAG construct (response to my minor point 1) is a useful control and would be better placed in the Appendix than restricted to the referees, since other readers will raise the same question.

Response: We thank the referee for the suggestion. We have incorporated this result into the manuscript (line 154) and the Appendix as Appendix Figure S7.

(v) I accept the authors' account of their unsuccessful attempts to characterise PSMB8 and IFNβ in postmortem tissue, and I do not ask for further work on this point. However, because the antibody screening is documented only in the response to referees, the Discussion should state explicitly that expression of the immunoproteasome and IFNβ in patient tissue remains uncharacterised and constitutes a limitation of the study.

Response: We thank the referee for your suggestion. We have described this limitation in the Discussion section (lines 356–358) as following;

"Additionally, the expression levels of immunoproteasome subunits and IFNβ in patient tissues remain uncharacterized, which constitutes a limitation of this study."

(vi) Line 533: "Eequal amounts".

Response: We thank the referee for the careful reading. This typo has been corrected, and we apologize for this oversight.

Lastly, we sincerely thank the referee for the many constructive comments and for carefully reading our manuscript. We acknowledge that our study has several limitations and have revised the manuscript accordingly. We hope that our responses above adequately address the referee's concerns.

Referee #2:

The authors have made a huge effort to respond to all the comments of the 3 reviewers.

Regarding my own comments (Referee#2), all my concerns have been adequately resolved by the authors and I thank them for the effort and additional experiments. I congratulate them for this excellent study that brings important new findings to the field.

Details about their responses - all accepted:

1) My Comment Figures 1/5:

They explained well were to find the asked for controls in the WBs and added a new SuppFig5 with side-by-side WBs showing the effects of blocking autophagy or proteasome in their system.

2) My Comment Figure 4:

They added a new IF (SuppFig9) showing nice IFNAR1 stains in mouse MN and surrounding microglia.

3) My Comment Figure 6:

The authors removed the entire Figure, also due to comments of Referee#1. I do understand their move, but this is clearly a side-project to follow-up in future sudies (their system in Aging vs Neurodegeneration). It does NOT impact the findings of this study.

4) My Comment Figure 7:

They added a laser-microdissection exp. (Fig7C-F) to show canonical proteasome going down in MN of ALS mice while immunoproteasome going up. For my other comments about Fig7, I accept their explanations about possible differences between the Brenner study and their findings, especially as they actually repeated this exp. and showed it to the referee.

5) My Comment Title:

They adapted accordingly and added "models" to "ALS".

Response:

We are pleased that the referee found our responses satisfactory. Thanks to the referee's questions and comments, we believe that this study has been substantially improved. We sincerely thank the referee once again for all the constructive feedback.

2nd Revision - Editorial Decision **18th Sep 2026**

Prof. Koji Yamanaka
Nagoya University Research Institute of Environmental Medicine
Neuroscience and Pathobiology
Furo-cho
Chikusa-ku
Nagoya, Aichi 464-8601
Japan

Dear Prof. Yamanaka,

I am very pleased to accept your manuscript for publication in the next available issue of EMBO reports. Thank you for your contribution to our journal.

Your manuscript will be processed for publication by EMBO Press. It will be copy edited and you will receive page proofs prior to publication. Please note that you will be contacted by Springer Nature Author Services to complete licensing and payment information.

You may qualify for financial assistance for your publication charges - either via a Springer Nature fully open access agreement or an EMBO initiative. Check your eligibility: https://link.springer.com/journal/44319/how-to-publish-with-us

Should you be planning a Press Release on your article, please notify the production team via this form in order to coordinate publication and release dates: https://forms.cloud.microsoft/e/eH4A8JVP6g

If you have any questions, please do not hesitate to contact the Editorial Office. Thank you for your contribution to EMBO Reports.

Yours sincerely,

Esther Schnapp, PhD
Senior Editor
EMBO reports

>>> Please note that it is EMBO Reports policy for the transcript of the editorial process (containing referee reports and your response letter) to be published as an online supplement to each paper. If you do NOT want this, you will need to inform the Editorial Office via email immediately. More information is available here: https://link.springer.com/partners/embo-press/editorial-policies#Peer%20review

EMBO Press Author Checklist

Corresponding Author Name: Koji Yamanaka	
Journal Submitted to: EMBO Reports	
Manuscript Number: EMBOR-2025-62894-T	

USEFUL LINKS FOR COMPLETING THIS FORM
The EMBO Journal - Author Guidelines
EMBO Reports - Author Guidelines
Molecular Systems Biology - Author Guidelines
EMBO Molecular Medicine - Author Guidelines

Reporting Checklist for Life Science Articles (updated January
This checklist is adapted from Materials Design Analysis Reporting (MDAR) Checklist for Authors. MDAR establishes a minimum set of requirements in transparent reporting in the life sciences (see Statement of Task: 10.31222/osf.io/9sm4x). Please follow the journal's guidelines in preparing your
Please note that a copy of this checklist will be published alongside your article.

Abridged guidelines for figures
1. Data
The data shown in figures should satisfy the following conditions:
- the data were obtained and processed according to the field's best practice and are presented to reflect the results of the experiments in an accurate and unbiased manner.
- ideally, figure panels should include only measurements that are directly comparable to each other and obtained with the same assay.
- plots include clearly labeled error bars for independent experiments and sample sizes. Unless justified, error bars should not be shown for technical
- if n<5, the individual data points from each experiment should be plotted. Any statistical test employed should be justified.
- Source Data should be included to report the data underlying figures according to the guidelines set out in the authorship guidelines on Data

2. Captions
Each figure caption should contain the following information, for each panel where they are relevant:
- a specification of the experimental system investigated (eg cell line, species name).
- the assay(s) and method(s) used to carry out the reported observations and measurements.
- an explicit mention of the biological and chemical entity(ies) that are being measured.
- an explicit mention of the biological and chemical entity(ies) that are altered/varied/perturbed in a controlled manner.
- the exact sample size (n) for each experimental group/condition, given as a number, not a range;
- a description of the sample collection allowing the reader to understand whether the samples represent technical or biological replicates (including how many animals, litters, cultures, etc.).
- a statement of how many times the experiment shown was independently replicated in the laboratory.
- definitions of statistical methods and measures:
  - common tests, such as t-test (please specify whether paired vs. unpaired), simple χ2 tests, Wilcoxon and Mann-Whitney tests, can be unambiguously identified by name only, but more complex techniques should be described in the methods section;
  - are tests one-sided or two-sided?
  - are there adjustments for multiple comparisons?
  - exact statistical test results, e.g., P values = x but not P values < x;
  - definition of 'center values' as median or average;
  - definition of error bars as s.d. or s.e.m.

Please complete ALL of the questions below.
Select "Not Applicable" only when the requested information is not relevant for your study.

Materials

Newly Created Materials	Information included in the manuscript?	In which section is the information available? (Reagents and Tools Table, Materials and Methods, Figures, Data Availability Section)
New materials and reagents need to be available; do any restrictions apply?	Yes	Reagents and Tools Table, Methods

Antibodies	Information included in the manuscript?	In which section is the information available? (Reagents and Tools Table, Materials and Methods, Figures, Data Availability Section)
For **antibodies** provide the following information:<br>- Commercial antibodies: RRID (if possible) or supplier name, catalogue number and or/clone number<br>- Non-commercial: RRID or citation	Yes	Reagents and Tools Table, Methods

DNA and RNA sequences	Information included in the manuscript?	In which section is the information available? (Reagents and Tools Table, Materials and Methods, Figures, Data Availability Section)
**Short novel DNA or RNA including primers, probes**: provide the sequences.	Yes	Reagents and Tools Table, Methods

Cell materials	Information included in the manuscript?	In which section is the information available? (Reagents and Tools Table, Materials and Methods, Figures, Data Availability Section)
**Cell lines:** Provide species information, strain. Provide accession number in repository **OR** supplier name, catalog number, clone number, and/**OR** RRID.	Yes	Reagents and Tools Table, Methods
**Primary cultures:** Provide species, strain, sex of origin, genetic modification status.	Yes	Methods
Report if the cell lines were recently **authenticated** (e.g., by STR profiling) and tested for mycoplasma contamination.	Not Applicable	

Experimental animals	Information included in the manuscript?	In which section is the information available? (Reagents and Tools Table, Materials and Methods, Figures, Data Availability Section)
**Laboratory animals or Model organisms:** Provide species, strain, sex, age, genetic modification status. Provide accession number in repository **OR** supplier name, catalog number, clone number, **OR** RRID.	Yes	Reagents and Tools Table, Methods
**Animal observed in or captured from the field:** Provide species, sex, and age where possible.	Not Applicable	
Please detail **housing and husbandry conditions**.	Yes	Methods

Plants and microbes	Information included in the manuscript?	In which section is the information available? (Reagents and Tools Table, Materials and Methods, Figures, Data Availability Section)
**Plants:** provide species and strain, ecotype and cultivar where relevant, unique accession number if available, and source (including location for collected wild specimens).	Not Applicable	
**Microbes:** provide species and strain, unique accession number if available, and source.	Not Applicable	

Human research participants	Information included in the manuscript?	In which section is the information available? (Reagents and Tools Table, Materials and Methods, Figures, Data Availability Section)
If collected and within the bounds of privacy constraints report on age, sex and gender or ethnicity for all study participants.	Yes	Appendix (Table S1)

Core facilities	Information included in the manuscript?	In which section is the information available? (Reagents and Tools Table, Materials and Methods, Figures, Data Availability Section)
If your work benefited from core facilities, was their service mentioned in the acknowledgments section?	Yes	Acknowledgments

Design

Study protocol	Information included in the manuscript?	In which section is the information available? (Reagents and Tools Table, Materials and Methods, Figures, Data Availability Section)
If study protocol has been **pre-registered, provide DOI in the manuscript**. For clinical trials, provide the trial registration number **OR** cite DOI.	Not Applicable	
Report the **clinical trial registration number** (at ClinicalTrials.gov or equivalent), where applicable.	Not Applicable	

Laboratory protocol	Information included in the manuscript?	In which section is the information available? (Reagents and Tools Table, Materials and Methods, Figures, Data Availability Section)
Provide DOI OR other citation details if **external detailed step-by-step protocols** are available.	Not Applicable	

Experimental study design and statistics	Information included in the manuscript?	In which section is the information available? (Reagents and Tools Table, Materials and Methods, Figures, Data Availability Section)
Include a statement about **sample size** estimate even if no statistical methods were used.	Yes	Methods (Statistical analysis)
Were any steps taken to minimize the effects of subjective bias when allocating animals/samples to treatment (e.g. **randomization procedure**)? If yes, have they been described?	Not Applicable	
Include a statement about **blinding** even if no blinding was done.	Not Applicable	
Describe **inclusion/exclusion criteria** if samples or animals were excluded from the analysis. Were the criteria pre-established?<br><br>If sample or data points were omitted from analysis, report if this was due to attrition or intentional exclusion and provide justification.	Yes	Methods (Statistical analysis)
For every figure, are **statistical tests** justified as appropriate? Do the data meet the assumptions of the tests (e.g., normal distribution)? Describe any methods used to assess it. Is there an estimate of variation within each group of data? Is the variance similar between the groups that are being statistically compared?	Yes	Methods (Statistical analysis)

Sample definition and in-laboratory replication	Information included in the manuscript?	In which section is the information available? (Reagents and Tools Table, Materials and Methods, Figures, Data Availability Section)
In the figure legends: state number of times the experiment was **replicated** in laboratory.	Yes	Figure legends
In the figure legends: define whether data describe **technical or biological replicates**.	Yes	Figure legends

Ethics

Ethics	Information included in the manuscript?	In which section is the information available? (Reagents and Tools Table, Materials and Methods, Figures, Data Availability Section)
Studies involving **human participants**: State details of **authority granting ethics approval** (IRB or equivalent committee(s), provide reference number for approval.	Yes	Methods (Postmortem human tissues & iPSC culture, differentiation, and transfection)
Studies involving **human participants**: Include a statement confirming that **informed consent** was obtained from all subjects and that the experiments conformed to the principles set out in the WMA Declaration of Helsinki and the Department of Health and Human Services Belmont Report.	Yes	Methods (Postmortem human tissues & iPSC culture, differentiation, and transfection)
Studies involving **human participants:** For publication of **patient photos**, include a statement confirming that consent to publish was obtained.	Not Applicable	
Studies involving experimental **animals**: State details of **authority granting ethics approval** (IRB or equivalent committee(s), provide reference number for approval. Include a statement of compliance with ethical regulations.	Yes	Methods (Animals)
Studies involving **specimen and field samples:** State if relevant **permits** obtained, provide details of authority approving study; if none were required, explain why.	Not Applicable	

Dual Use Research of Concern (DURC)	Information included in the manuscript?	In which section is the information available? (Reagents and Tools Table, Materials and Methods, Figures, Data Availability Section)
Could your study fall under dual use research restrictions? Please check biosecurity documents and list of **select agents and toxins** (CDC): https://www.selectagents.gov/sat/list.htm	Not Applicable	
If you used a select agent, is the security level of the lab appropriate and reported in the manuscript?	Not Applicable	
If a study is subject to dual use research of concern regulations, is the name of the **authority granting approval and reference number** for the regulatory approval provided in the manuscript?	Not Applicable	

Reporting

The MDAR framework recommends adoption of discipline-specific guidelines, established and endorsed through community initiatives. Journals have their own policy about requiring specific guidelines and recommendations to complement MDAR.

Adherence to community standards	Information included in the manuscript?	In which section is the information available? (Reagents and Tools Table, Materials and Methods, Figures, Data Availability Section)
State if relevant guidelines or checklists (e.g., **ICMJE, MIBBI, ARRIVE, PRISMA**) have been followed or provided.	Not Applicable	
For **tumor marker prognostic studies,** we recommend that you follow the **REMARK** reporting guidelines (see link list at top right). See author guidelines, under 'Reporting Guidelines'. Please confirm you have followed these guidelines.	Not Applicable	
For **phase II and III randomized controlled trials**, please refer to the **CONSORT** flow diagram (see link list at top right) and submit the CONSORT checklist (see link list at top right) with your submission. See author guidelines, under 'Reporting Guidelines'. Please confirm you have submitted this list.	Not Applicable	

Data Availability

Data availability	Information included in the manuscript?	In which section is the information available? (Reagents and Tools Table, Materials and Methods, Figures, Data Availability Section)
Have **primary datasets** been deposited according to the journal's guidelines (see 'Data Deposition' section) and the respective accession numbers provided in the Data Availability Section?	Not Applicable	
Were **human clinical and genomic datasets** deposited in a public access-controlled repository in accordance to ethical obligations to the patients and to the applicable consent agreement?	Not Applicable	
Are **computational models** that are central and integral to a study available without restrictions in a machine-readable form? Were the relevant accession numbers or links provided?	Not Applicable	
If publicly available data were reused, provide the respective **data citations in the reference list**.	Yes	Results, References, Appendix (Figure S8)
